# Proteomic Analysis of Proteins Responsive to Drought and Low Temperature Stress in a Hard Red Spring Wheat Cultivar

**DOI:** 10.3390/molecules25061366

**Published:** 2020-03-17

**Authors:** Maryke Labuschagne, Stefania Masci, Silvio Tundo, Vera Muccilli, Rosaria Saletti, Angeline van Biljon

**Affiliations:** 1 Department of Plant Sciences, University of the Free State, Bloemfontein 9300, South Africa; avbiljon@ufs.ac.za; 2 Department of Agricultural and Forestry Sciences, University of Tuscia, 01100 Viterbo, Italy; masci@unitus.it; 3 Department of Land, Environment, Agriculture and Forestry (TESAF), Research Group in Plant Pathology, University of Padova, Viale dell’Università 16, 35020 Legnaro (PD), Italy; silvio.tundo@unipd.it; 4 Department of Chemical Sciences, Organic Mass Spectrometry Laboratory, University of Catania, 95131 Catania, Italy; v.muccilli@unict.it (V.M.); rsaletti@unict.it (R.S.)

**Keywords:** abiotic stress, bread wheat, glutenin, proteomics

## Abstract

Drought stress is becoming more prevalent with global warming, and has been shown to have large effects on gluten proteins linked to wheat bread making quality. Likewise, low temperature stress can detrimentally affect proteins in wheat. This study was done to determine the differential abundance of high molecular weight (HMW) glutenin proteins in a drought and low temperature stressed high quality hard red spring wheat cultivar (PAN3478), against a control. The treatments were applied in the greenhouse at the soft dough stage. HMW glutenin proteins were extracted from the flour, and were separated by using two-dimensional gel electrophoresis. Protein spots that had *p* values lower than 0.05 and fold values equal to or greater than 1.2 were considered to be significantly differentially abundant. These proteins were further analyzed by using tandem mass spectrometry. There was a 1.3 to 1.8 fold change in 17 protein spots due to the cold treatment. The drought treatment caused a 1.3 to 3.8 fold change in 19 protein spots. These spots matched either HMW or low molecular weight (LMW) glutenin subunits. In the latter case, the C subunits of LMW glutenins were notably found to be up-regulated under both stress conditions. All the proteins that have been identified can directly influence dough characteristics. Data are available via ProteomeXchange with the identifier PXD017578.

## 1. Introduction

In the last few years, there has been significant progress in protein separation and identification techniques, including two dimensional gel electrophoresis (2-DE), liquid chromatography, mass spectrometry (MS) and also in the availability of databases and database searching [1]. Among the most commonly used methods in proteomics, 2-DE allows easy resolution and visualization of thousands of protein species on a single gel, thus resolving proteoforms [2]. Expression proteomics are used to study quantitative and qualitative expression of proteins under different conditions [3]. Proteomics can be used to understand how the genome regions are involved with grain protein composition, the involvement of enzymes and the expression of specific genes in different growing conditions [4,5]. In this way, proteomics in wheat are proving to be a powerful tool to elucidate the expression of proteins and how they contribute to the value of the grain. 

Gluten proteins confer viscoelasticity to dough, which determines its suitability for specific end-use products. Gluten consists of glutenin and gliadin, which are characterized by high numbers of allelic polymorphisms. The relative amounts of composition of the glutenins and gliadins play a large role in dough characteristics and end-use quality. Glutenins confer elasticity, while gliadins confer viscosity [6]. Gliadins are monomeric and glutenins are polymeric proteins. Glutenins consist of high molecular weight (HMW) glutenin subunits (GS) and low molecular weight glutenin subunits (LMW-GS). The glutenins are linked by inter and intrachain disulfide bonds [7]. 

Drought stress is becoming more prevalent with global warming, and has been shown to have large effects on the breadmaking quality of wheat [8]. Likewise, low temperature stress detrimentally affects proteins in wheat. Proteins denature under low temperature stress, causing cellular damage. Low temperature tolerance is a multigenic trait that activates a large number of cold inducible genes [9,10]. Wheat is also very sensitive to heat stress, especially in the grain filling stage [11]. Proteomics approaches such as two dimensional gel electrophoresis (2-DE) can be done through the densitometric analysis of gels on which proteins have been separated. Spot intensity differences in replicate gels of samples are compared. The protein within the spots that have changed can be digested to peptides, which can further be analyzed with mass-spectrometric methods. This allows identification of proteoforms that were altered [12]. The proteins are identified by matching the spectra to theoretical data from a protein database [13]. 

Integration of functional genomics, proteomics, bioinformatics, breeding and genetic resources is aiding the understanding of the genetic and biochemical bases of quality traits in wheat. This information must be incorporated into breeding programs together with high throughput screening techniques to combine good yield and agronomic characteristics with good quality [14]. Proteomics tools can be used to investigate gluten proteins and to identifying specific proteins that are related to baking quality characteristics, for improvement of quality and functional properties, for improved understanding of structure and interrelationships as well as to reduce allergies [15].

Quantitative 2-DE was reported as a precise way to study ratios of HMW and LMW glutenins and to identify components in the polymer [16]. This can be coupled with tandem mass spectrometry (MS/MS), which identifies individual proteins that can contribute to glutenin polymer formation and flour quality [17].

The aim of this study was to determine how drought and low-temperature stress influence HMW and LMW glutenin proteins in a bread wheat cultivar with excellent bread making quality, as separated by 2-DE, and to identify the subunits that were significantly changed due to stress conditions using LC-MS/MS. 

## 2. Results

**Figure 1:**
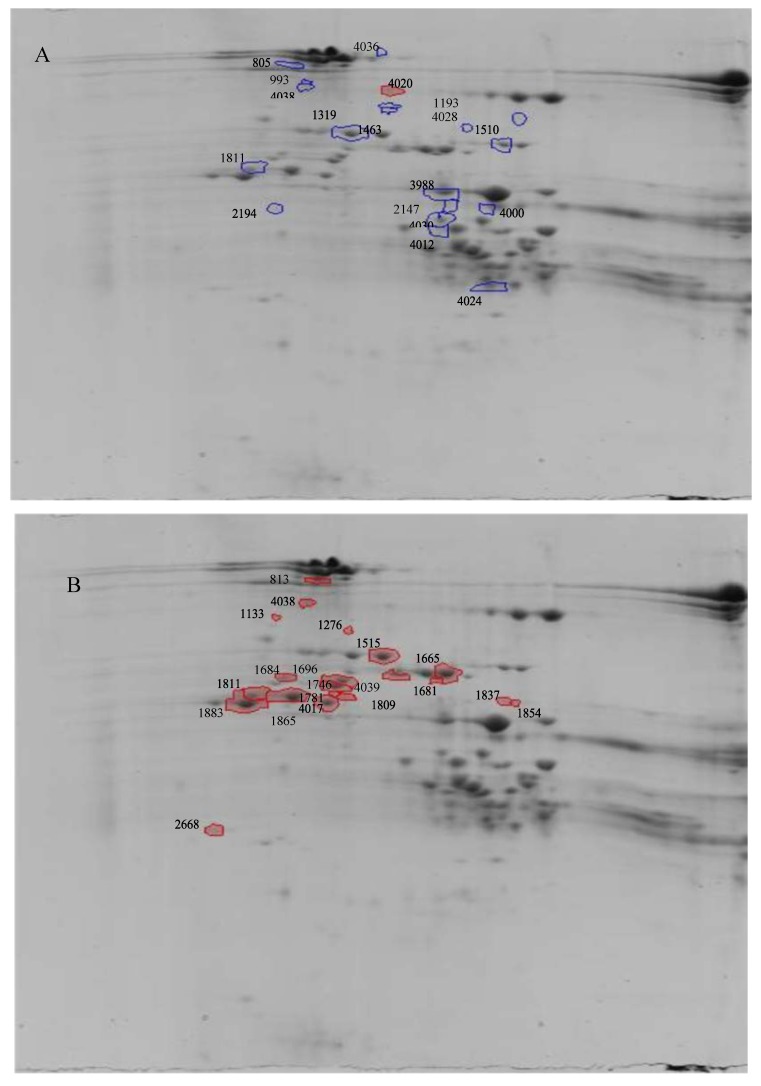
Differentially abundant proteins after (**A**) Cold treatment and (**B**) Drought treatment with spot numbers, as indicated in Table 1.

In the cold treatment, 17 spots were identified which were significantly (*p* ≤ 0.05) up-regulated due to cold stress (Figure 1A and Table 1). The increase varied between 1.3 to 1.8 fold changes. The drought treatment caused 1.3 to 3.8 fold change in 19 spots that were found to be significantly differentially abundant. All the spots that were significantly different between treatments, were increased due to the stress treatments, and none were reduced due to the treatments (Supplementary Figure S1). The reason for this is not clear.

After LC-MS/MS analysis, two spots, one with matching peptide sequences to HMW glutenin proteins Dx2, Dy12 and PC256, and one to HMW glutenin protein PW212, were found to be differentially abundant under both drought and cold stress (Table 1). PAN3478 has a HMW glutenin subunit composition of 1Ax1, 1Bx13, 1By16, 1Dx2 and 1Dy12 as separated with sodium dodecyl sulfate polyacrylamide gel electrophoresis (SDS-PAGE, data not shown). Only protein spots that were identified are reported in Table 1.

Proteins with peptide sequence similarity to α- and γ-gliadin were identified in this analysis, which is due to the presence of the C subunits of LMW glutenins [18,19]. In fact, monomeric gliadins were removed from all samples before glutenin extraction. In particular, three spots corresponding to α-type sequences and two to γ-type gliadins were up-regulated under cold-stress conditions, whereas one spot with α-gliadin type peptide sequence was up-regulated under drought stress. No proteins similar to ω-gliadin (D subunits of LMW glutenins) [20] were present amongst the spots that were found to be differentially abundant. In two spots, classical LMW glutenins (B subunits) [21] were also differentially abundant under cold-stress conditions (Table 1). 

## 3. Discussion

The processing properties of wheat are largely determined by gluten proteins. The HMW glutenins are known to have a large effect on dough technological properties [22], and clearly proteins with peptide sequences that are similar, especially HMW glutenins DY12, PW212, PC256, HMW-GS DX2 and DY12, were responsive to cold and drought stress, and could directly influence dough characteristics.

LMW glutenins also play a role in dough matrix formation. LMW glutenins are classically subdivided into three groups: B, C and D, with only the former corresponding to classical LMW glutenins, whereas the latter two correspond to proteins that are structurally gliadins, but functionally glutenins. In particular, C subunits have α- and γ-gliadin type sequences, whereas D subunits have ω-gliadin type sequences. Their occurrence in the glutenin fraction is due to the presence of an uneven number of cysteines that affects their polymerization behavior, enabling them to form intermolecular disulfide bonds [18,19,20]. The role of these subunits on gluten quality is contrasting. Meanwhile B subunits, which are classical LMW glutenins, are considered to be glutenin chain extenders because of the presence of two cysteine residues available in forming intermolecular disulfide bonds, and thus have a positive effect on polymer size and, consequently, dough quality. The C and D subunits are chain terminators, as reviewed previously [21].

Wheat production is significantly affected by abiotic stresses, such as high temperatures at grain filling. Heat and drought stress affects grain protein synthesis, cellular and carbohydrate metabolism, as well as enzymes involved in transcription and translation, and thus disrupts grain development. In a study using iTRAQ, 256 differential abundant proteins were seen between normal and high temperatures. LMW glutenins were significantly reduced at 15 and 20 DPA under heat stress [11]. Another study [23] reported on the effect of heat and drought stress applied at two stages of grain filling post-anthesis, using 2-DE and MS. The type and time of stresses affected the synthesis of individual protein fractions. Albumins were significantly reduced due to stress. Glutenin accumulation increased by 85%–159% during grain filling in response to stress. Combined stress conditions caused a significantly larger effect than single stress events. The α- and γ-type gliadin fractions decreased due to drought stress. In the current study, proteins similar to α-, and γ gliadins, but corresponding to C subunits of low molecular weight glutenins, were up-regulated under cold-stress conditions. However, in the case of drought stress, with only one spot corresponding to with α-type C subunit, was found under drought stress conditions, suggesting that these gliadin like subunits were more sensitive to cold stress conditions. Modulation of C subunits under stress can influence dough quality characteristics as a consequence of glutenin polymer size.

In another study [24] three wheat cultivars were subjected to drought stress during grain filling, followed by 2-DE and MALDI-TOF-TOF analysis of mature grains. A significant albumin and gliadin increase was seen under drought stress. The study identified 14 differentially abundant proteins of which eight were identified as potential complex forming proteins. The LMW glutenins did not show any significant reaction to drought stress, while only two up-regulated spots contained LMW glutenins under cold stress. 

When proteomic techniques are applied to investigate gluten proteins, it becomes clear that their characterization is very difficult and challenging. Indeed, it should be considered that: (1) sequence coverage of glutenin subunits will obviously be scarce owing to the long repeating motif lacking tryptic cleavage sites; (2) the few peptides with masses suitable for MS/MS fragmentation (occurring in the N-*terminal* and C-terminal domains) would not be able to discriminate between the single subunits because they are common to this kind of proteins; (3) a single 2D-gel spot may frequently contain more proteins belonging to the same group of gluten proteins, differing by point substitutions, insertion or deletion of short sequences in the repetitive domain.

In the current study, the HMW glutenins with similar peptide sequences to the D genome coded glutenins in particular (Dx2 and Dy12) were sensitive to cold and drought stress, as well as some LMW glutenins of B and C types. As these subunits are known to influence baking quality, their regulation would certainly affect baking quality characteristics. It is interesting to note that all the proteins identified resulted in up-regulation under stress conditions, although we do not have a rationale for this.

To conclude, proteomics is a useful tool for analyzing cereal grain protein. Many proteomics studies were aimed at generating knowledge on improvement of crop quality in terms of biotic and abiotic stress, and in terms of nutritional and processing quality [25]. Proteomics has the potential to shed light on genotypes through environment interaction in terms of wheat baking quality [26], and can serve as a powerful tool to elucidate the expression of proteins and how they contribute to the value of the grain. Interest in cereal proteomics goes beyond the elucidation of structure and function relationships, and studies can contribute to insights into quality because it is influenced by stress, pathogens and yield [27]. 

## 4. Materials and Methods

A commercial hard red spring wheat irrigation cultivar (PAN3478), was used for the study, as it has excellent baking quality characteristics. It was planted in 3 single pots filled with soil, in the greenhouse. Three treatments were applied to 15 pots per replication, three replications and three plants per pot. A randomized complete block design with two factors, treatments and cultivars, was used. Greenhouse temperatures were set at 15 °C/22 °C (night/day). Fertilization was applied to assure optimal growing conditions. Optimal watering of the pots was done throughout the experiment for the cold stress regime and the control, and up to the soft dough stage for the drought stress experiment. As soon as the main tillers in each pot reached the soft dough stage, treatments commenced. The soft dough stage is when wheat kernels contain approximately 50% moisture and is classified as a value of 85 on the Zadoks scale [28]. The main tiller of each plant was marked with a tag before treatment commenced. For the cold treatment, plants were placed in climate cabinets in the following cycle: 5 °C for 30 min then 1 °C less every 30 min until it reached −5.5 °C; then it was left for three hours after which it was increased to −2 °C for 30 min; then 0 °C for 30 min; then 2 °C for 30 min; then 5 °C for 30 min; then back to green-house to optimal conditions. This treatment was structured in a way to closely resemble field conditions in the spring wheat planting areas where cold spells are often experienced after anthesis. To induce drought stress, watering was withheld until severe wilting was visible and then watering was resumed. The control treatment was left in the green-house under optimal conditions prior to harvesting. At harvesting, the seed of the marked main tillers of the plants of the 15 pots per replication were bulked for each of the treatments and cultivars. The seed was milled to a whole flour with a laboratory mill (IKA A10 Yellowline analysis grinder, Merck Chemicals Pty Ltd, Mountainview, CA, USA). 

### 4.1. Glutenin Subunits Extraction and 2D Electrophoretic Analysis 

Samples of 100 mg of wheat flour from the control sample and from plants subjected to cold and drought stress were washed three times with 1 ml of 50% (*v*/*v*) 1-propanol in order to remove gliadins [29]. The pellet was treated with a solution (1:10) composed of 50% 1-propanol, 50 mM tris-HCl pH 8.8, 1% dithiothreitol (DTT), 1 mM EDTA, 10 mM iodoacetamide for 1 h at 65 °C. After centrifugation (13000 rpm, 10 min), the proteins were precipitated overnight at −20 °C in 1 ml of cold acetone and centrifuged. The precipitated proteins were further rinsed with cold acetone and dried. The protein concentration was quantified using a BCA protein assay kit.

The dried pellets, corresponding to about 400 µg proteins, were dissolved in 393 µl of strip rehydration buffer (7 M Urea, 2 M thiourea, 2% Chaps, 2% Triton X-100) adding 1.2% (*v*/*v*) destreak reagent (GE Healthcare, Chicago, IL, USA) and 0.5% IPG buffer pH 3-10 at the time of use. IEF linear IPG strips 18 cm, pH 3–10 (GE Healthcare) that were rehydrated in 16 h at room temperature were used. After the rehydration step, focusing was performed at 20 °C for 36 kVh (500 V 3 h, 1,000 V 1 h gradient, 10,000 V 3 h gradient, 10,000 V 2 h) using the IPGphor^TM^Isoelectric Focusing System (GE Healthcare) with the manifold support. The gel strips were equilibrated for 45 min in 0.05 M tris-HCl pH 8.8, 6M urea, 30% (*w/v*) glycerol, 2% (*w/v*) SDS and 2 mg mL^−1^ of bromophenol blue as the tracking dye.

For the second dimension, the strips were placed on 18 × 20 cm polyacrylamide gels (T = 12%, C = 2.67%) of 1 mm thickness (Protean Plus multi-casting chamber, Bio-Rad; Protean Plus Dodeca Cell, Bio-Rad) and run at 40 mA per gel for 5–6 h at 10 °C until the dye front left the gel.

Twenty seven gels were performed in total, corresponding to three biological and three technical replicates for each sample (thus nine from the control sample, and nine from each of the treated samples (cold and drought stress). Averaged normalized volumes for nine gels were used for each spot for each treatment. The raw data is presented in the Supplementary data. Gels were highly repeatable. 

### 4.2. Protein Staining, Image Acquisition and Gel Spots Excision

In order to quantitatively compare the proteins present in each of the gels, they were stained overnight with Coomassie Brilliant Blue G-250 (Sigma-Aldrich, St. Louis, MO, USA) [30], then destained for 4 h in distilled water and scanned at 300 dot per inch and 16-bit grayscale pixel depth. Gels were analyzed with the software SameSpots Progenesis (version 4.6.1.218, Nonlinear Dynamics, UK). Those protein spots that had *p* value, obtained from analysis of variance (ANOVA), lower than 0.05 and fold value equal to or greater than 1.2 were considered as significant differentially abundant. Principal Components Analysis (PCA) was done on the data [31]. Following this analysis, differential abundance spots were selected from those gels in which the spot color was more intense, that is, where the protein concentration was higher and transferred into 1.5 ml tubes. The excision of the spots was carried out by using an EXQuestTM Spot Cutter of Bio-Rad. Spots were subsequently analyzed and identified by liquid chromatography tandem-mass spectrometry (LC-MS/MS).

### 4.3. In-gel Digestion and Mass Spectrometric Analysis

Selected protein spots from the 2-DE gels were excised manually and transferred to 1.5 ml microcentrifuge tubes. The spots were then washed and subjected to in-gel trypsin digestion and subsequently to mass spectrometric analysis [32,33]. After soaking trypsin (modified porcine trypsin, Promega, Milan, Italy) into the gel pieces, the supernatant containing excess trypsin was removed and the gel pieces were covered with 50 μl of 50 mM NH_4_HCO_3_ and incubated at 37 °C overnight. The enzymatic reaction was stopped by cooling the gel pieces and the supernatant solution at −20 °C. After in-gel digestion, the peptides solutions were transferred into clean 0.5 ml tubes. The peptides were extracted from gel pieces with 40 µl of 0.5% formic acid (FA) and subsequently with the same volume of acetonitrile. This extraction procedure was repeated three times. The total extracts were pooled, combined with the first supernatant, lyophilized and dissolved in 20 µl of 0.5% FA. Capillary RP-HPLC/nESI-MS/MS was performed using an Ultimate 3000 LC system combined with an autosampler and a flow splitter 1:100 (Dionex Corporation, Sunnyvale, CA, USA), coupled on-line with a linear ion trap nano-electrospray mass spectrometer (LTQ, Thermo Fischer Scientific, San Jose, CA, USA). Ionization was performed with a liquid junction using an uncoated capillary probe (30 ± 2 µm i.d.; New Objective, Woburn, MA, USA). 

The peptide solution was loaded onto a C18 μ-pre-column cartridge (0.3 mm × 5 mm, 100 Å, 5 μm, PepMap, Dionex) equilibrated with 0.5% aqueous FA at a flow rate of 20 μL min^−1^ for 4 min. Subsequently, the solution was switched onto a reversed-phase C18 column (0.18 mm × 150 mm, 300 Å, 5 μm, BioBasic, ThermoFisher Scientific, Waltham, MA, USA) and peptides were separated by elution at room temperature with a linear gradient of solvent B (CH_3_CN + 0.5%) in A (H_2_O + 0.5% FA) from 10% to 50% in 50 min at a flow rate of 1.5 μL min^−1^. HPLC-grade water and CH_3_CN were provided by Carlo Erba (Milan, Italy). The nESI source was operated under the following conditions: a capillary temperature of 220 °C and spray voltage of 1.9 kV. Repetitive mass spectra were acquired in positive ion mode in the *m/z* range 250–2000.

Characterization of peptide ions was performed by the data-dependent method as follows: (i) full scan MS in the *m/z* range 250–2000; (ii) zoom scan of the three most intense ions (isolation width: 2 Da); (iii) MS/MS analysis of the three most intense ions (normalized collision energy; 30 a.u., activation Q: 0.250). Mass calibration was made using a standard mixture of caffeine (M_r_ 194.1 Da), MRFA peptide (M_r_ 523.6 Da) and Ultramark (M_r_ 1621 Da). Data acquisition was performed using the Excalibur v. 1.4 software (ThermoFisher Scientific).

### 4.4. Database Search and Protein Identification

LC–MS/MS data were processed by PEAKS software v. 8.5 (Bioinformatics Solutions Inc., Waterloo, ON, Canada). Data were searched against the “*Viridiplantae*” SwissProt database with 40,218 sequences, and TrEMBL with 9,314,329 sequences (release November 2019). Full tryptic peptides with a maximum of three missed cleavage sites were subjected to a bioinformatics search.

Cysteine carboxyamidomethylation was set as fixed modification, whereas oxidation of methionine, and transformation of N-*terminal* glutamine and N-*terminal* glutamic acid residue in the pyroglutamic acid form were included as variable modifications.

The precursor mass tolerance threshold was ± 1.2 Da and a tolerance of ± 0.6 Da for the fragment ions was set. Peptide spectral matches (PSMs) were validated using Target Decoy PSM Validator node based on q-values at a 1% FDR. A protein was considered identified with a minimum of 2 peptides; proteins that contained similar peptides and could not be differentiated based on MS/MS analysis alone were grouped to satisfy the principles of parsimony. In the case of gliadins and some LMW- and HMW-GS, they were considered to have also been identified with a single peptide and its MS/MS spectrum was manually verified for the purpose of supporting this identification. This derogation from the number of peptides for the identifications was applied because of the peculiarity of the amino acid sequence. The mass spectrometry proteomics data were deposited to the ProteomeXchange Consortium via the PRIDE [34] partner repository with the dataset identifier PXD017578.

## Figures and Tables

**Table 1:** Protein spots that were changed due to cold and drought stress, and their identified peptides.

Treatment	Spot number	P value	Q Value	Power	Fold Change	Protein Identification	Acc.No	Score; Coverage (%)	Identified Peptides	M + H*; z
Cold stress	4020	0.008	0.82	0.811	1.7	HMW PW212	P08489	209.1; 7	GGSFYPGETTPPQQLQQR IFWGIPALLK QpyroSGQGQQGYYSSYHVSVEHQAASLK SLQQTGQGQQSGQGQQGYYSSYHVSVEHQAASLK	1989.9; 2 1156.7; 2 2721.2; 2 3665.7; 2
	4020					HMW 12	P08488	173.9; 6	LPWSTGLQMR LPWSTGLQMoxR QVVDQQLAGR QGYDSPYHVSAEQQAASPMoxVAK QpyroVVDQQLAGR	1187.6; 2 1203.6; 2 1112.6; 2 2379.1; 2 1095.6; 2
	4000	0.017	0.82	0.703	1.3	γ-gliadin	P08453	81.15; 4	APFASIVAGIGGQ	1186.6; 2
	3988	0.018	0.82	0.698	1.3	LMW	P10385	87.7; 3	QpyroIPEQSRHESIR QpyroIPEQSR PEQSRHESIR	1461.7; 2 839.4; 2 1237.6; 2
	4038					HMW 12	P08488	147.5; 3	LPWSTGLQMR LPWSTGLQMoxR QVVDQQLAGR	1187.6; 2 1203.6; 2 1112.6; 2
	4038					Fragment HMW PC256	P02861	107.5; 30	LEGGDALLASQ QWLQPR AQQLAAQLPAMoxCR	1072.5; 2 826.4; 2 1472.7; 2
	1463	0.022	0.82	0.666	1.8	HMW 12	P08488	203.1; 12	QpyroGYDSPYHVSAEQQAASPMoxVAK SLQQPGQGQQIGK LPWSTGLQMR QGYDSPYHVSAEQQAASPMoxVAK QGYYPTSLQQPGQGQQIGQGQQGYYPTSPQHTGQR LPWSTGLQMoxR	2362.0; 2 1367.7; 2 1187.6; 2 2379.1; 2 3862.8; 3 1203.6; 2
	1193	0.029	0.82	0.614	1.6	HMW 12	P08488	217.6; 14	LPWSTGLQMR SVAVSQVAR LPWSTGLQMoxR QVVDQQLAGR QpyroVVDQQLAGR QYEQTVVPPK QGYDSPYHVSAEQQAASPMoxVAK LVLFAAVVIALVALTTAEGEASR EpyroLQESSLE	1187.6; 2 915.5; 2 1203.6; 2 1112.6; 2 1095.6; 2 1187.6; 2 2379.0; 3 2313.3; 3 915.4 ;2
	1193					HMW PW212	P08849	94.3; 2	GGSFYPGETTPPQQLQQR	1989.9; 2
	4028	0.031	0.82	0.606	1.6	HMW PW212	P08849	71.8; 2	GGSFYPGETTPPQQLQQR	1989.9; 2
	1811	0.032	0.82	0.595	1.4	HMW PW212	P08849	187.8; 2	GGSFYPGETTPPQQLQQR ELQELQER	1989.9; 2 1043.5; 2
	2147	0.033	0.82	0.590	1.3	γ-gliadin	P8453	97.14; 4	APFASIVAGIGGQ	1186.6; 2
	2147					HMW PW212	P08849	187.8; 3	GGSFYPGETTPPQQLQQR	1989.9; 2
	2194	0.034	0.82	0.587	1.5	HMW PW212	P08849	1164.1; 3	GGSFYPGETTPPQQLQQR IFWGIPALLK	1989.9; 2 1156.7; 2
	2194					LMW PTDUCD1	P16315	48.3; 3	QLPQIPEQSR	1194.6
	1319	0.034	0.82	0.584	1.5	HMW 12	P08488	204.4; 15	QpyroGYYPTSLQQPGQGQQIGQGQQGYYPTSPQHTGQR QGYDSPYHVSAEQQAASPMoxVAK QpyroVVDQQLAGR LPWSTGLQMR EpyroQQAASPMVAKAQQPATQLPTVCRMEGGDALSASQ QpyroGYDSPYHVSAEQQAASPMoxVAK LPWSTGLQMoxR	3845.7; 3 2379.1; 3 1095.6; 3 1187.6; 2 3637.7; 3 2362.1; 2 1203.6; 2
	4012	0.044	0.82	0.537	1.4	α/β-gliadin A-IV	P04724	101.1; 12	GSVQPQQLPQFEEIR QpyroLPQFEEIR DVVLQQHSIAHGSSQVLQQSTY SVQPQQLPQFEEIR	1754.8; 2 1141.6; 2 2424.2; 3 1697.8; 2
	4024	0.046	0.82	0.531	1.3	α/β-gliadin	P02863	232.4; 21	PSQQNPQAQGSVQPQQLPQFEEIR SVQPQQLPQFEEIR GSVQPQQLPQFEEIR PQQLPQFEEIR QQQQPSSQVSFQQPLQQYPLGQGSFR PQFEEIR AIILHQQQK	2733.3; 3 1697.8; 2 1754.8; 2 1383.7; 3 2990.5; 3 917.4; 1 1077.6; 2
	4024					α/β-gliadin clone PTO-A10	P04728	63.1; 14	QQQQPSSQFSFQQPLQQYPLGQGSFR	3038.5; 3
	4024					γ-gliadin	P08453	92.9; 5	GIIQPQQPAQLEAIR	1660.9; 2
	1510	0.047	0.82	0.526	1.3	HMW PW212	P08849	100.6; 3	GGSFYPGETTPPQQLQQR ELQELQER	1989.9; 2 1043.5; 2
Drought stress	1883	0.003	0.74	0.907	1.6	HMW PW212	P08849	110.4; 2	GGSFYPGETTPPQQLQQR	1989.9; 2
	1811	0.003	0.74	0.902	1.7	HMW PW212	P08849	187.3; 2	GGSFYPGETTPPQQLQQR ELQELQER	1989.9; 2 1043.5; 2
	1865	0.006	0.74	0.850	1.6	HMW PW212	P08849	98.3; 2	GGSFYPGETTPPQQLQQR	1989.9; 2
	4017	0.006	0.74	0.843	1.4	HMW PW212	P08849	120.2; 3	GGSFYPGETTPPQQLQQR	1989.9; 2
	4039	0.016	0.74	0.718	1.5	Eukaryotic translation initiation factor isoform 4G-2	Q41583	37.7; 5	NGRNAPGGPLSPGGFS FDLLKGELLDSGITTADILKDVISLIF	1483.7; 2 2948.6; 3
	1854	0.025	0.74	0.641	1.5	HMW 12	P08488	134.3; 3	LPWSTGLQMoxR LPWSTGLQMR QYEQTVVPPK	1203.6; 2 1187.6; 2 1187.6; 2
	1854					α/β-gliadin A-II	P04722	70.8; 3	LWQIPEQSR	1155.6; 2
	1133	0.027	0.74	0.630	1.4	HMW PW212	P08489	79.7; 2	GGSFYPGETTPPQQLQQR	1989.9; 2
	1781	0.027	0.74	0.628	1.6	HMW PW212	P08489	100.7; 2	GGSFYPGETTPPQQLQQR	1989.9; 2
	4038					HMW 12	P08488	147.5; 3	LPWSTGLQMR LPWSTGLQMoxR QVVDQQLAGR	1187.6; 2 1203.6; 2 1112.6; 2
	4038					Fragment HMW PC256	P02861	107.5; 30	LEGGDALLASQ QWLQPR AQQLAAQLPAMoxCR	1072.5; 2 826.4; 2 1472.7; 2
	1809	0.031	0.74	0.600	1.7	HMW PW212	P08849	148.9; 3	GGSFYPGETTPPQQLQQR IFWGIPALLK	1989.9; 2 1156.7; 2
	1696	0.033	0.74	0.592	1.6	HMW PW212	P08489	98.3; 2	GGSFYPGETTPPQQLQQR	1989.9; 2
	1681	0.036	0.74	0.575	1.4	HMW PW212	P08849	113.4; 6	GGSFYPGETTPPQQLQQR MoxAKRLVLFVAVVVALVALTVAEGEASEQLQCER	1989.9; 2 3614.9; 3
	1681					HMW 12	P08488	24.9; 5	QpyroGYYPTSLQQPGQGQQIGQGQQGYYPTSPQHTGQR	3845.7; 3
	1684	0.043	0.74	0.544	1.3	HMW PW212	P08849	187.3; 3	GGSFYPGETTPPQQLQQR IFWGIPALLK ELQELQER	1989.9; 2 1156.7; 2 1043.5; 2
	1515	0.046	0.74	0.531	1.4	HMW PW212	P08489	174.9; 4	GGSFYPGETTPPQQLQQR	1989.9; 2
	1837	0.049	0.74	0.519	1.6	Plasma membrane ATPase	P83970	36.9; 6	EpyroMSALYLQVSIVSQALIFVT EpyroMoxSALYLQVSIVSQALIFVT LGMoxGTNMYPSSALLGQSK LGDIVPADARLLEGDPLK	2193.1; 2 2209.1; 2 1869.9; 2 1891.7; 2
	1665	0.049	0.74	0.516	1.3	HMW PW212	P08849	147.9; 4	GGSFYPGETTPPQQLQQR	1989.9; 2
Cold and drought stress	1811	0.003	0.61	0.901	1.7	HMW PW212	P08849	187.3; 3	GGSFYPGETTPPQQLQQR ELQELQER	1989.9; 2 1043.5; 2
	4038					HMW 12	P08488	147.5; 3	LPWSTGLQMR LPWSTGLQMoxR QVVDQQLAGR	1187.6; 2 1203.6; 2 1112.6; 2
	4038					Fragment HMW PC256	P02861	107.5; 30	LEGGDALLASQ QWLQPR AQQLAAQLPAMoxCR	1072.5; 2 826.4; 2 1472.7; 2

## Supplementary Materials

The following are available online, Figure S1: Raw data of analyzed protein spots after cold and drought treatments.
