# Supplementary material for: Proteomic Analysis of Proteins Responsive to Drought and Low Temperature Stress in a Hard Red Spring Wheat Cultivar
Source: Molecules. 2020 Mar 17;25(6):1366. doi: 10.3390/molecules25061366 (PMC7144396; doi:10.3390/molecules25061366)
Supplement: Supplementary file 1 [file molecules-25-01366-s001.pdf]

Raw data of all analyses

Statistics performed considering Cold treatment

Reference image

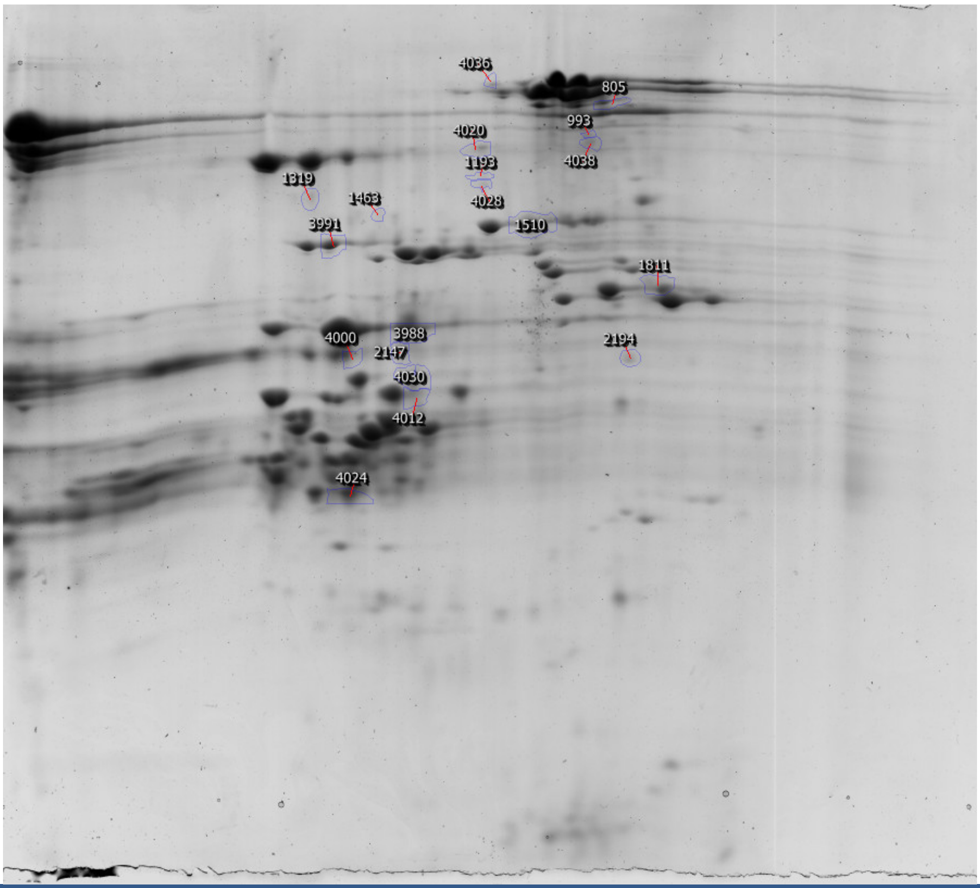

Experiment Design

Condition	Control	Cold
Replicates	9	9

Spots

#	Anova (p)	Fold	Tags	Notes	pI	MW	Protein Accession	Protein Description	Protein pI	Protein MW	Protein URL	Average Normalised Volumes	
												Control	Cold
1463	0,022	1,8										4,531e+005	8,164e+005
4030	0,047	1,8										1,112e+007	1,947e+007
4020	0,008	1,7										2,780e+006	4,730e+006
1193	0,029	1,6										5,029e+005	8,239e+005
4038	0,019	1,6										1,440e+006	2,346e+006
4028	0,031	1,6										6,241e+005	9,834e+005
3991	0,026	1,6										8,956e+006	1,392e+007
1319	0,034	1,5										1,610e+006	2,417e+006
4036	0,030	1,5										1,685e+006	2,485e+006
2194	0,034	1,5										1,292e+006	1,886e+006
4012	0,044	1,4										5,656e+006	8,185e+006
993	0,041	1,4										4,361e+005	6,207e+005
1811	0,032	1,4										9,180e+006	1,295e+007
4000	0,017	1,3										6,422e+006	8,587e+006
2147	0,033	1,3										4,230e+006	5,565e+006
4024	0,046	1,3										1,926e+007	2,516e+007
805	0,050	1,3										5,620e+006	7,256e+006
3988	0,018	1,3										2,269e+007	2,921e+007
1510	0,047	1,3										1,951e+007	2,465e+007

Tags	
	Edited
	(COLD)Anova p-value ≤ 0,05
	(COLD)Max fold change ≥ 1,2
	(DROUGHT)Anova p-value ≤ 0,05
	(DROUGHT)Max fold change ≥ 1,2
	(HEAT) Anova p-value ≤ 0,05
	(HEAT) Max fold change ≥ 1,2
	(ALL) Anova p-value ≤ 0,05

	(ALL) Max fold change ≥ 1,2

Identifier 1463

Position (928, 527)

Notes

- (ALL) Max fold change ≥ 1,2
- (COLD)Anova p-value ≤ 0,05
- (COLD)Max fold change ≥ 1,2

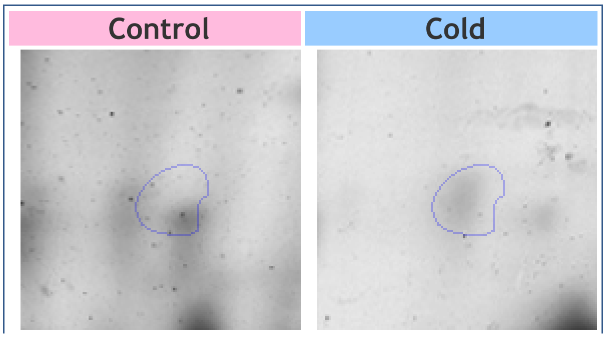

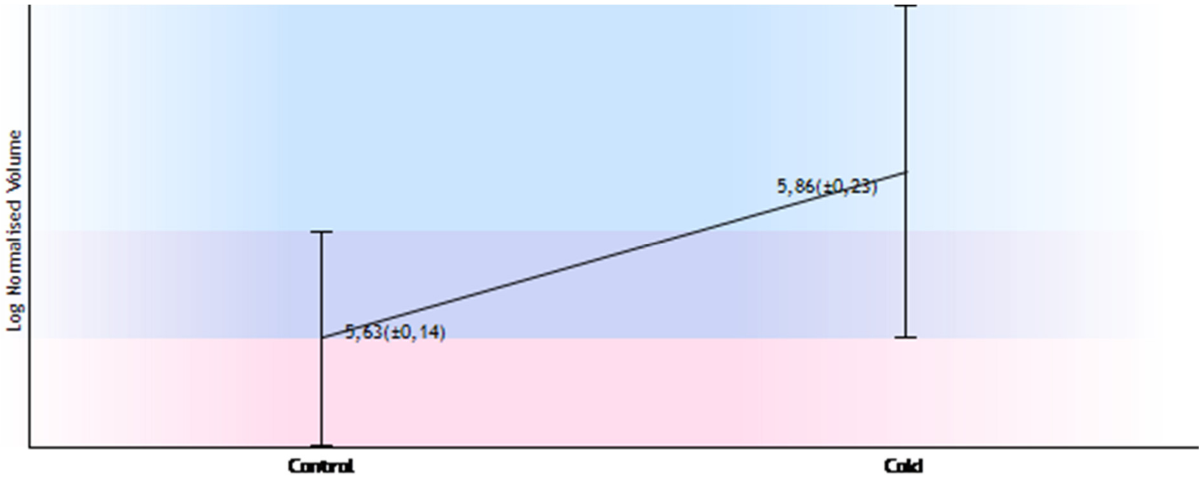

Identifier 4030

Position (1009, 914)

Notes

- (ALL) Anova p-value ≤ 0,05
- (ALL) Max fold change ≥ 1,2
- (COLD)Anova p-value ≤ 0,05
- (COLD)Max fold change ≥ 1,2
- Edited

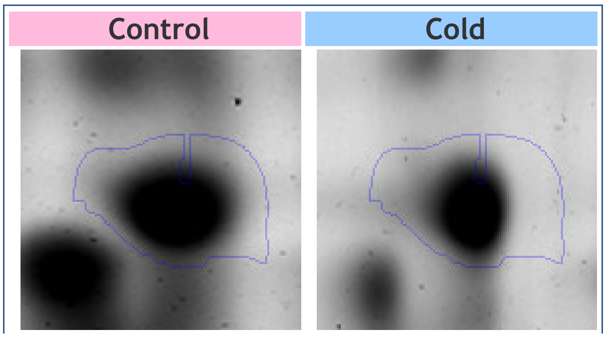

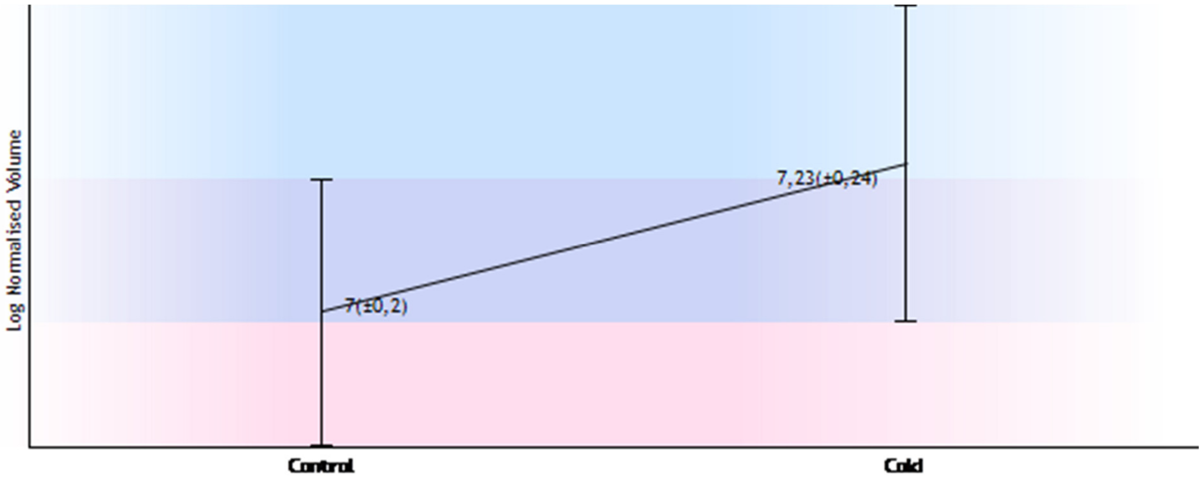

Identifier 4020

Position (1192, 357)

Notes

- (ALL) Anova p-value ≤ 0,05
- (ALL) Max fold change ≥ 1,2
- (COLD)Anova p-value ≤ 0,05
- (COLD)Max fold change ≥ 1,2
- Edited

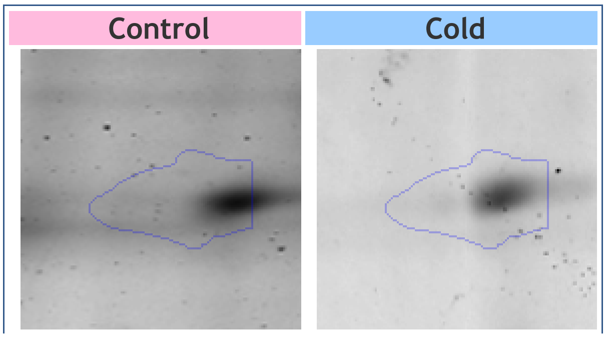

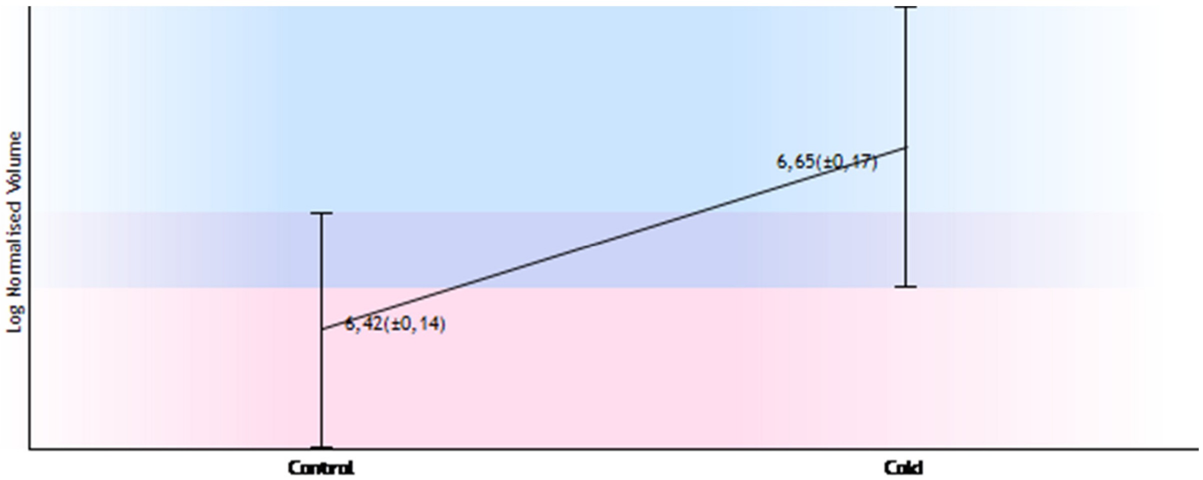

Identifier 1193

Position (1183, 419)

Notes

- (ALL) Max fold change ≥ 1,2
- (COLD)Anova p-value ≤ 0,05
- (COLD)Max fold change ≥ 1,2
- (DROUGHT) Max fold change ≥ 1,2

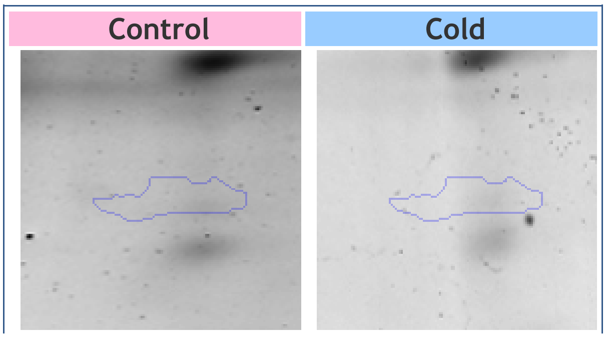

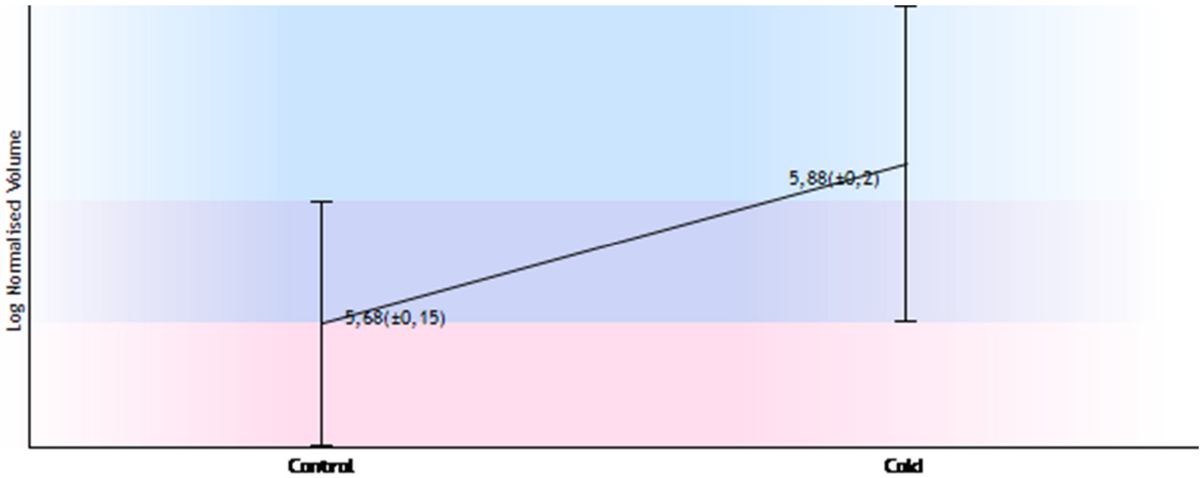

Identifier 4038

Position (1460, 349)

Notes

- (ALL) Max fold change ≥ 1,2
- (COLD)Anova p-value ≤ 0,05
- (COLD)Max fold change ≥ 1,2
- (DROUGHT)Anova p-value ≤ 0,05
- (HEAT) Max fold change ≥ 1,2
- Edited
- (DROUGHT) Max fold change ≥ 1,2

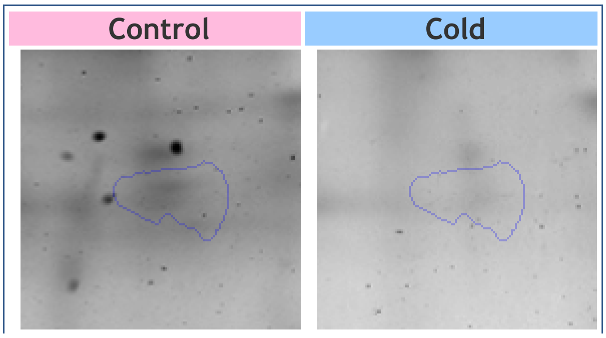

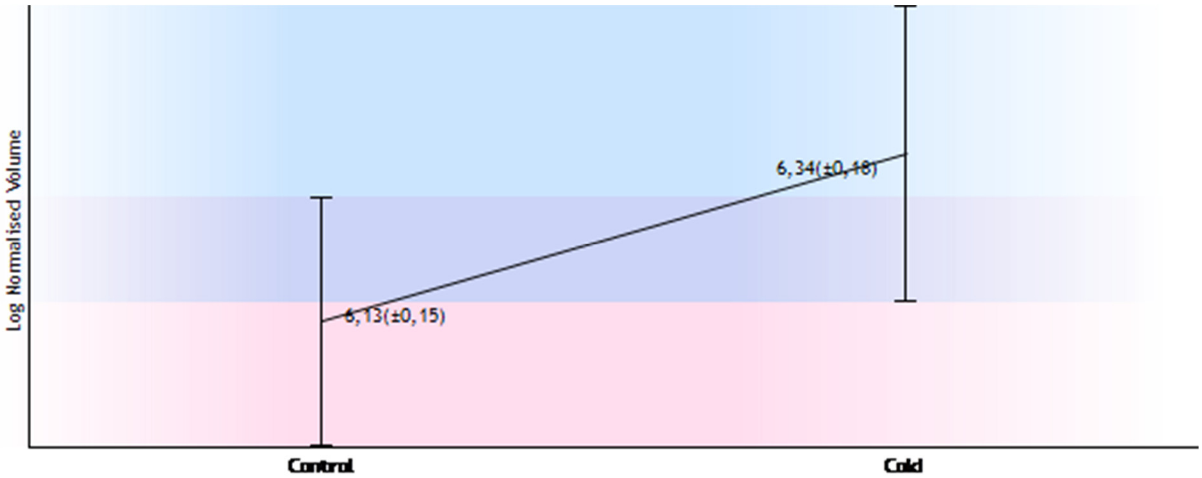

Identifier 4028

Position (1190, 438)

Notes

- (ALL) Max fold change ≥ 1,2
- (COLD)Anova p-value ≤ 0,05
- (COLD)Max fold change ≥ 1,2
- Edited
- (DROUGHT) Max fold change ≥ 1,2

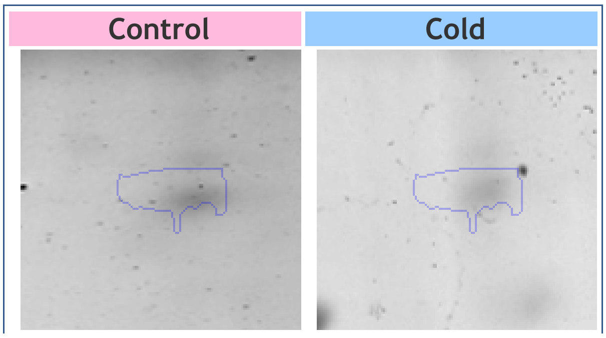

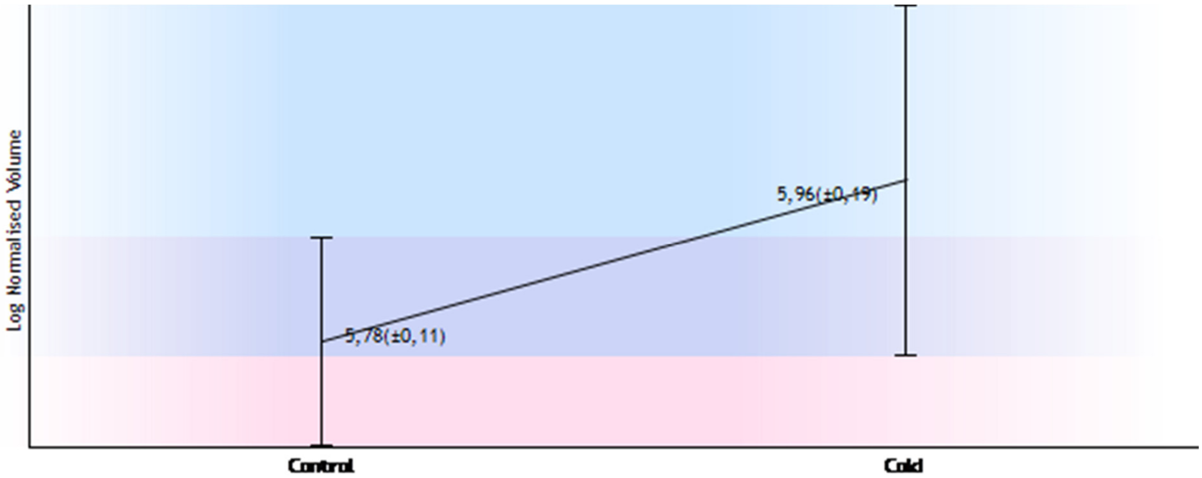

Identifier 3991

Position (803, 597)

Notes

- (ALL) Max fold change ≥ 1,2
- (COLD)Anova p-value ≤ 0,05
- (COLD)Max fold change ≥ 1,2
- Edited
- (DROUGHT)Max fold change ≥ 1,2

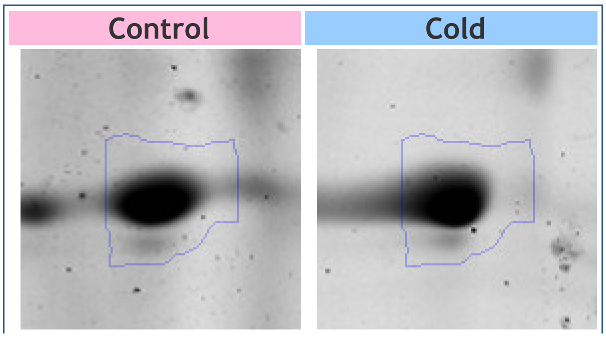

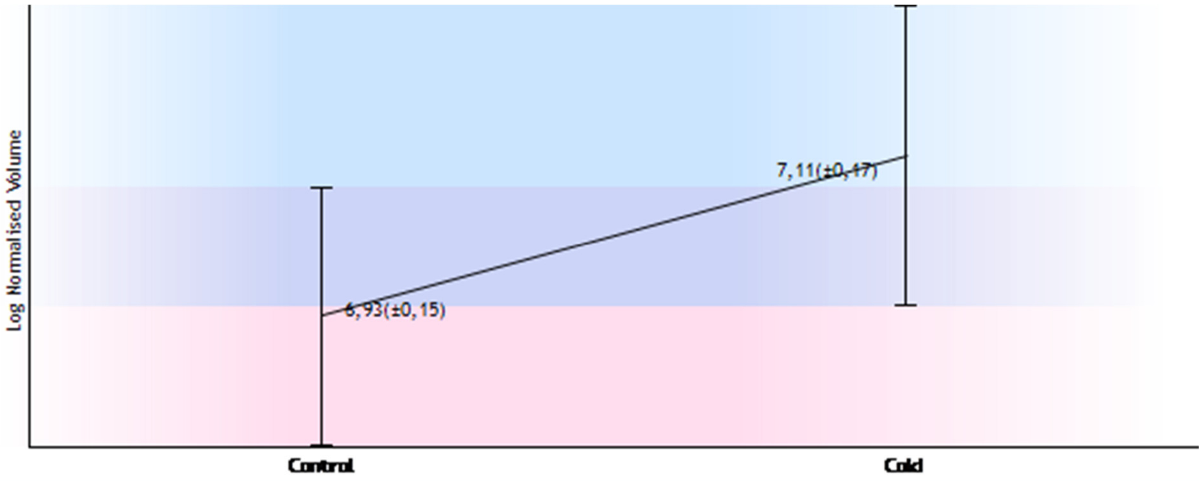

Identifier 1319

Position (753, 471)

Notes

- (ALL) Max fold change ≥ 1,2
- (COLD)Anova p-value ≤ 0,05
- (COLD)Max fold change ≥ 1,2

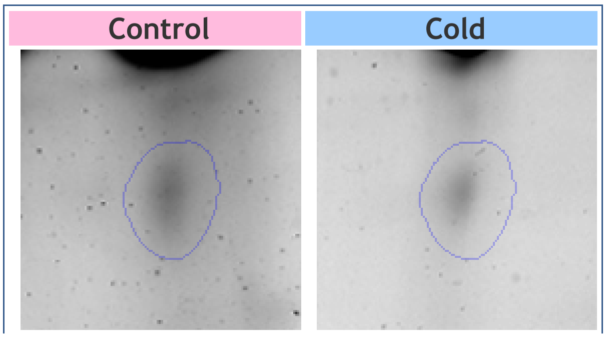

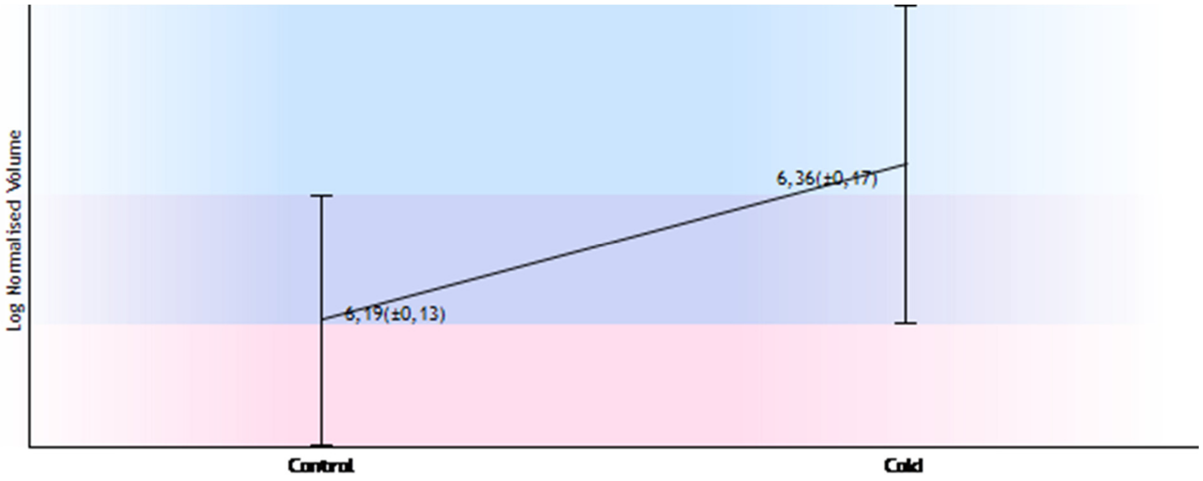

Identifier 4036

Position (1213, 194)

Notes

(ALL) Max fold change ≥ 1,2
(COLD)Anova p-value ≤ 0,05
(COLD)Max fold change ≥ 1,2
Edited
(DROUGHT) Max fold change ≥ 1,2

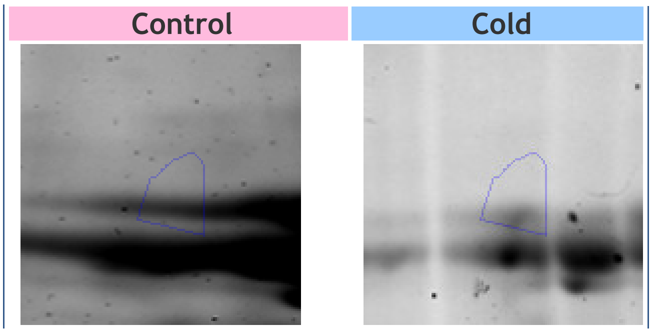

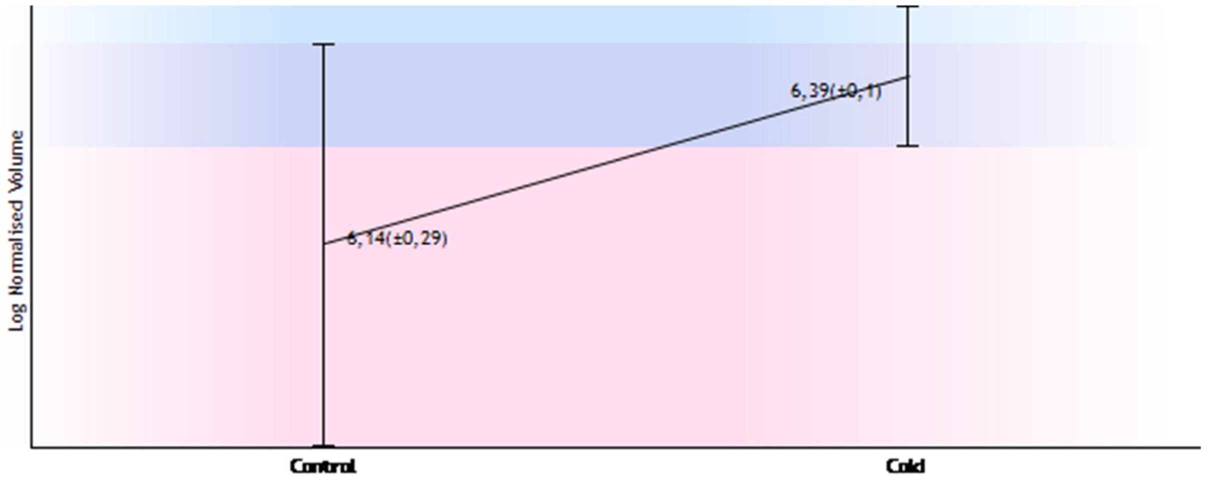

Identifier 2194

Position (1541, 874)
Notes
(ALL) Max fold change ≥ 1,2
(COLD)Anova p-value ≤ 0,05
(COLD)Max fold change ≥ 1,2
(DROUGHT) Max fold change ≥ 1,2

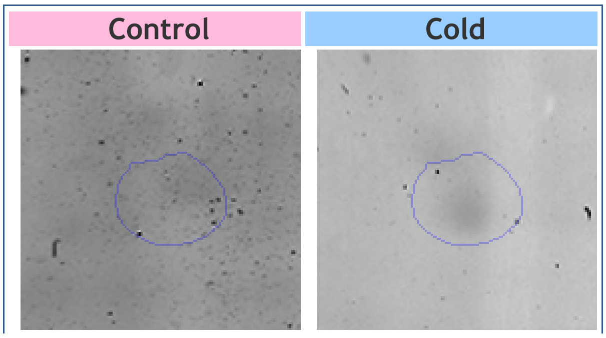

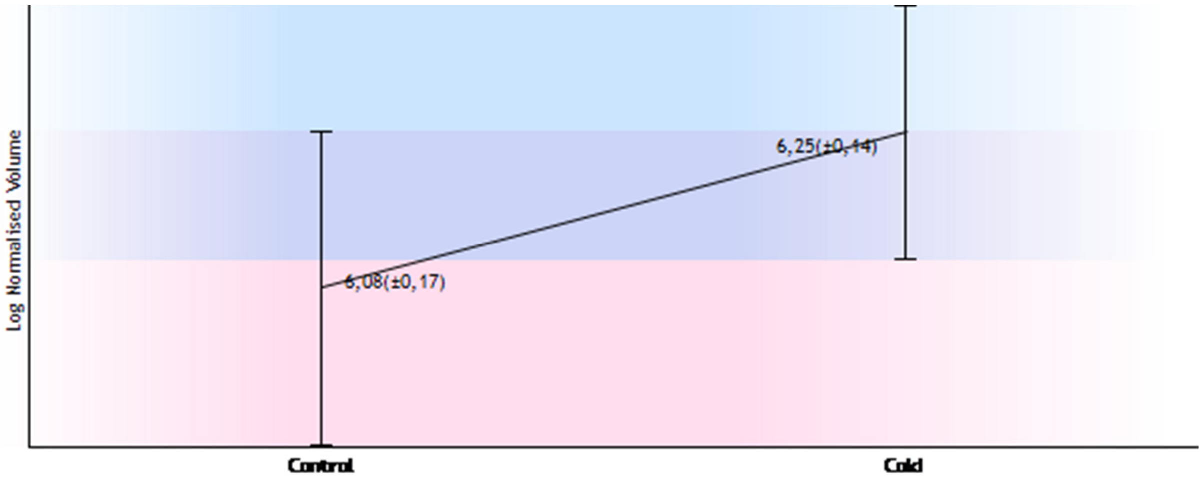

Identifier 4012

Position (1008, 967)

Notes

- (ALL) Max fold change ≥ 1,2
- (COLD)Anova p-value ≤ 0,05
- (COLD)Max fold change ≥ 1,2
- Edited
- (DROUGHT) Max fold change ≥ 1,2

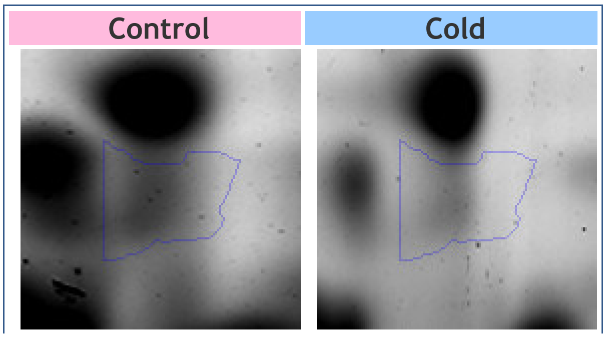

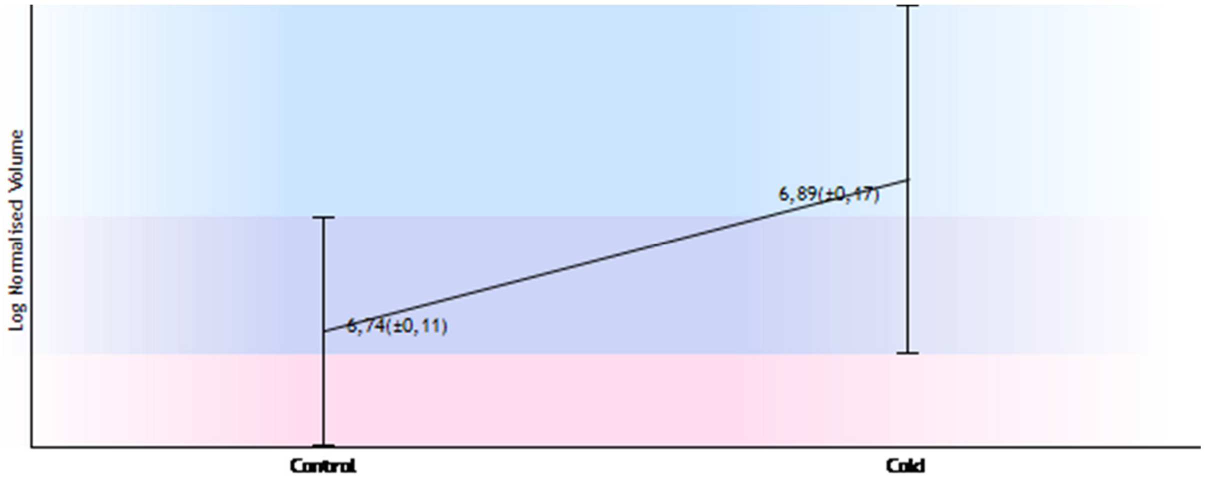

Identifier 993

Position (1439, 321)

Notes

- (ALL) Max fold change ≥ 1,2
- (COLD)Anova p-value ≤ 0,05
- (COLD)Max fold change ≥ 1,2
- (DROUGHT) Max fold change ≥ 1,2

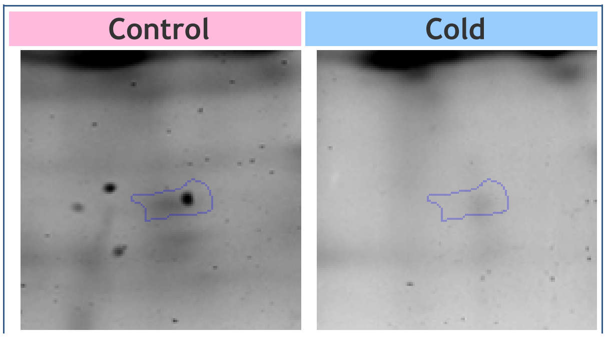

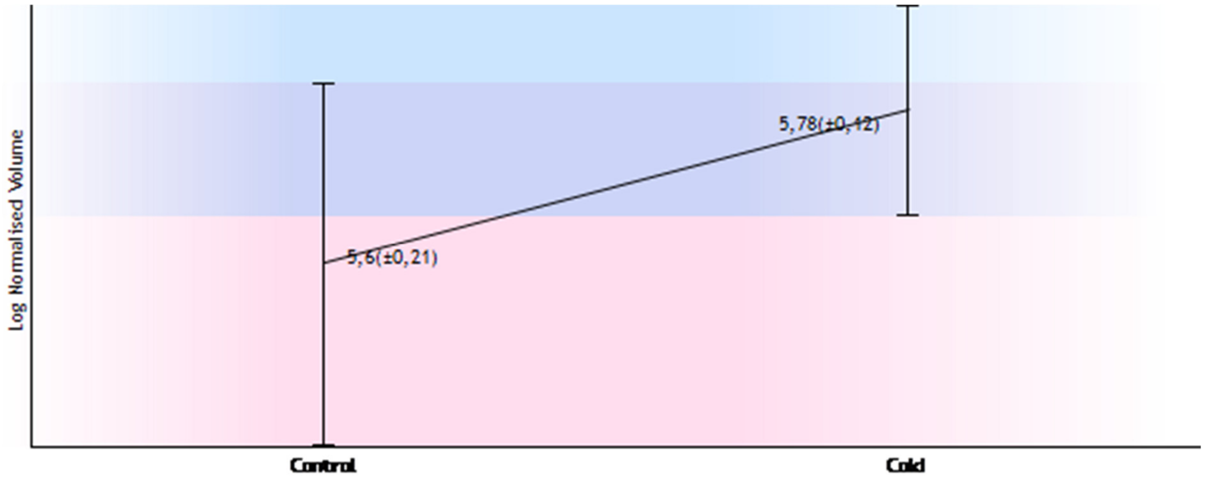

Identifier 1811

Position (1624, 707)

Notes

- (ALL) Anova p-value ≤ 0,05
- (ALL) Max fold change ≥ 1,2
- (COLD)Anova p-value ≤ 0,05
- (COLD)Max fold change ≥ 1,2
- (DROUGHT)Anova p-value ≤ 0,05
- (HEAT) Max fold change ≥ 1,2
- (DROUGHT)Max fold change ≥ 1,2

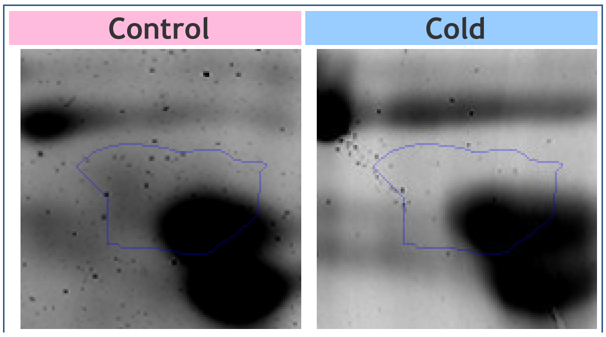

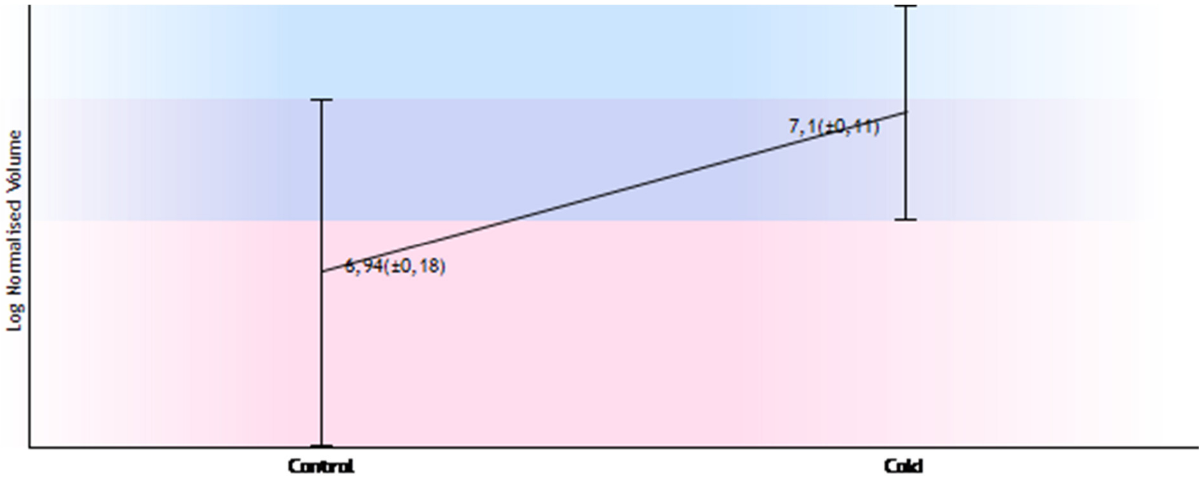

Identifier 4000

Position (845, 868)

Notes

- (ALL) Anova p-value ≤ 0,05
- (ALL) Max fold change ≥ 1,2
- (COLD)Anova p-value ≤ 0,05
- (COLD)Max fold change ≥ 1,2
- Edited

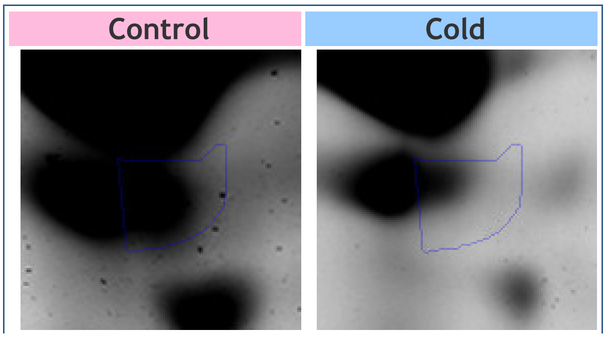

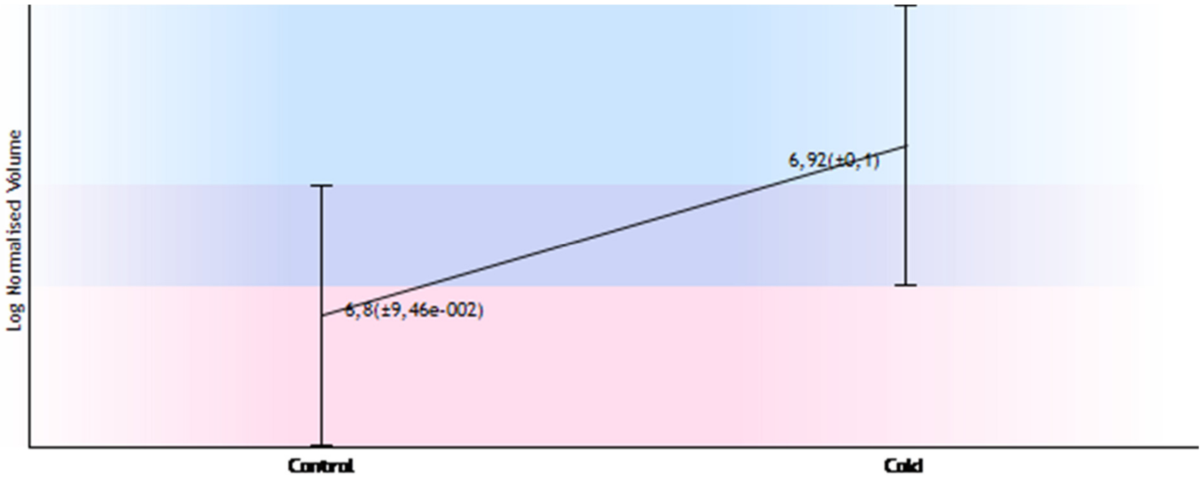

Identifier 2147

Position (986, 851)

Notes

- (ALL) Anova p-value ≤ 0,05
- (ALL) Max fold change ≥ 1,2
- (COLD)Anova p-value ≤ 0,05
- (COLD)Max fold change ≥ 1,2

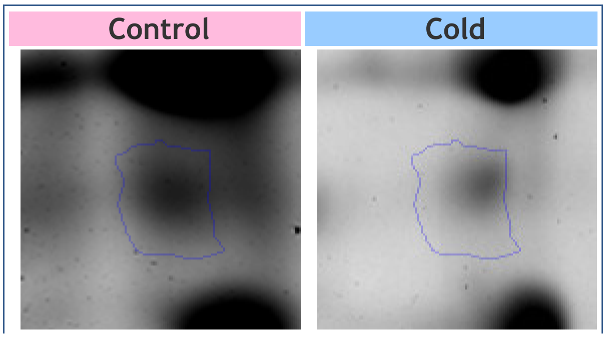

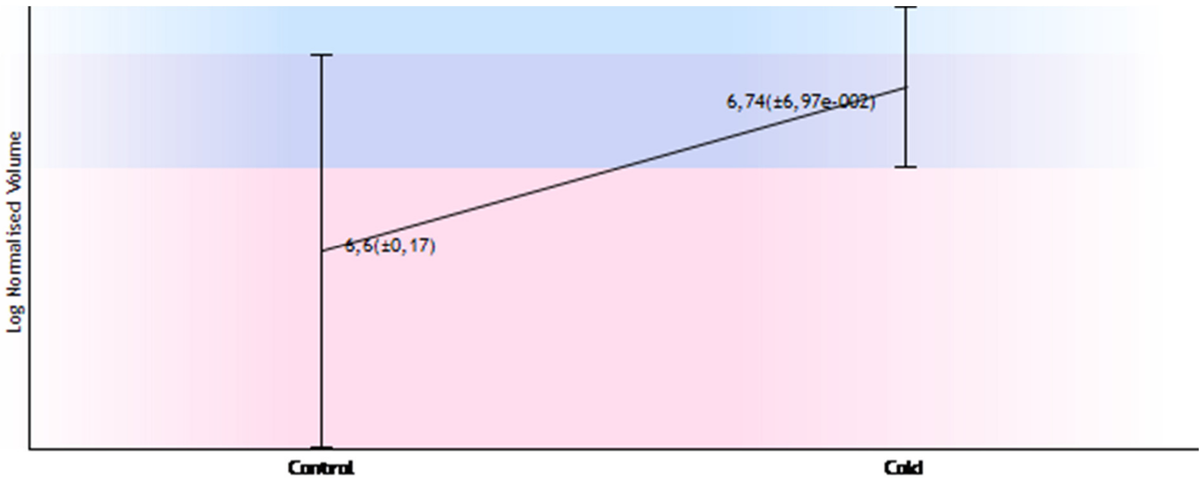

Identifier 4024

Position (822, 1194)

Notes

- (ALL) Max fold change ≥ 1,2
- (COLD)Anova p-value ≤ 0,05
- (COLD)Max fold change ≥ 1,2
- Edited

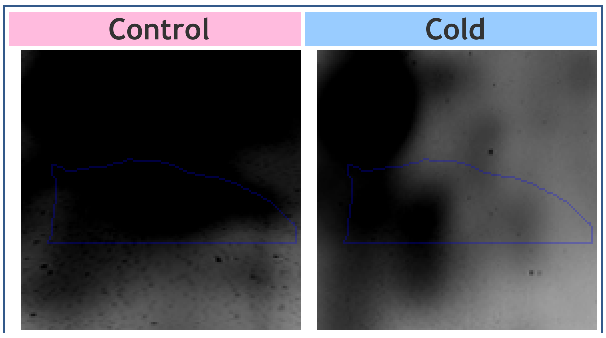

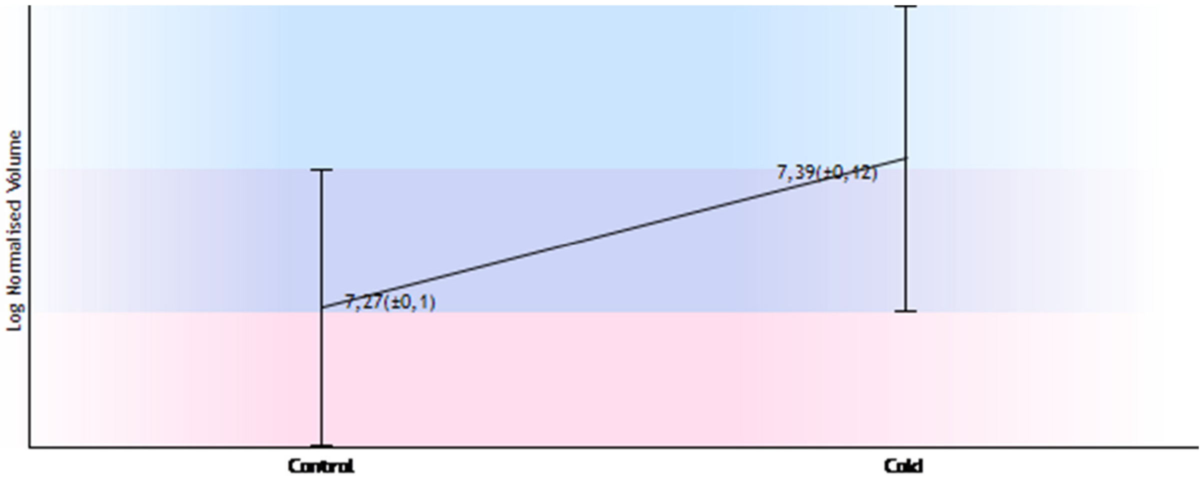

Identifier 805

Position (1476, 241)

Notes

- (ALL) Anova p-value ≤ 0,05
- (ALL) Max fold change ≥ 1,2
- (COLD)Anova p-value ≤ 0,05
- (COLD)Max fold change ≥ 1,2

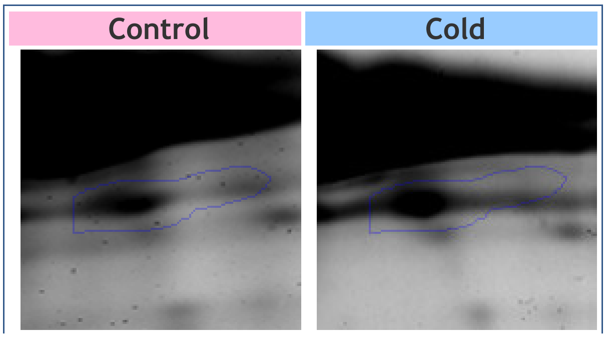

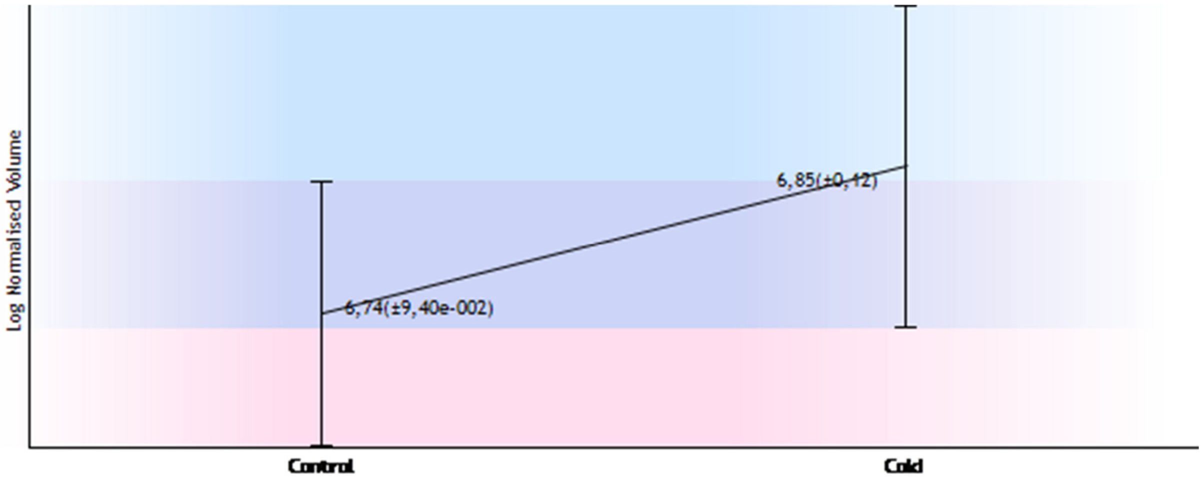

Identifier 3988

Position (985, 805)

Notes

- (ALL) Anova p-value ≤ 0,05
- (ALL) Max fold change ≥ 1,2
- (COLD)Anova p-value ≤ 0,05
- (COLD)Max fold change ≥ 1,2
- Edited

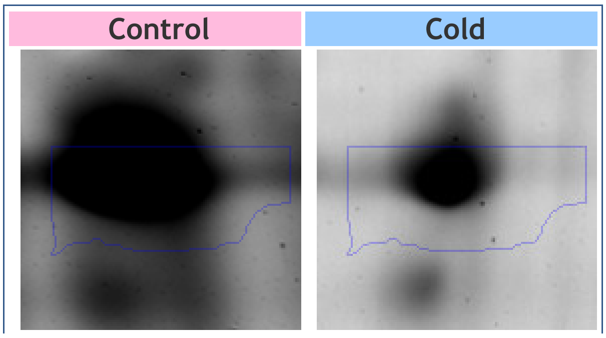

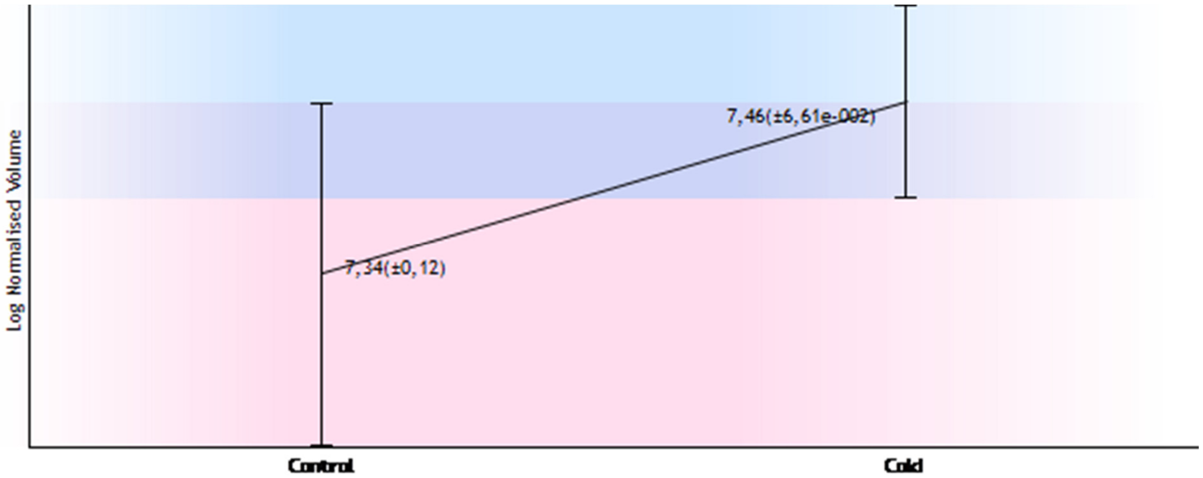

Identifier 1510

Position (1299, 551)

Notes

- (ALL) Anova p-value ≤ 0,05
- (ALL) Max fold change ≥ 1,2
- (COLD)Anova p-value ≤ 0,05
- (COLD)Max fold change ≥ 1,2
- (DROUGHT) Max fold change ≥ 1,2

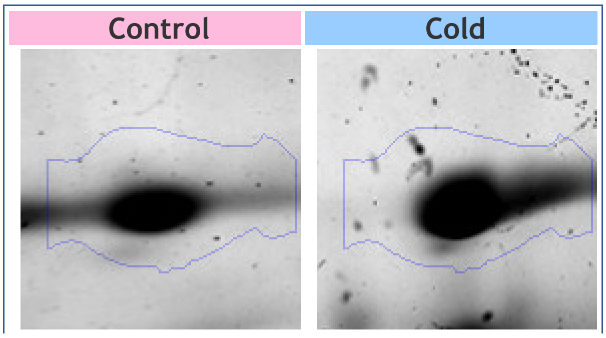

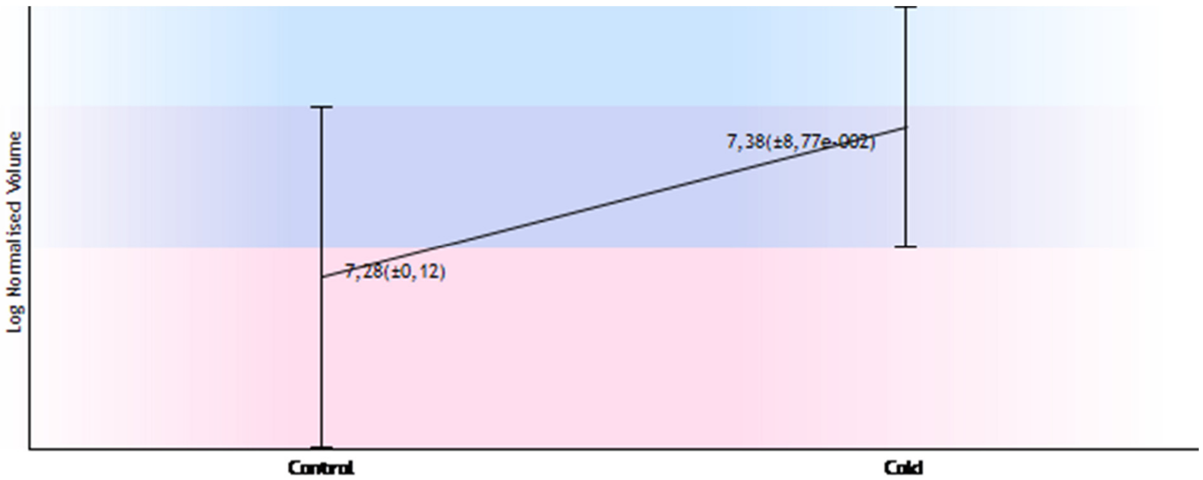

Statistics performed considering Cold/Drought/Heat treatment

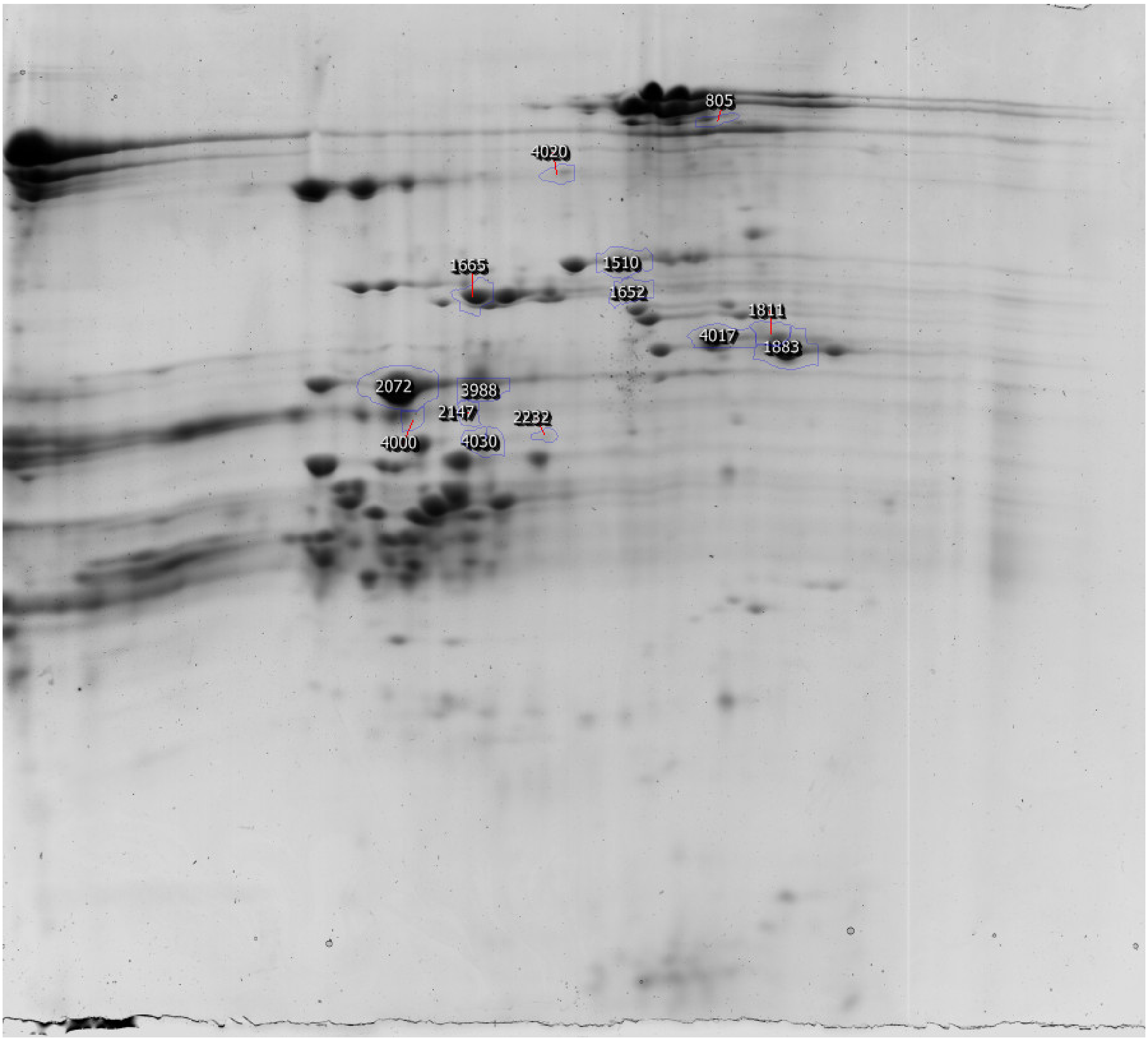

Experiment Design

Condition	Control	Cold	Drought	Heat
Replicates	9	9	10	9

Spots

#	Anova (p)	Fold	Tags	Notes	pI	MW	Protein Accession	Protein Description	Protein pI	Protein MW	Protein URL	Average Normalised Volumes			
												Control	Cold	Drought	Heat
2232	0,010	2,5										9,734e+005	1,242e+006	6,981e+005	1,725e+006
4030	0,044	1,8										1,112e+007	1,947e+007	1,182e+007	1,705e+007
1811	0,009	1,7										9,180e+006	1,295e+007	1,568e+007	1,331e+007
4020	0,026	1,7										2,780e+006	4,730e+006	3,124e+006	3,087e+006
1652	0,048	1,6										7,843e+006	9,593e+006	1,090e+007	1,262e+007
1883	0,008	1,6										3,578e+007	4,081e+007	5,607e+007	3,694e+007
805	0,011	1,5										5,620e+006	7,256e+006	5,867e+006	4,719e+006
1510	0,042	1,5										1,951e+007	2,465e+007	2,534e+007	2,862e+007
2147	0,035	1,4										4,230e+006	5,565e+006	4,003e+006	3,852e+006
2072	0,011	1,4										1,049e+008	1,254e+008	1,074e+008	8,757e+007
4017	0,043	1,4										2,736e+007	2,804e+007	3,813e+007	2,826e+007
4000	0,032	1,4										6,422e+006	8,587e+006	6,366e+006	6,216e+006
1665	0,049	1,4										2,687e+007	3,474e+007	3,572e+007	2,598e+007
3988	0,018	1,4										2,269e+007	2,921e+007	2,291e+007	2,157e+007

Tags	
	Edited
	(COLD)Anova p-value ≤ 0,05
	(COLD)Max fold change ≥ 1,2
	(DROUGHT)Anova p-value ≤ 0,05
	(DROUGHT) Max fold change ≥ 1,2
	(HEAT) Anova p-value ≤ 0,05
	(HEAT) Max fold change ≥ 1,2
	(ALL) Anova p-value ≤ 0,05
	(ALL) Max fold change ≥ 1,2

Identifier 2232

Position (1140, 906)

Notes

- (ALL) Anova p-value ≤ 0,05
- (ALL) Max fold change ≥ 1,2
- (COLD)Max fold change ≥ 1,2
- (DROUGHT) Max fold change ≥ 1,2

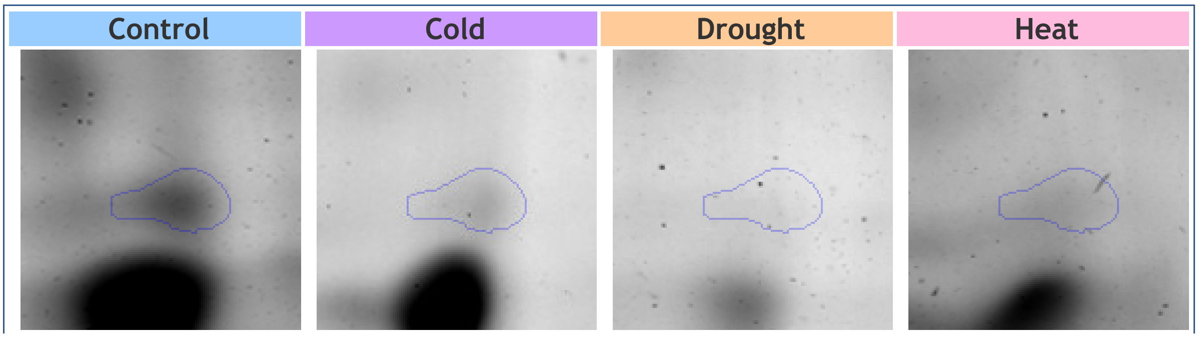

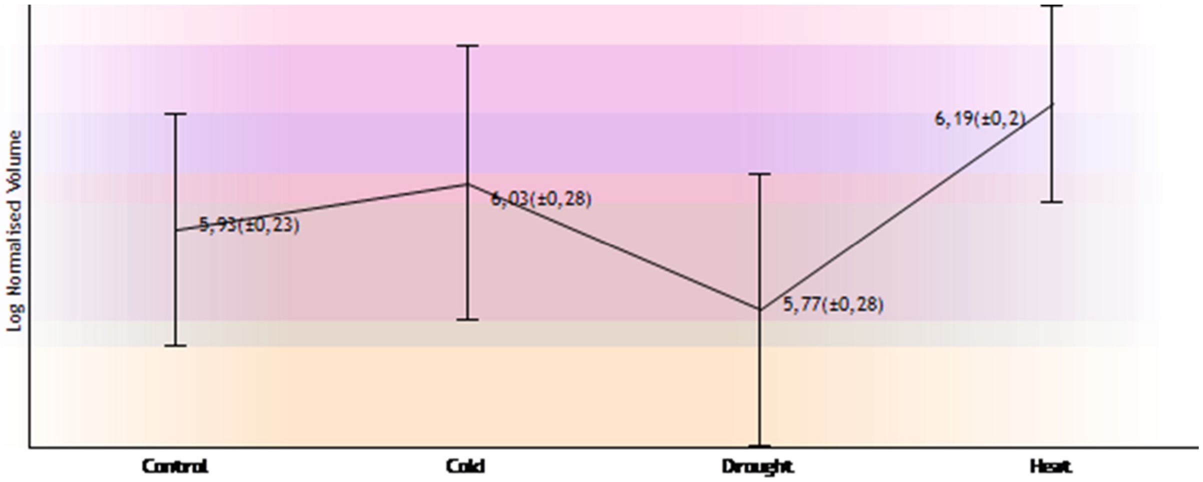

Identifier 4030

Position (1009, 914)

Notes

- (ALL) Anova p-value ≤ 0,05
- (ALL) Max fold change ≥ 1,2
- (COLD)Anova p-value ≤ 0,05
- (COLD)Max fold change ≥ 1,2
- Edited

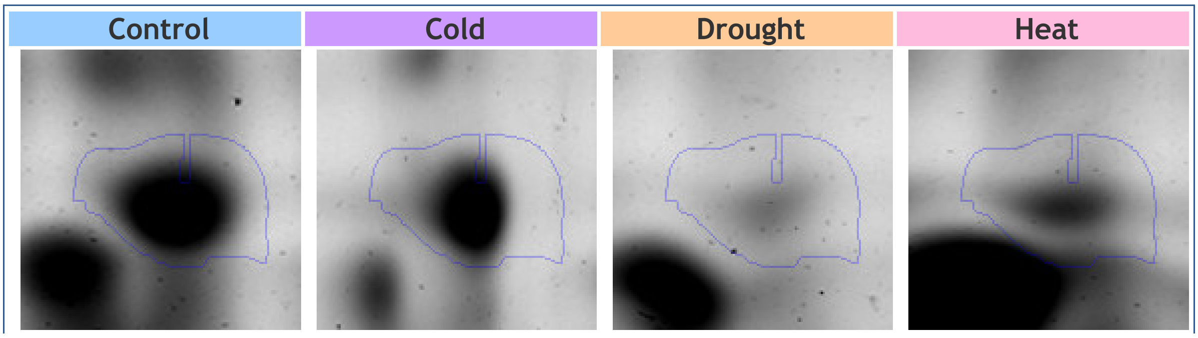

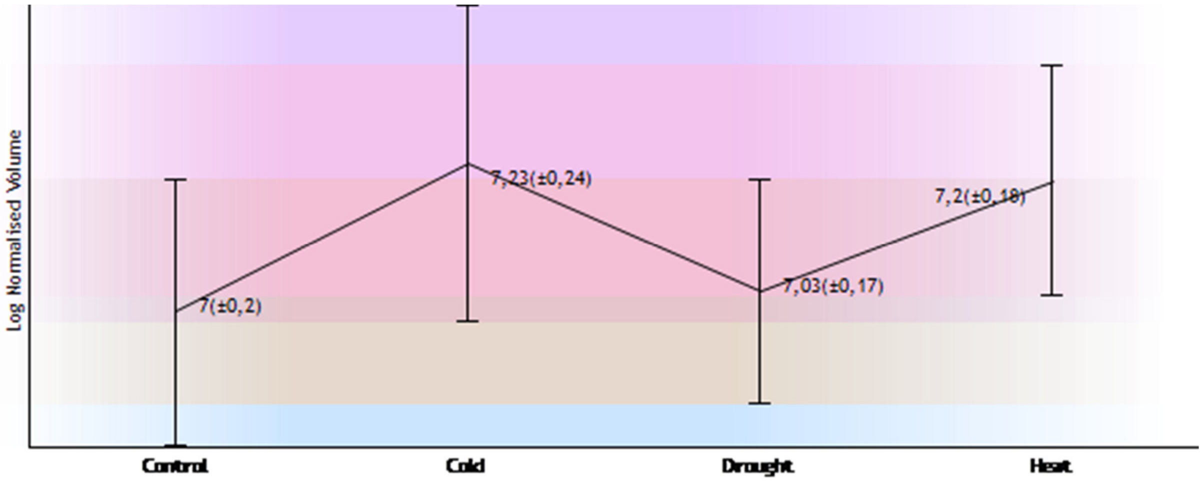

Identifier 1811

Position (1624, 707)

Notes

- (ALL) Anova p-value ≤ 0,05
- (ALL) Max fold change ≥ 1,2
- (COLD)Anova p-value ≤ 0,05
- (COLD)Max fold change ≥ 1,2
- (DROUGHT)Anova p-value ≤ 0,05
- (HEAT) Max fold change ≥ 1,2
- (DROUGHT) Max fold change ≥ 1,2

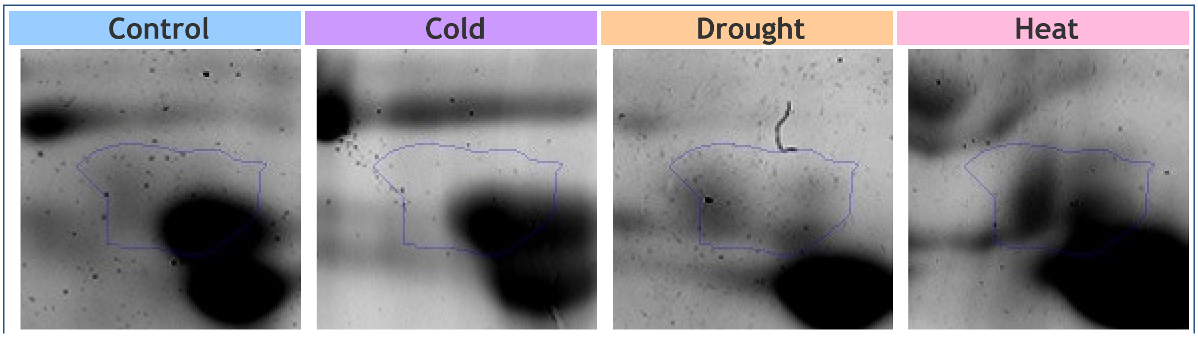

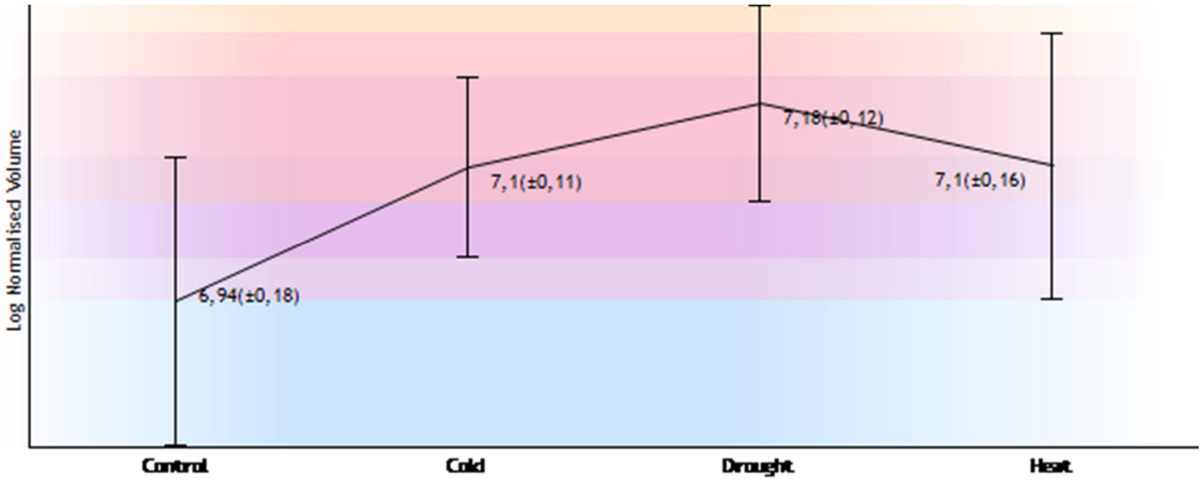

Identifier 4020

Position (1192, 357)

Notes

- (ALL) Anova p-value ≤ 0,05
- (ALL) Max fold change ≥ 1,2
- (COLD)Anova p-value ≤ 0,05
- (COLD)Max fold change ≥ 1,2
- Edited

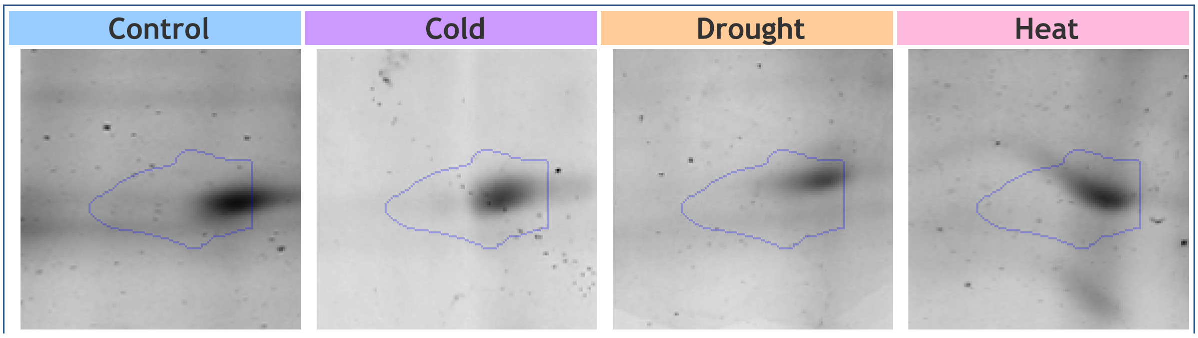

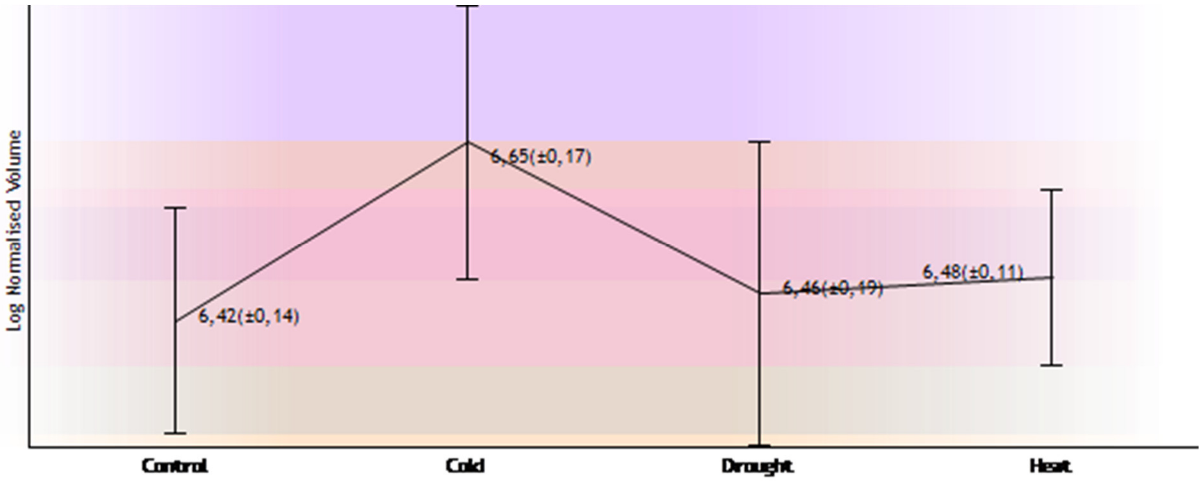

Identifier 1652

Position (1308, 612)

Notes

- (ALL) Anova p-value ≤ 0,05
- (ALL) Max fold change ≥ 1,2
- (COLD)Max fold change ≥ 1,2
- (DROUGHT) Max fold change ≥ 1,2

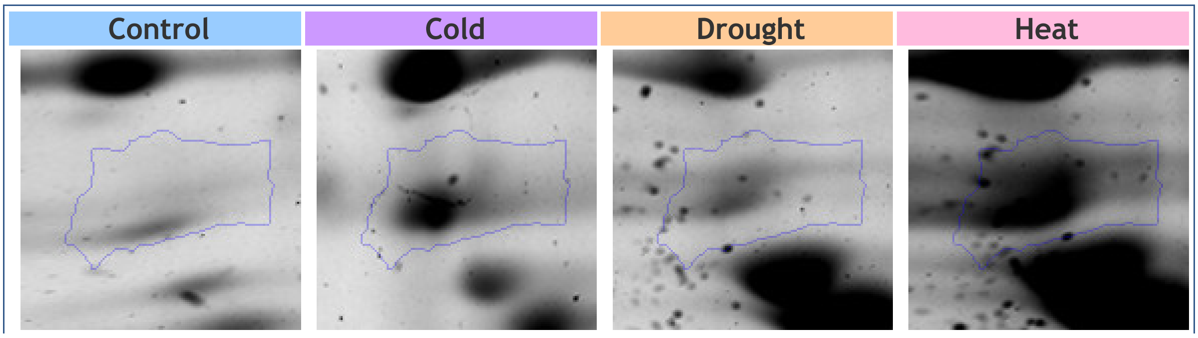

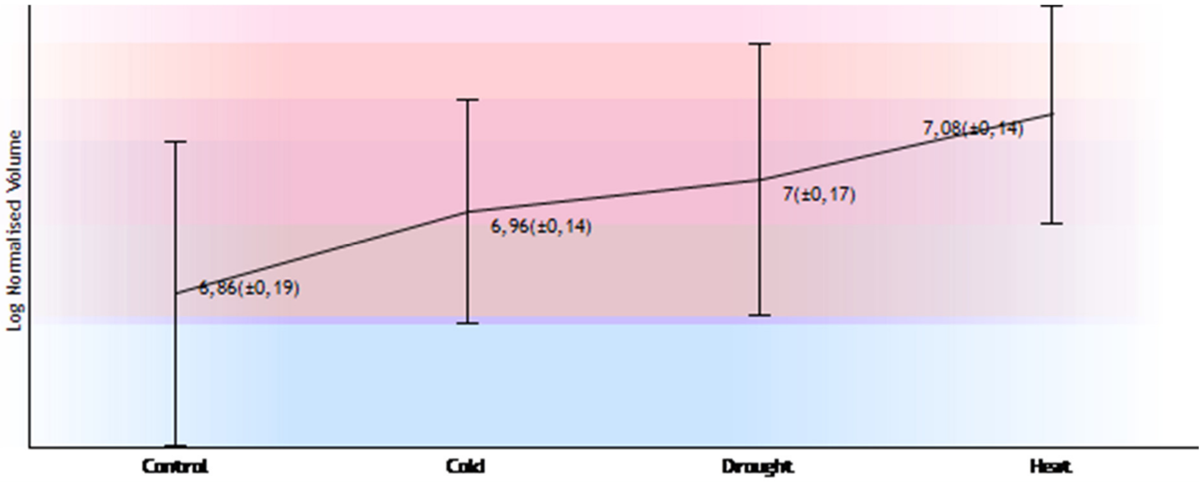

Identifier 1883

Position (1643, 740)

Notes

- (ALL) Anova p-value ≤ 0,05
- (ALL) Max fold change ≥ 1,2
- (DROUGHT)Anova p-value ≤ 0,05
- (DROUGHT) Max fold change ≥ 1,2

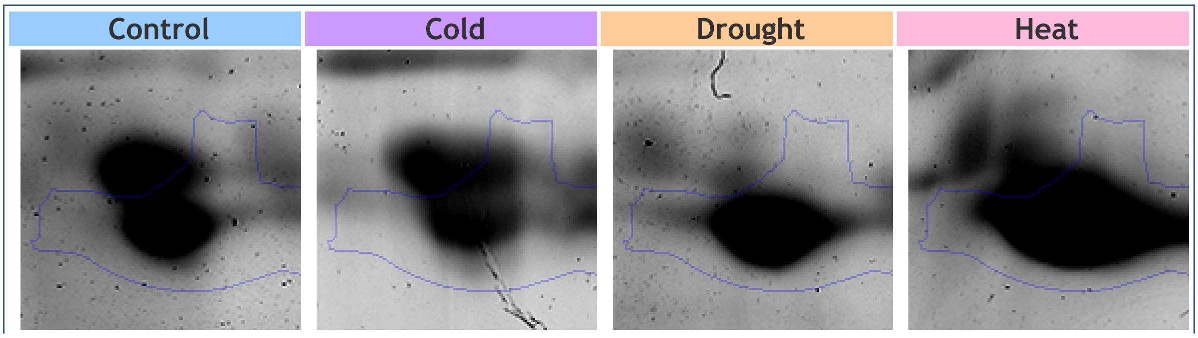

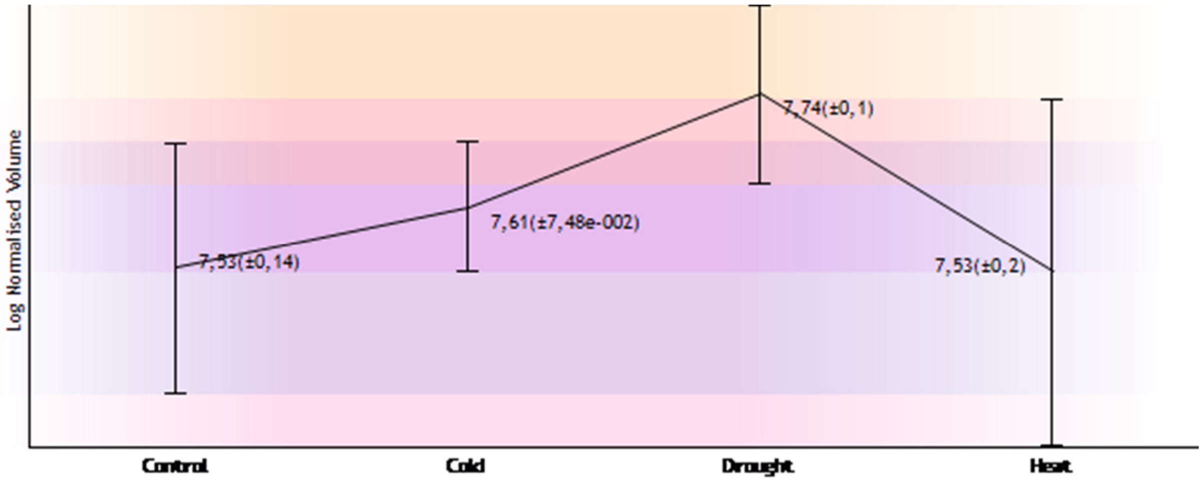

Identifier 805

Position (1476, 241)

Notes

- (ALL) Anova p-value ≤ 0,05
- (ALL) Max fold change ≥ 1,2
- (COLD)Anova p-value ≤ 0,05
- (COLD)Max fold change ≥ 1,2

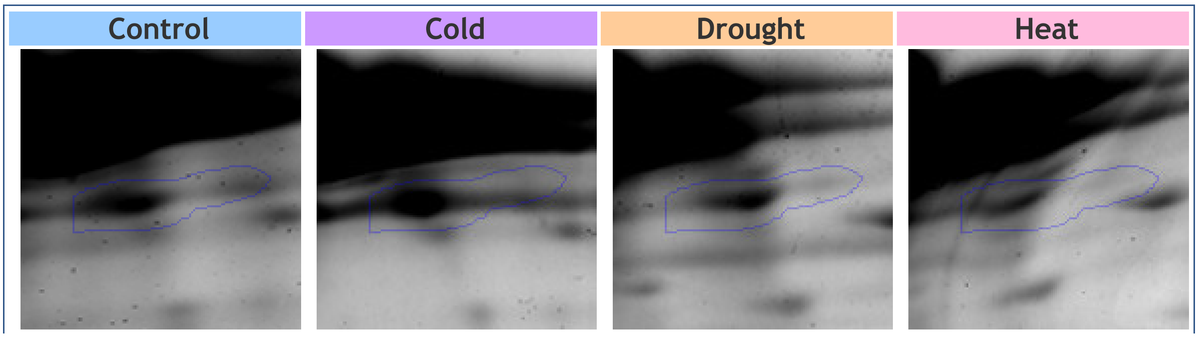

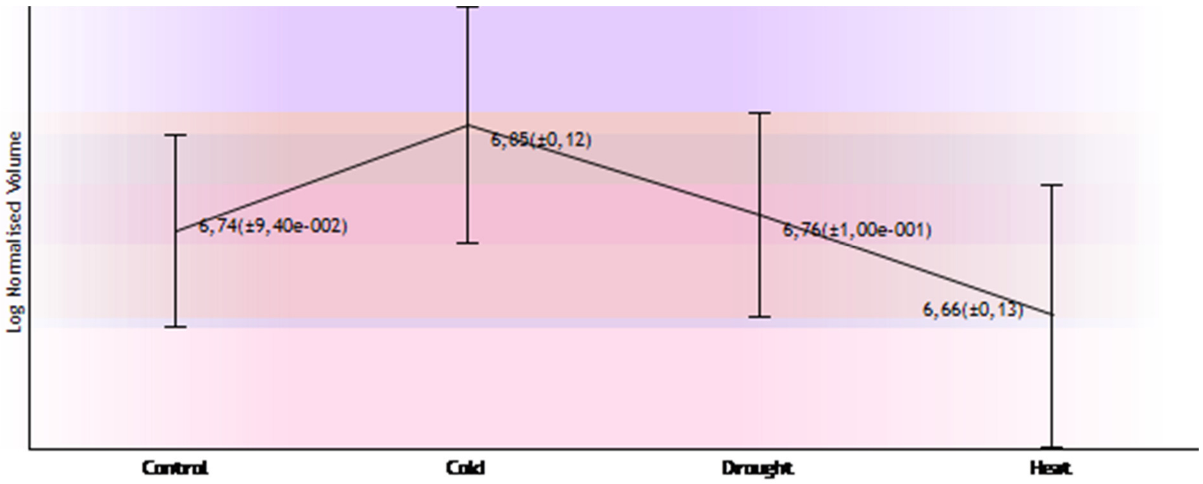

Identifier 1510

Position (1299, 551)

Notes

- (ALL) Anova p-value ≤ 0,05
- (ALL) Max fold change ≥ 1,2
- (COLD)Anova p-value ≤ 0,05
- (COLD)Max fold change ≥ 1,2
- (DROUGHT) Max fold change ≥ 1,2

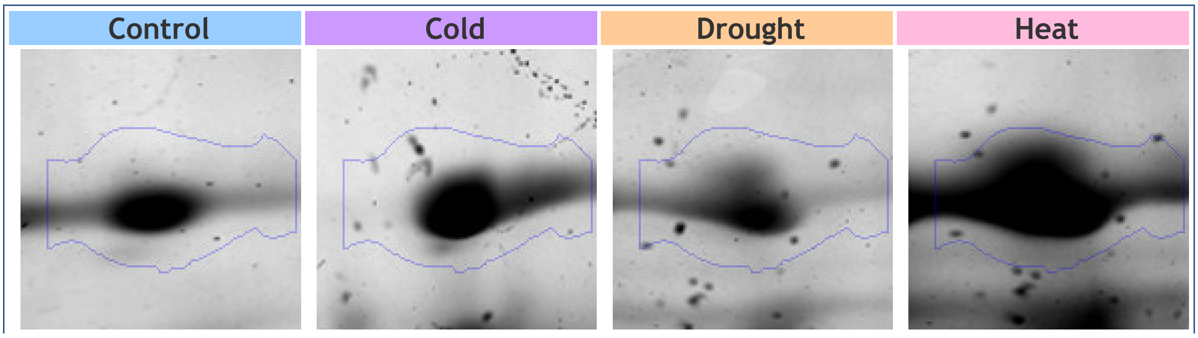

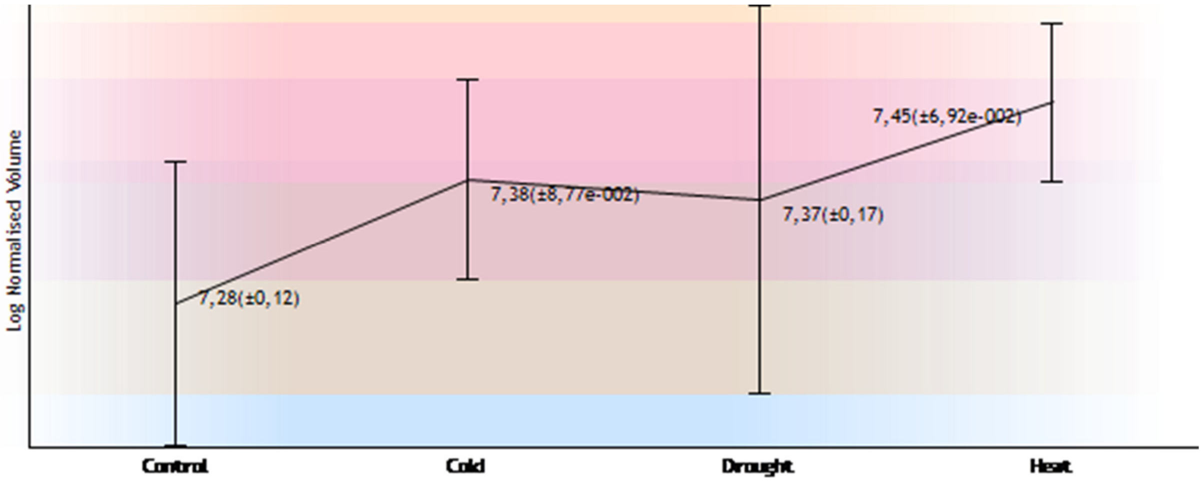

Identifier 2147

Position (986, 851)

Notes

- (ALL) Anova p-value ≤ 0,05
- (ALL) Max fold change ≥ 1,2
- (COLD)Anova p-value ≤ 0,05
- (COLD)Max fold change ≥ 1,2

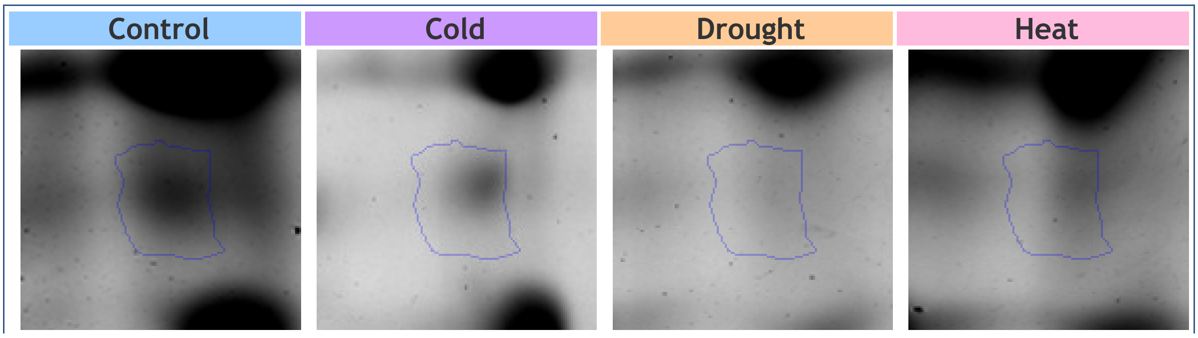

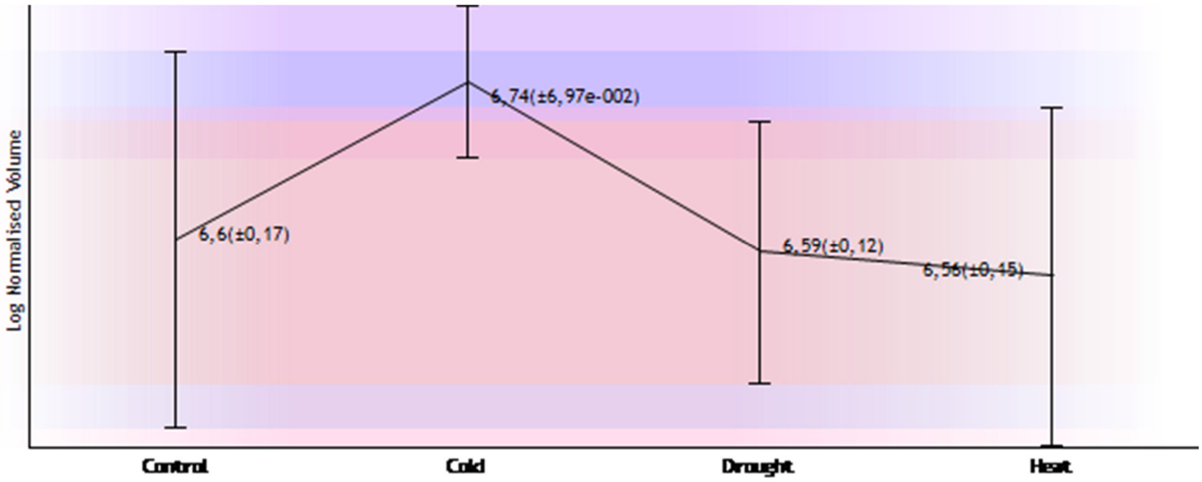

Identifier 2072

Position (828, 826)

Notes

- (ALL) Anova p-value ≤ 0,05
- (ALL) Max fold change ≥ 1,2
- (COLD)Anova p-value ≤ 0,05

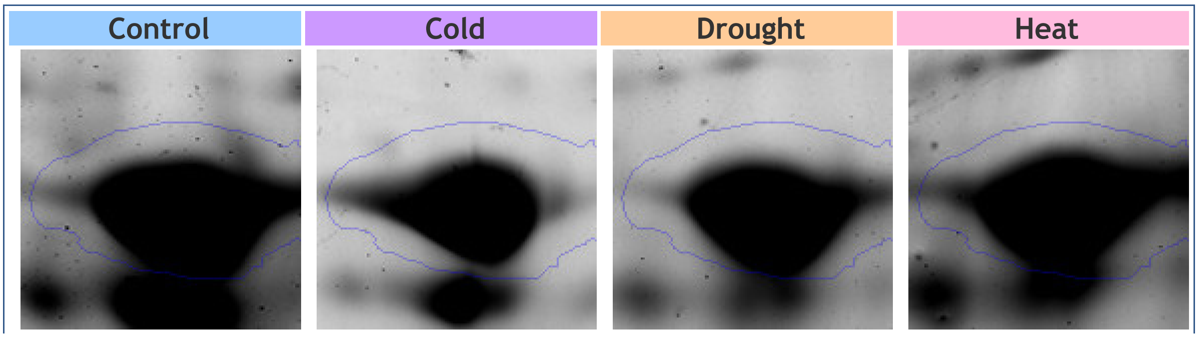

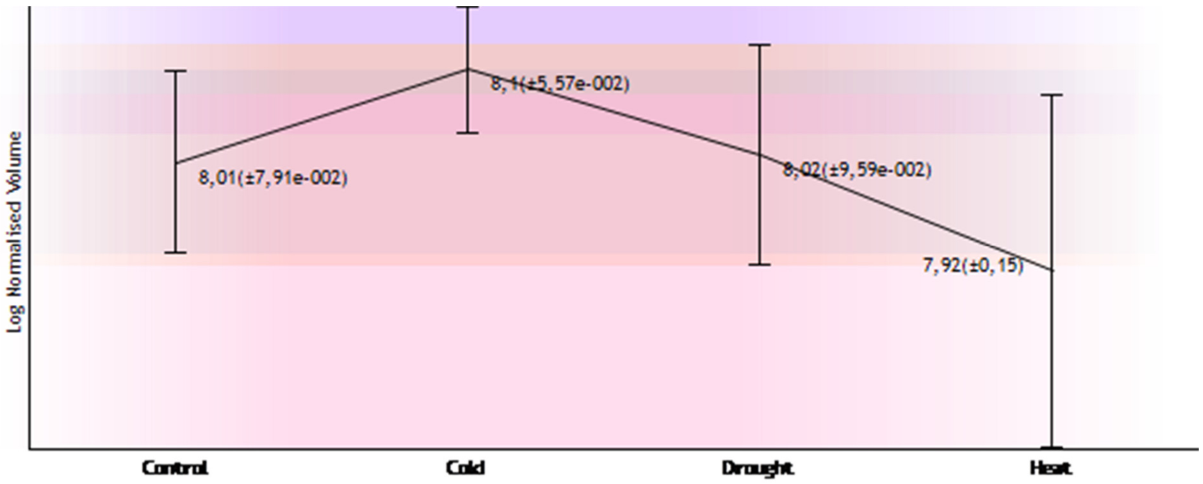

Identifier 4017

Position (1498, 702)

Notes

- (ALL) Anova p-value ≤ 0,05
- (ALL) Max fold change ≥ 1,2
- (DROUGHT)Anova p-value ≤ 0,05
- Edited
- (DROUGHT) Max fold change ≥ 1,2

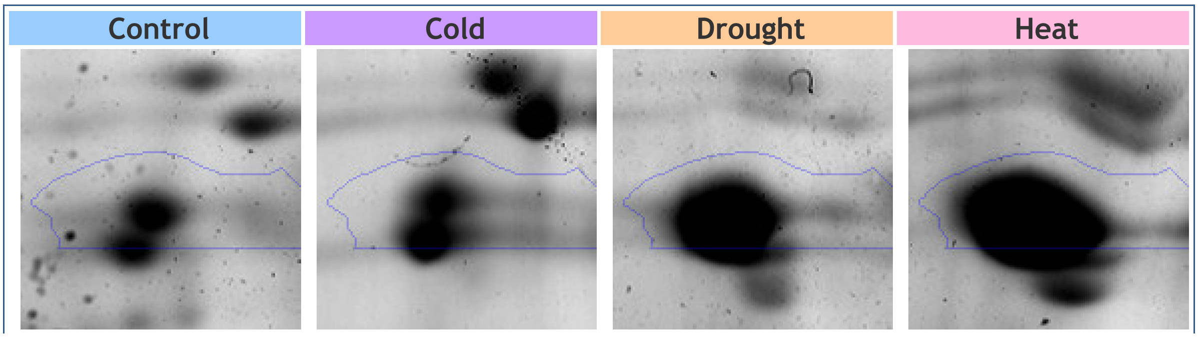

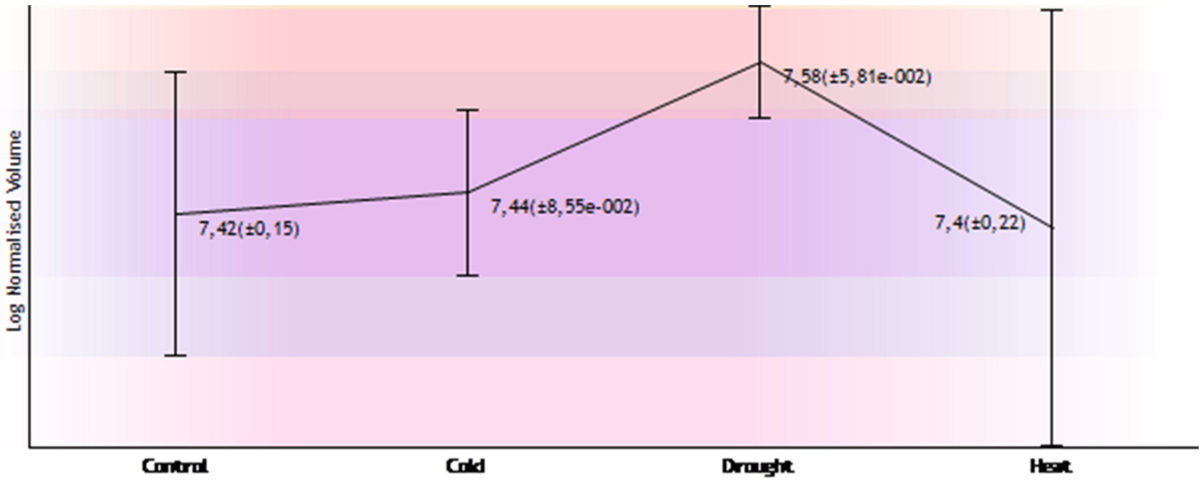

Identifier 4000

Position (845, 868)

Notes

- (ALL) Anova p-value ≤ 0,05
- (ALL) Max fold change ≥ 1,2
- (COLD)Anova p-value ≤ 0,05
- (COLD)Max fold change ≥ 1,2
- Edited

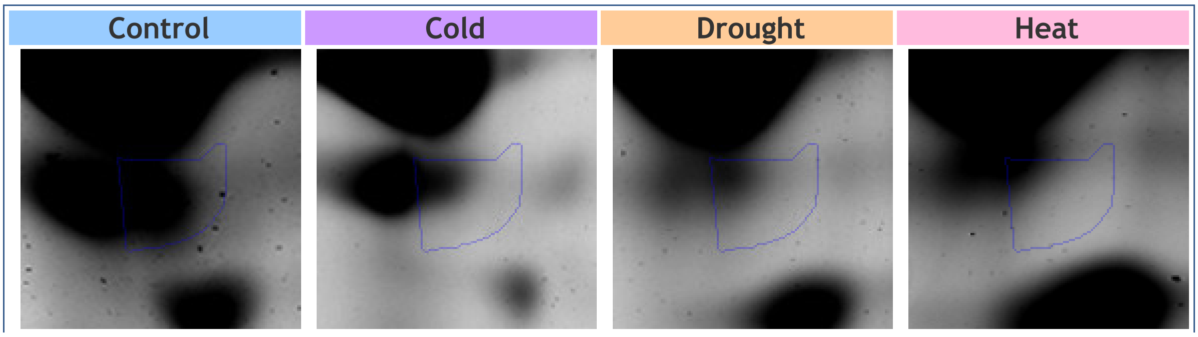

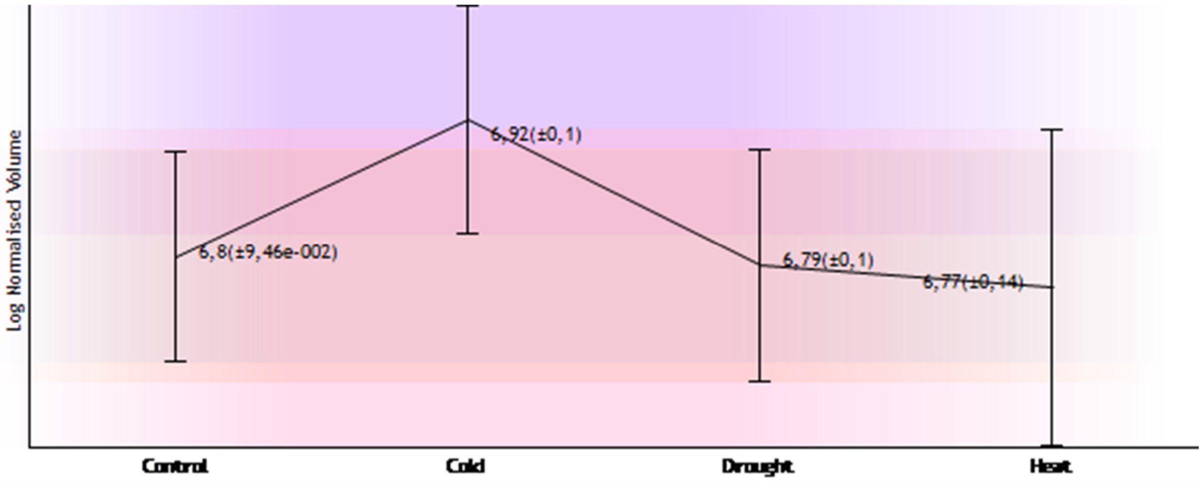

Identifier 1665

Position (994, 622)

Notes

- (ALL) Anova p-value ≤ 0,05
- (ALL) Max fold change ≥ 1,2
- (COLD)Max fold change ≥ 1,2
- (DROUGHT)Anova p-value ≤ 0,05
- (DROUGHT) Max fold change ≥ 1,2

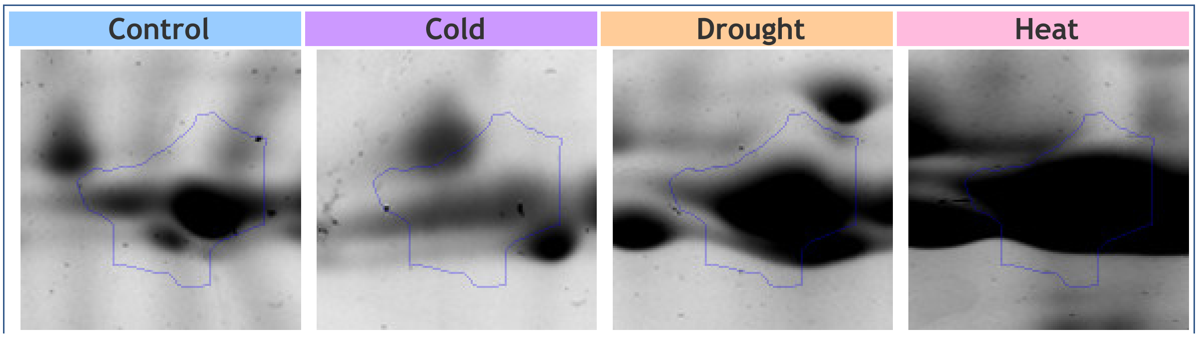

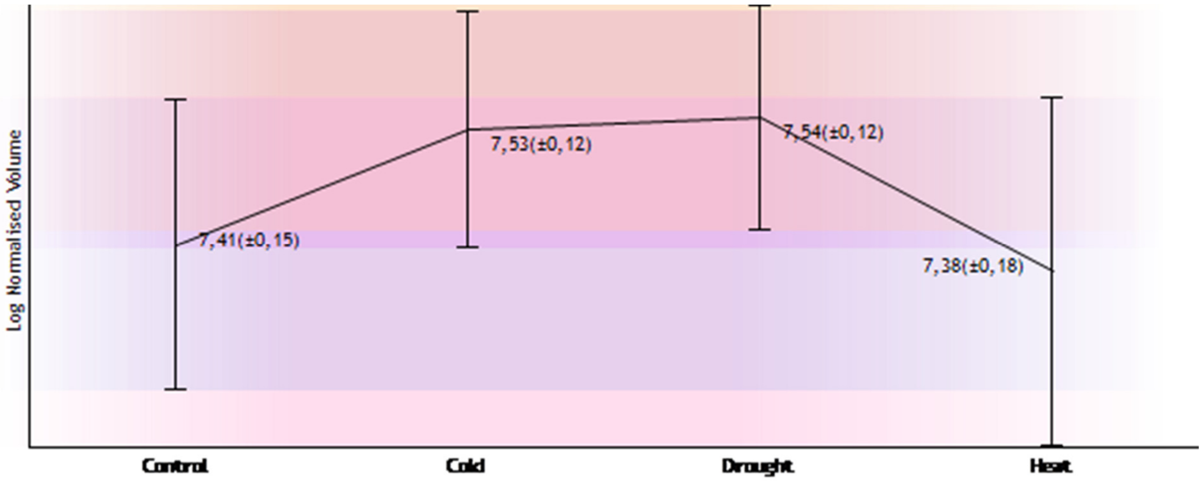

Identifier 3988

Position (985, 805)

Notes

- (ALL) Anova p-value ≤ 0,05
- (ALL) Max fold change ≥ 1,2
- (COLD)Anova p-value ≤ 0,05
- (COLD)Max fold change ≥ 1,2
- Edited

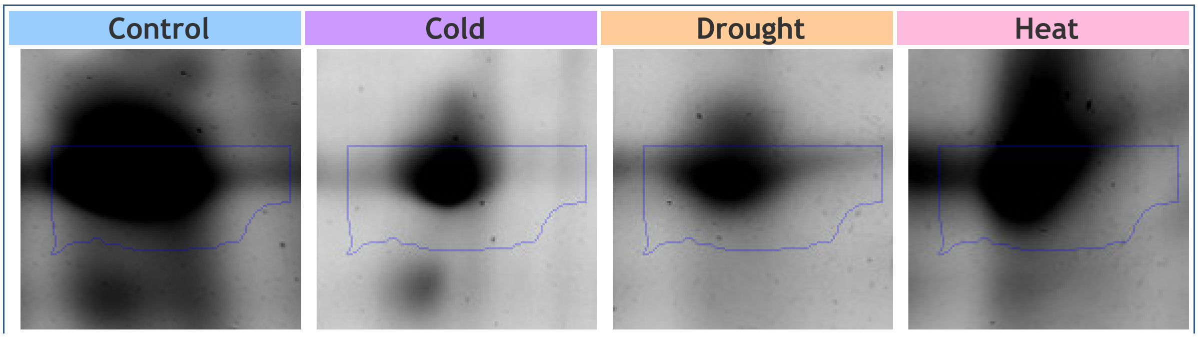

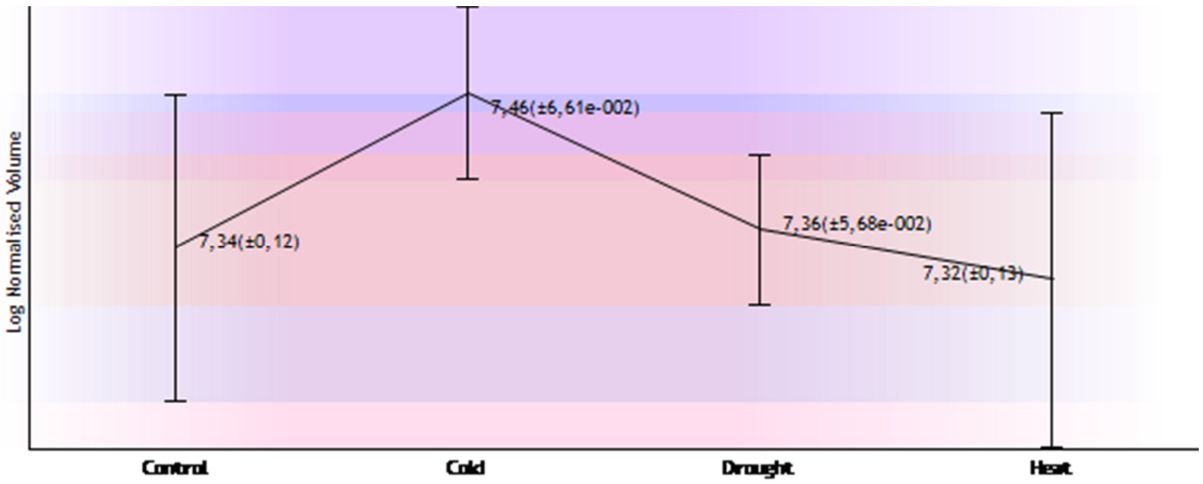

Drought treatment

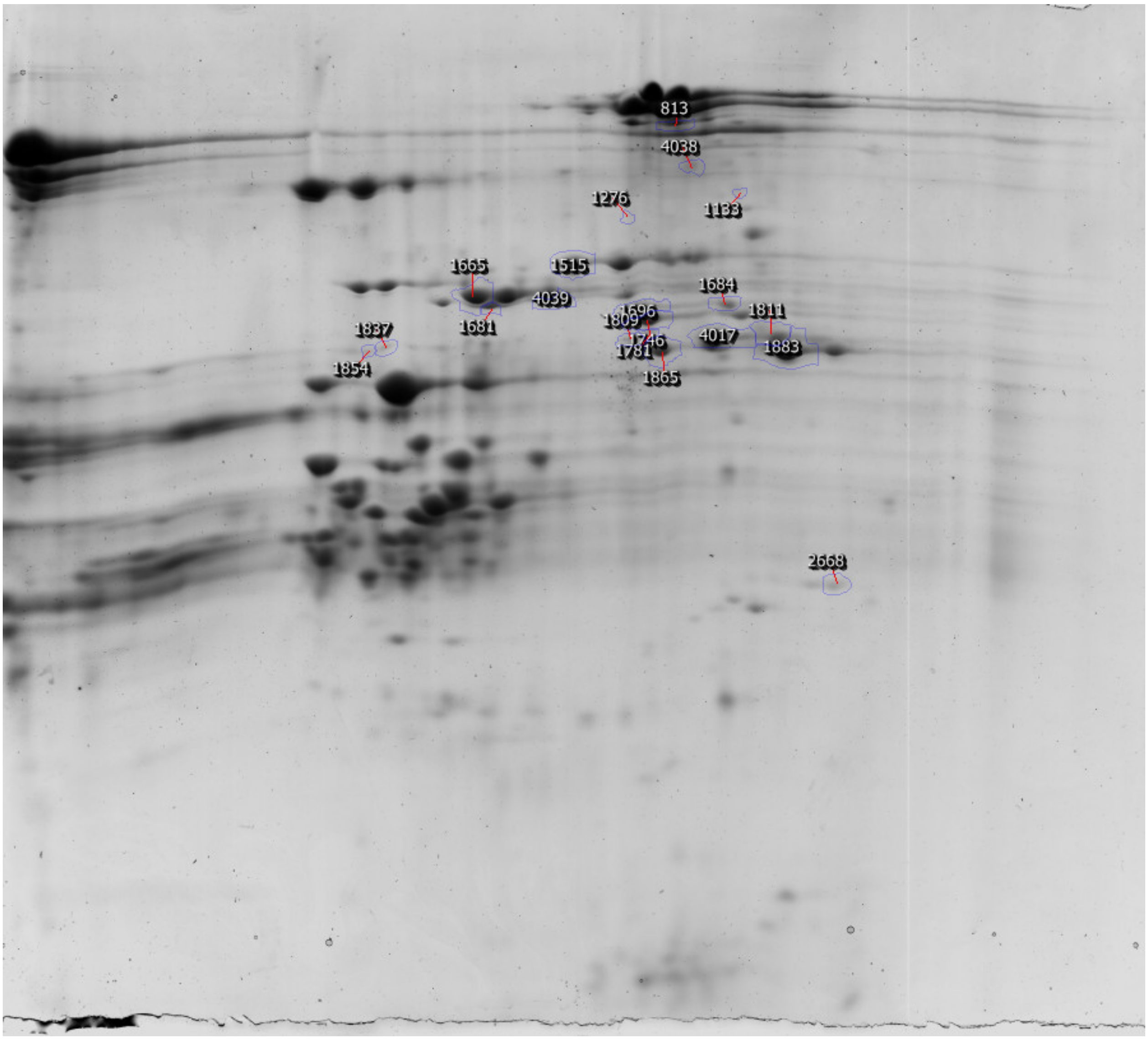

Experiment Design

Condition	Control	Drought
Replicates	9	10

Spots

#	Anova (p)	Fold	Tags	Notes	pI	MW	Protein Accession	Protein Description	Protein pI	Protein MW	Average Normalised Volumes	
											Control	Drought
1276	0,042	3,8									2,701e+005	1,014e+006
1809	0,031	1,7									1,654e+006	2,881e+006
1811	0,003	1,7									9,180e+006	1,568e+007
1865	0,006	1,6									1,167e+007	1,907e+007
1837	0,048	1,6									1,162e+006	1,876e+006
4038	0,029	1,6									1,440e+006	2,303e+006
1781	0,027	1,6									6,389e+005	1,020e+006
1696	0,033	1,6									9,245e+006	1,453e+007
1883	0,003	1,6									3,578e+007	5,607e+007
2668	0,013	1,5									3,172e+006	2,072e+006
1746	0,023	1,5									1,108e+007	1,678e+007
1854	0,025	1,5									4,540e+005	6,773e+005
4039	0,016	1,5									7,442e+006	1,096e+007
1515	0,046	1,4									1,877e+007	2,714e+007
1681	0,036	1,4									3,632e+006	5,223e+006
4017	0,006	1,4									2,736e+007	3,813e+007
813	0,031	1,4									6,944e+006	9,593e+006
1133	0,027	1,4									2,864e+005	3,917e+005
1665	0,049	1,3									2,687e+007	3,572e+007
1684	0,043	1,3									3,497e+006	4,638e+006

Tags	
	Edited
	(COLD)Anova p-value ≤ 0,05
	(COLD)Max fold change ≥ 1,2
	(DROUGHT)Anova p-value ≤ 0,05
	(DROUGHT) Max fold change ≥ 1,2
	(HEAT) Anova p-value ≤ 0,05
	(HEAT) Max fold change ≥ 1,2
	(ALL) Anova p-value ≤ 0,05
	(ALL) Max fold change ≥ 1,2
	(Drought) MAX fold change
	Max fold change ≥ 1,2

Identifier 1276

Position (1307, 447)

Notes

- (ALL) Max fold change ≥ 1,2
- (COLD)Max fold change ≥ 1,2
- (DROUGHT)Anova p-value ≤ 0,05
- (HEAT) Max fold change ≥ 1,2
- Max fold change ≥ 1,2
- Max fold change ≥ 1,2

Control	Drought
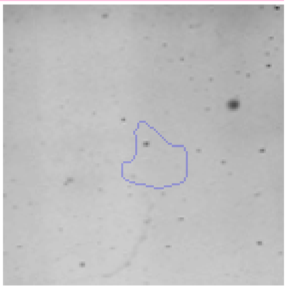	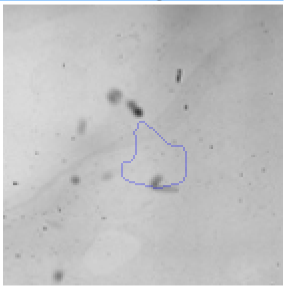

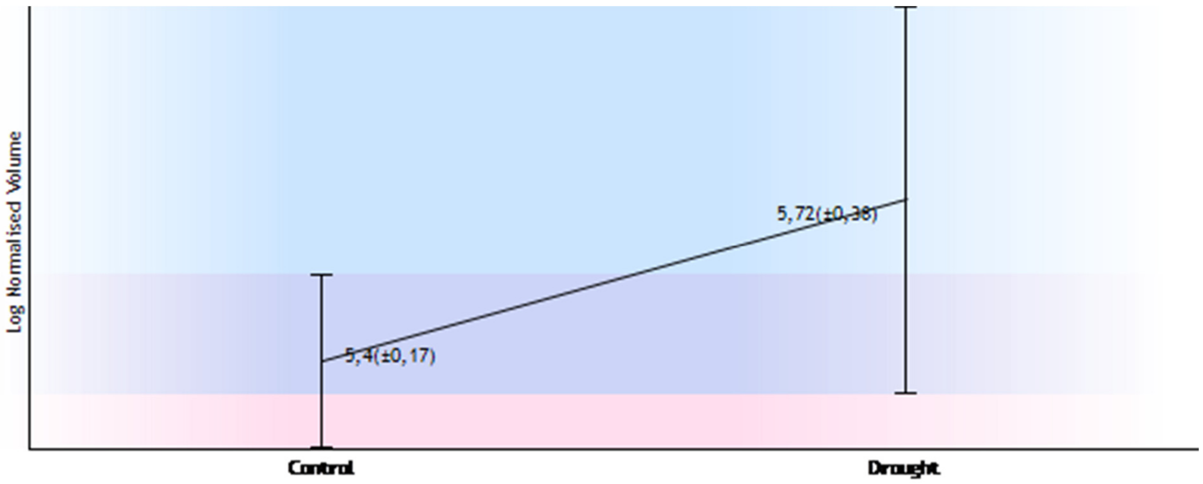

Identifier 1809

Position (1324, 705)

Notes

- (ALL) Max fold change ≥ 1,2
- (DROUGHT)Anova p-value ≤ 0,05
- (HEAT) Max fold change ≥ 1,2
- Max fold change ≥ 1,2
- (DROUGHT) Max fold change ≥ 1,2

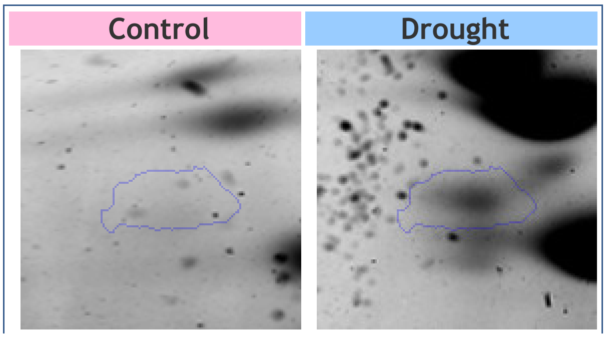

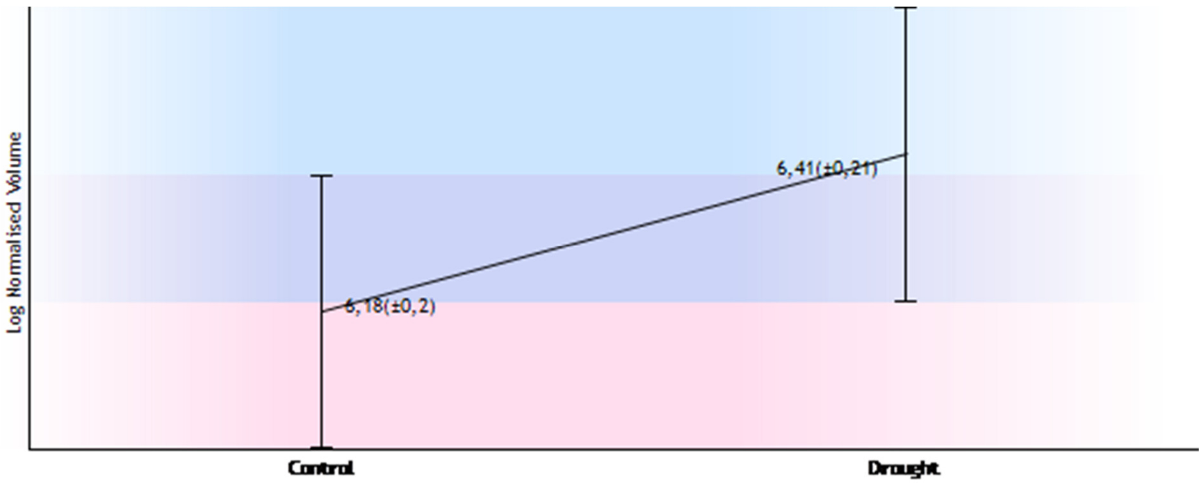

Identifier 1811

Position (1624, 707)

Notes

- (ALL) Anova p-value ≤ 0,05
- (ALL) Max fold change ≥ 1,2
- (COLD)Anova p-value ≤ 0,05
- (COLD)Max fold change ≥ 1,2
- (DROUGHT)Anova p-value ≤ 0,05
- (HEAT) Max fold change ≥ 1,2
- (DROUGHT) Max fold change ≥ 1,2

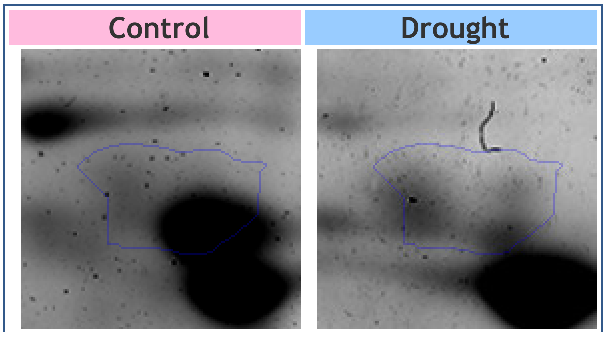

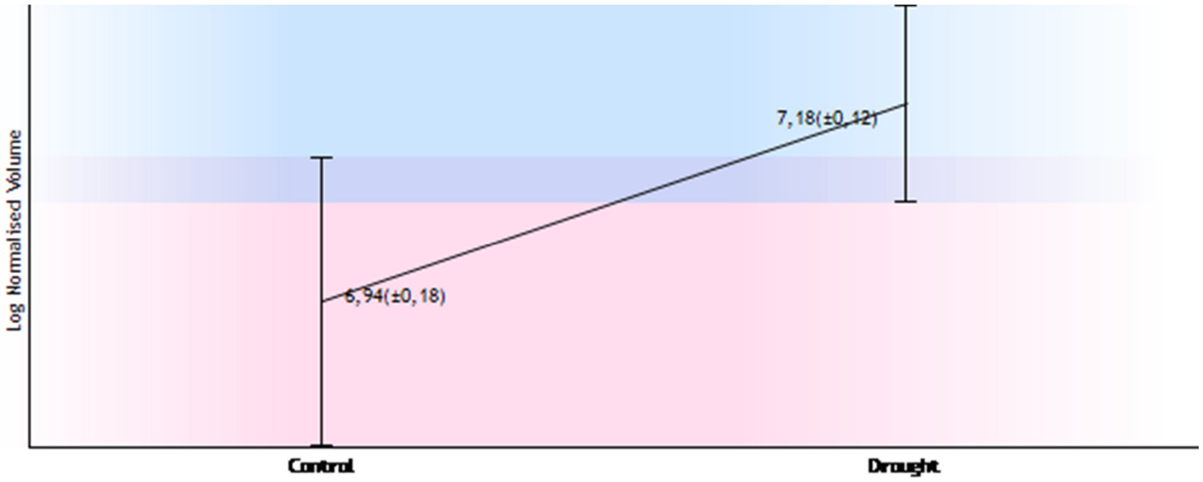

Identifier 1865

Position (1380, 731)

Notes

- (ALL) Max fold change ≥ 1,2
- (DROUGHT)Anova p-value ≤ 0,05
- (DROUGHT) Max fold change ≥ 1,2

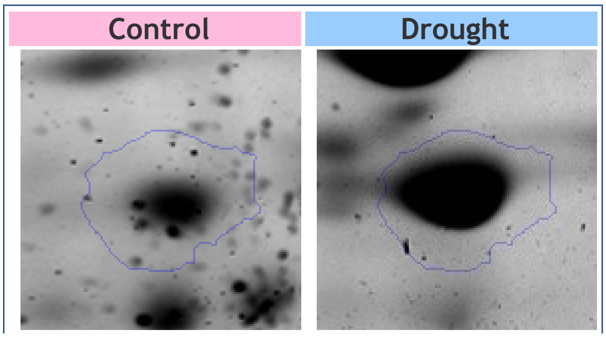

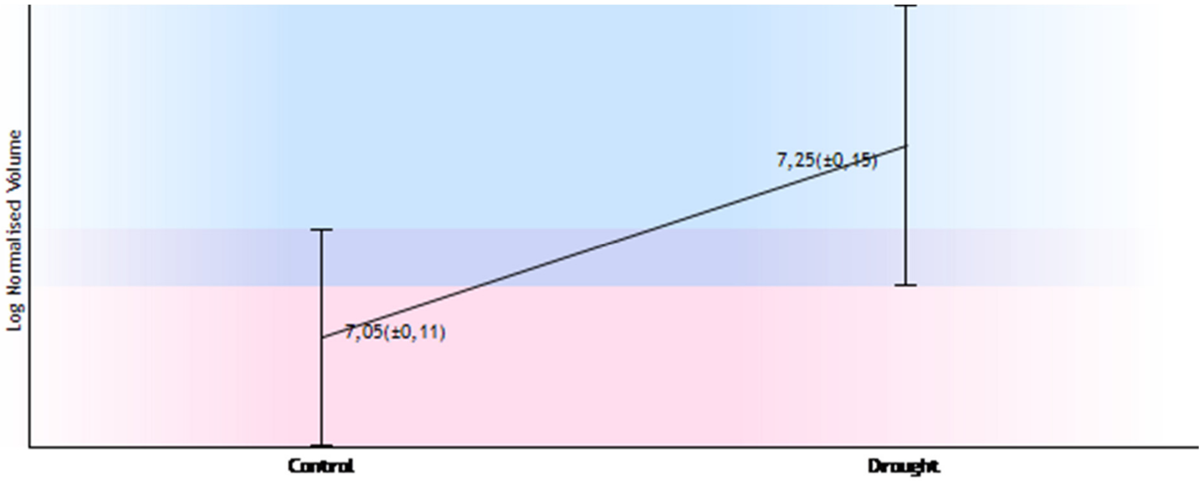

Identifier 1837

Position (804, 719)

Notes

- (ALL) Max fold change ≥ 1,2
- (COLD)Max fold change ≥ 1,2
- (DROUGHT)Anova p-value ≤ 0,05
- (DROUGHT) Max fold change ≥ 1,2

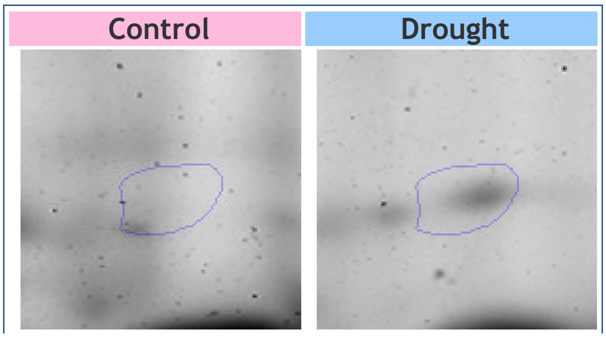

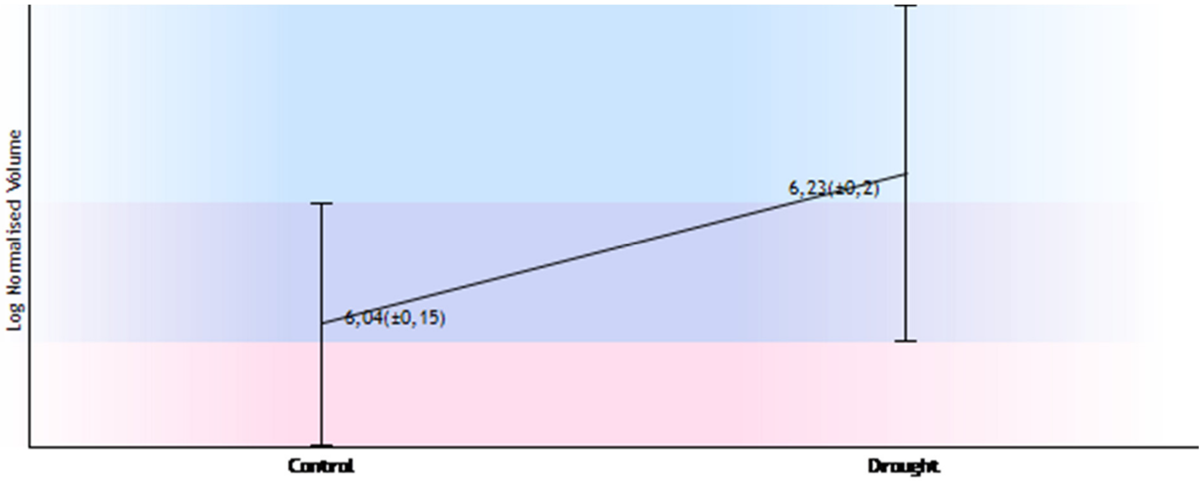

Identifier 4038

Position (1460, 349)

Notes

- (ALL) Max fold change ≥ 1,2
- (COLD)Anova p-value ≤ 0,05
- (COLD)Max fold change ≥ 1,2
- (DROUGHT)Anova p-value ≤ 0,05
- (HEAT) Max fold change ≥ 1,2
- Edited
- (DROUGHT) Max fold change ≥ 1,2

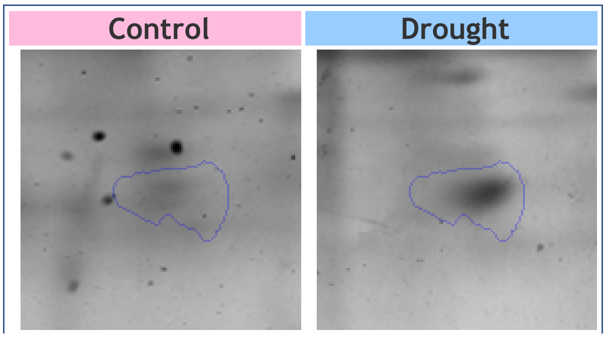

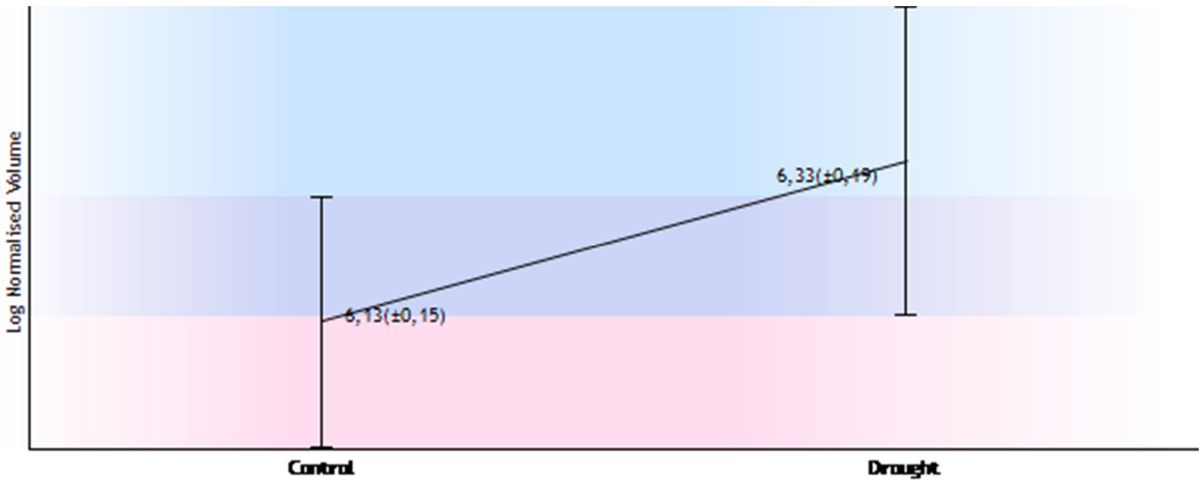

Identifier 1781

Position (1357, 688)

Notes

- (ALL) Max fold change ≥ 1,2
- (DROUGHT)Anova p-value ≤ 0,05
- (HEAT) Max fold change ≥ 1,2
- Max fold change ≥ 1,2

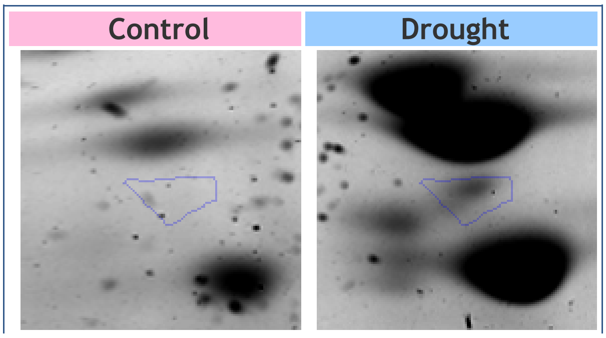

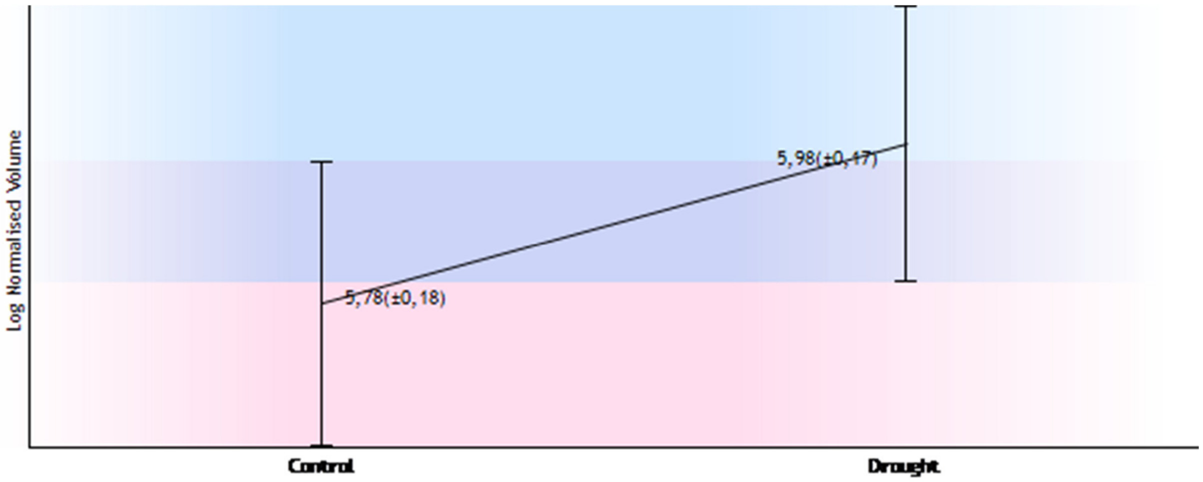

Identifier 1696

Position (1331, 646)

Notes

- (ALL) Max fold change ≥ 1,2
- (DROUGHT)Anova p-value ≤ 0,05
- (HEAT) Max fold change ≥ 1,2
- (DROUGHT) Max fold change ≥ 1,2

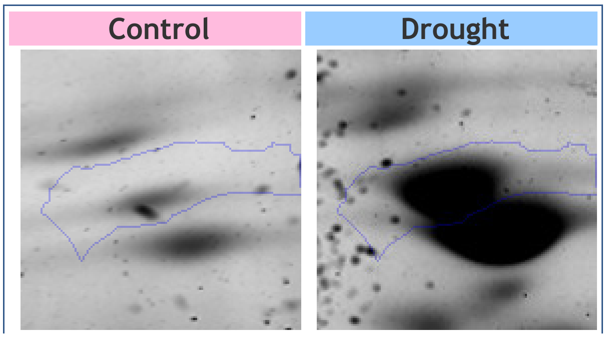

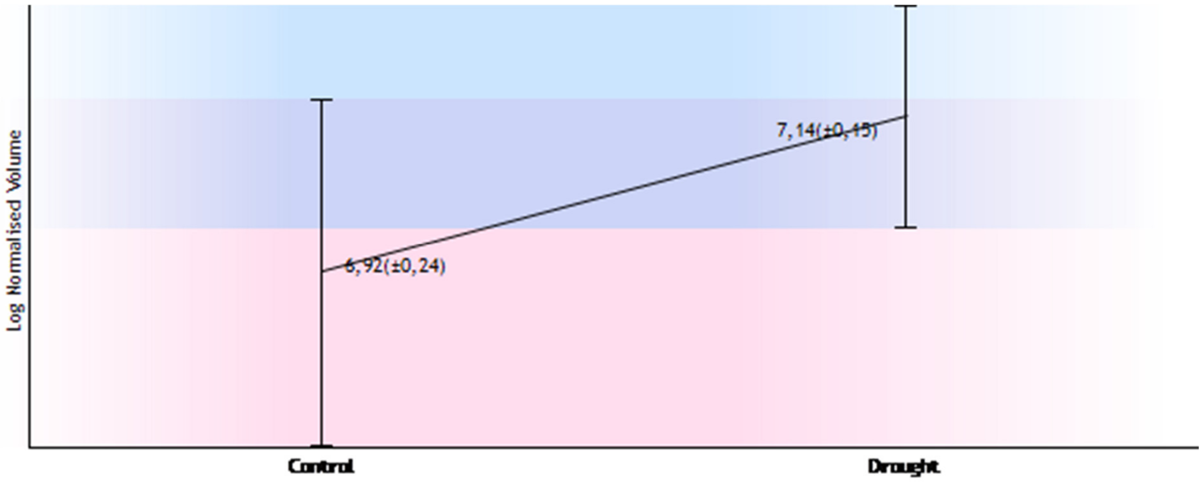

Identifier 1883

Position (1643, 740)

Notes

- (ALL) Anova p-value ≤ 0,05
- (ALL) Max fold change ≥ 1,2
- (DROUGHT)Anova p-value ≤ 0,05
- (DROUGHT) Max fold change ≥ 1,2

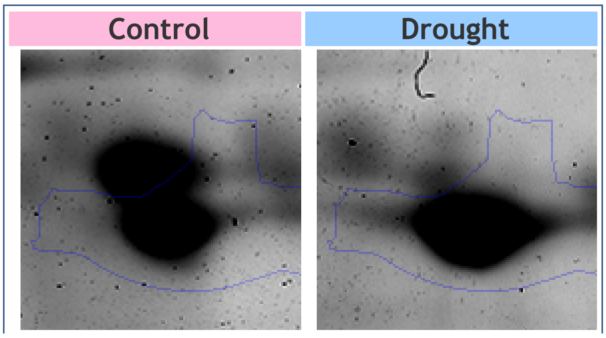

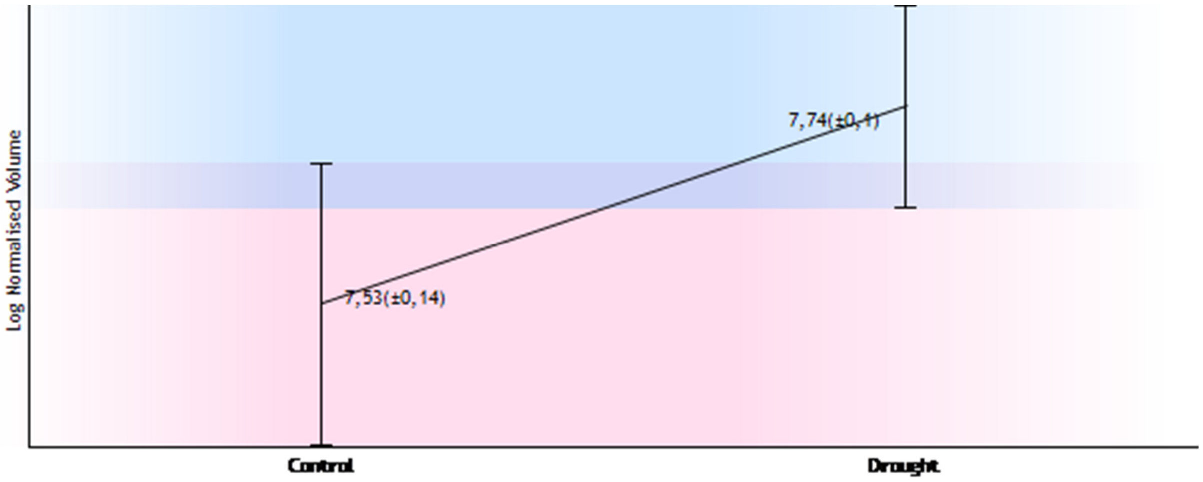

Identifier 2668

Position (1743, 1220)

Notes

- (ALL) Max fold change ≥ 1,2
- (DROUGHT)Anova p-value ≤ 0,05
- (DROUGHT) Max fold change ≥ 1,2

Identifier 1746

Position (1355, 667)

Notes

- (ALL) Max fold change ≥ 1,2
- (COLD)Max fold change ≥ 1,2
- (DROUGHT)Anova p-value ≤ 0,05
- (DROUGHT) Max fold change ≥ 1,2

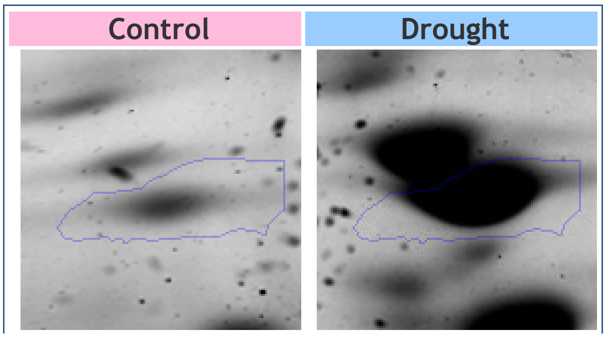

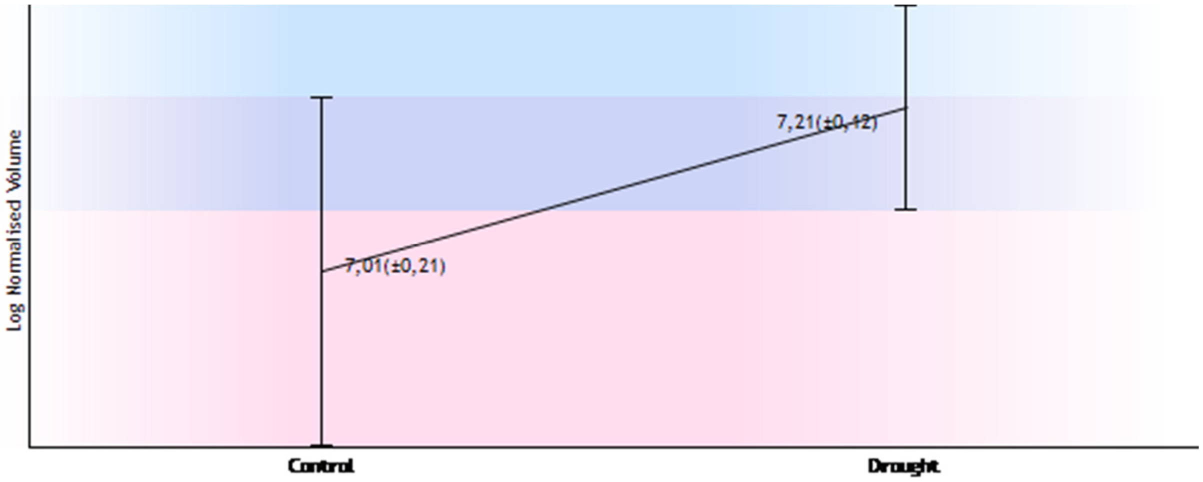

Identifier 1854

Position (764, 727)

Notes

- (ALL) Max fold change ≥ 1,2
- (COLD)Max fold change ≥ 1,2
- (DROUGHT)Anova p-value ≤ 0,05
- (DROUGHT) Max fold change ≥ 1,2

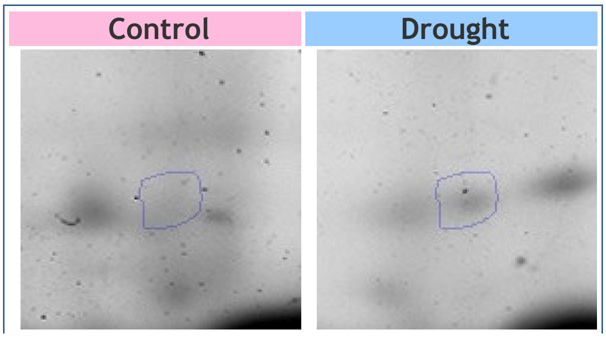

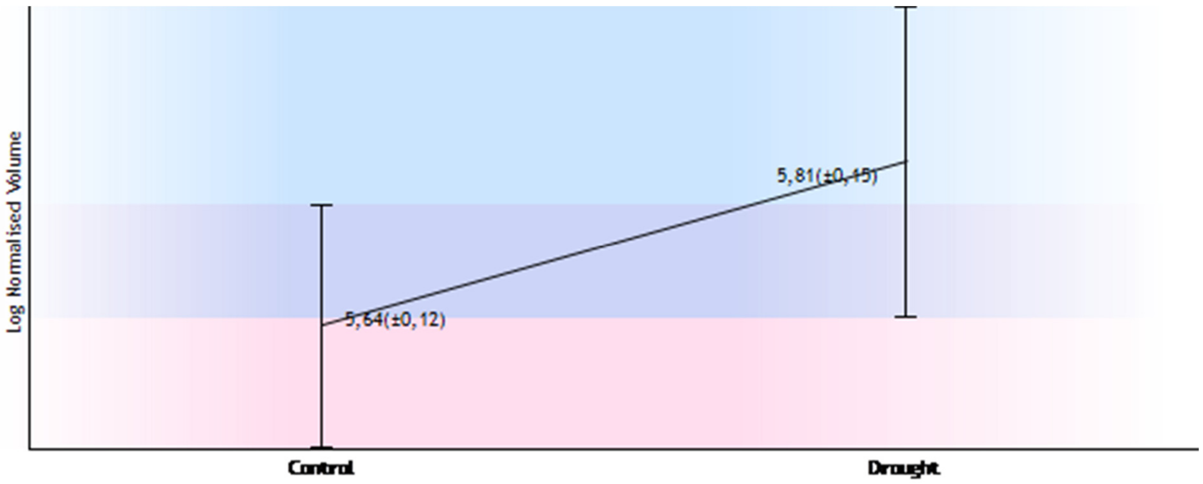

Identifier 4039

Position (1149, 617)

Notes

- (ALL) Max fold change ≥ 1,2
- (COLD)Max fold change ≥ 1,2
- (DROUGHT)Anova p-value ≤ 0,05
- (HEAT) Max fold change ≥ 1,2
- Edited
- (DROUGHT) Max fold change ≥ 1,2

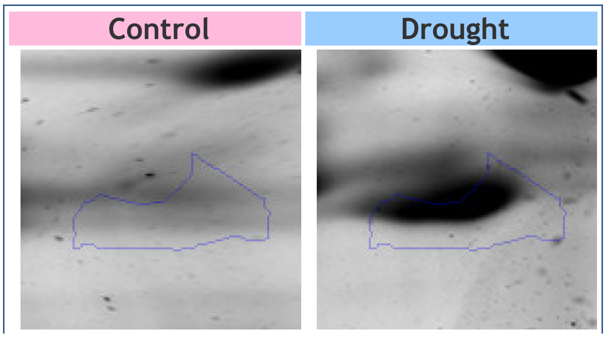

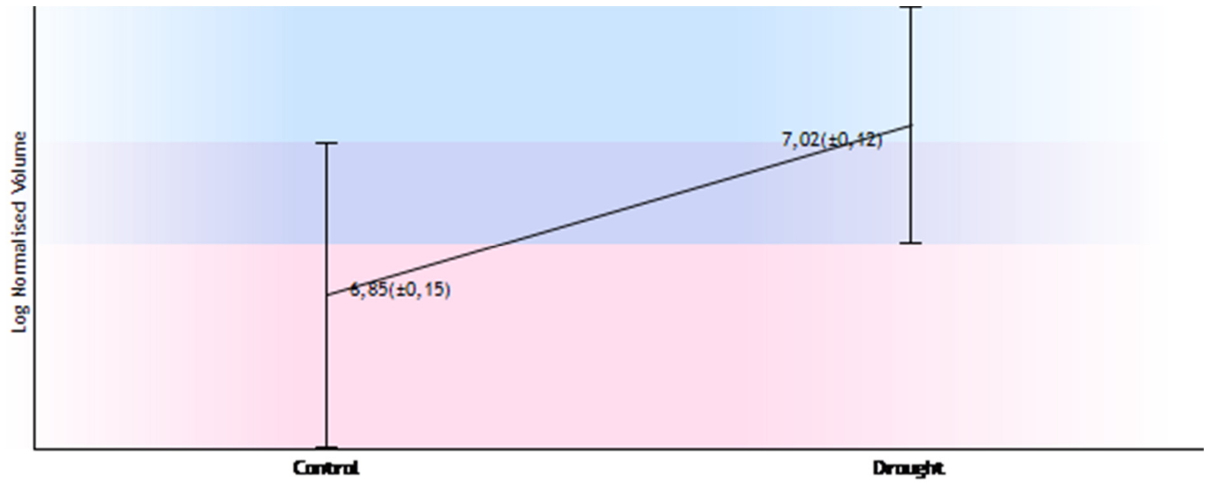

Identifier 1515

Position (1200, 556)

Notes

- (ALL) Max fold change ≥ 1,2
- (DROUGHT)Anova p-value ≤ 0,05
- (DROUGHT) Max fold change ≥ 1,2

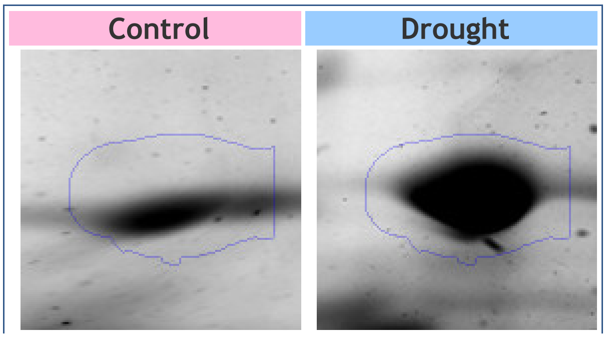

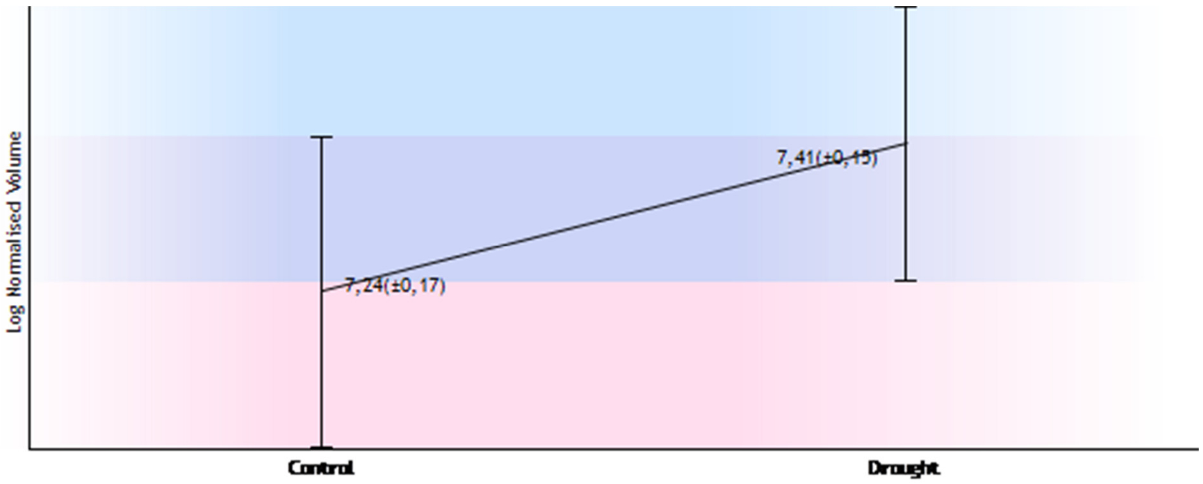

Identifier 1681

Position (1022, 633)

Notes

- (ALL) Max fold change ≥ 1,2
- (COLD)Max fold change ≥ 1,2
- (DROUGHT)Anova p-value ≤ 0,05
- (DROUGHT) Max fold change ≥ 1,2

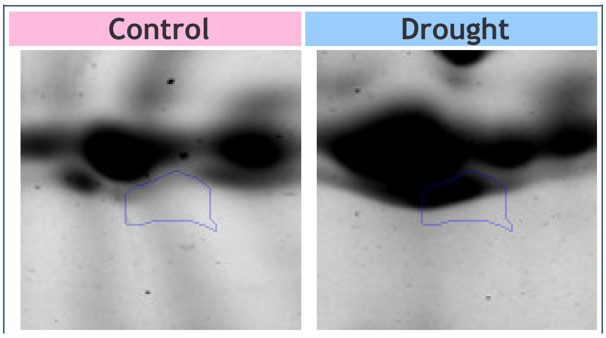

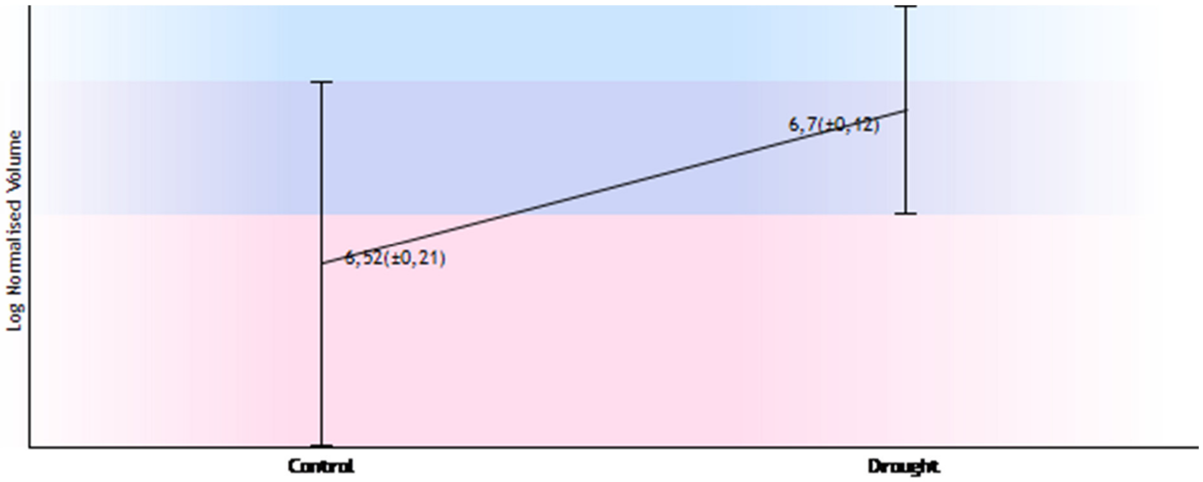

Identifier 4017

Position (1498, 702)

Notes

- (ALL) Anova p-value ≤ 0,05
- (ALL) Max fold change ≥ 1,2
- (DROUGHT)Anova p-value ≤ 0,05
- Edited
- (DROUGHT) Max fold change ≥ 1,2

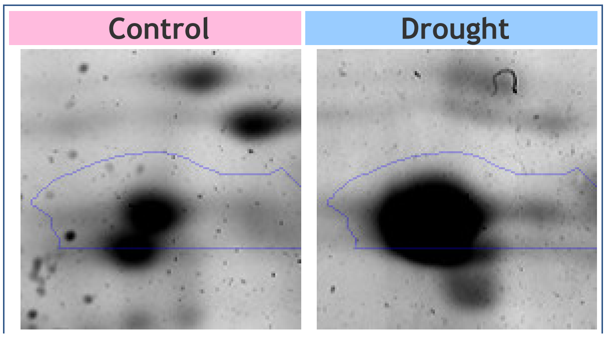

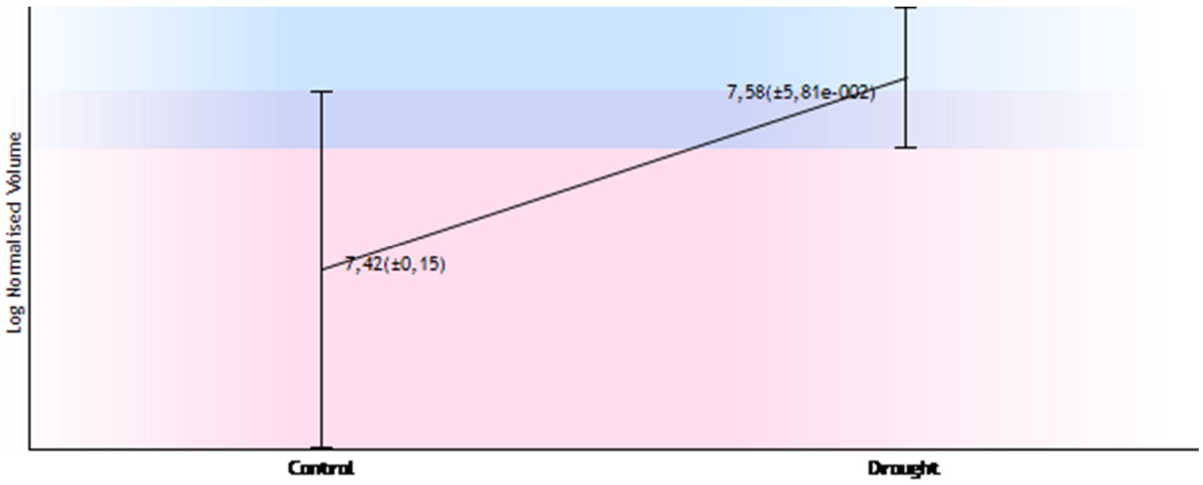

Identifier 813

Position (1412, 250)

Notes

- (ALL) Max fold change ≥ 1,2
- (COLD)Max fold change ≥ 1,2
- (DROUGHT)Anova p-value ≤ 0,05
- (DROUGHT) Max fold change ≥ 1,2

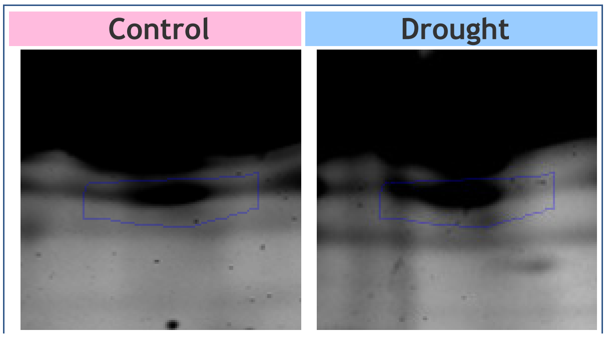

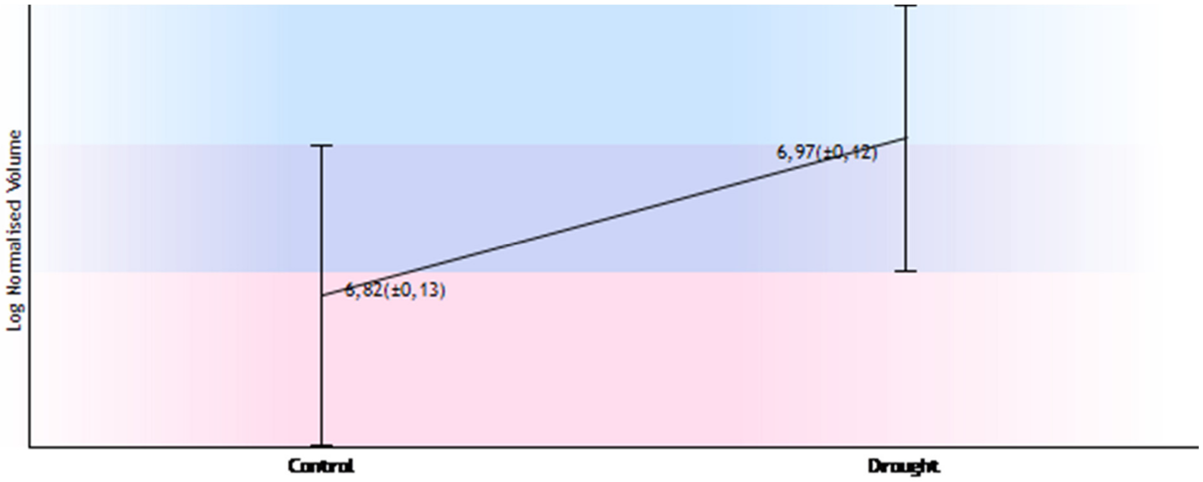

Identifier 1133

Position (1545, 389)

Notes

- (ALL) Max fold change ≥ 1,2
- (COLD)Max fold change ≥ 1,2
- (DROUGHT)Anova p-value ≤ 0,05
- (DROUGHT) Max fold change ≥ 1,2

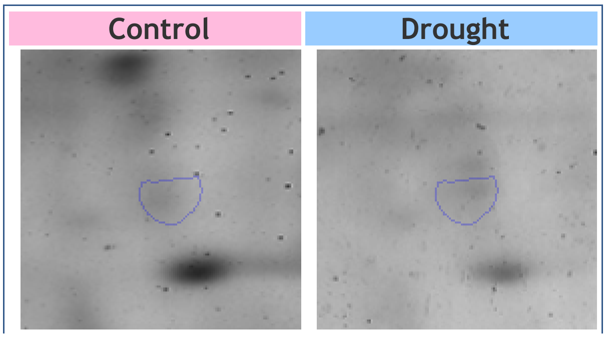

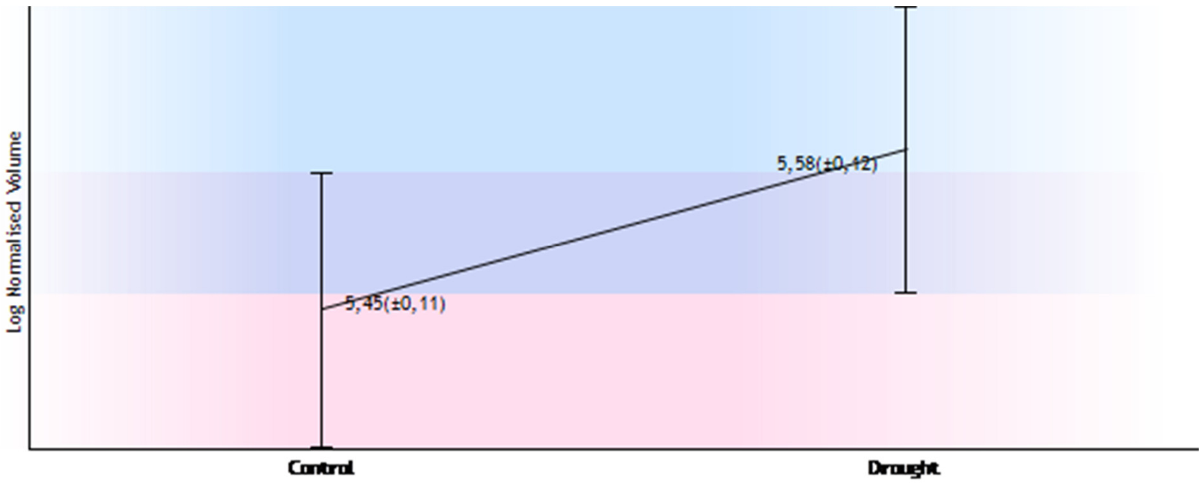

Identifier 1665

Position (994, 622)

Notes

- (ALL) Anova p-value ≤ 0,05
- (ALL) Max fold change ≥ 1,2
- (COLD)Max fold change ≥ 1,2
- (DROUGHT)Anova p-value ≤ 0,05
- (DROUGHT) Max fold change ≥ 1,2

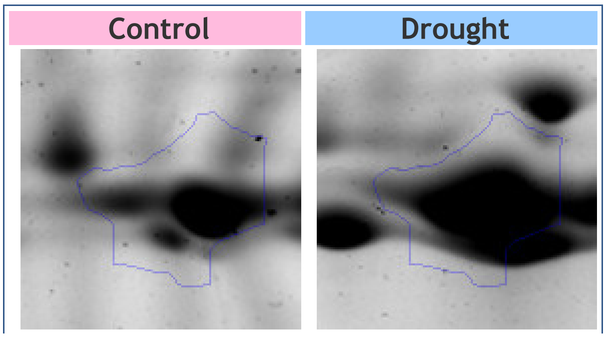

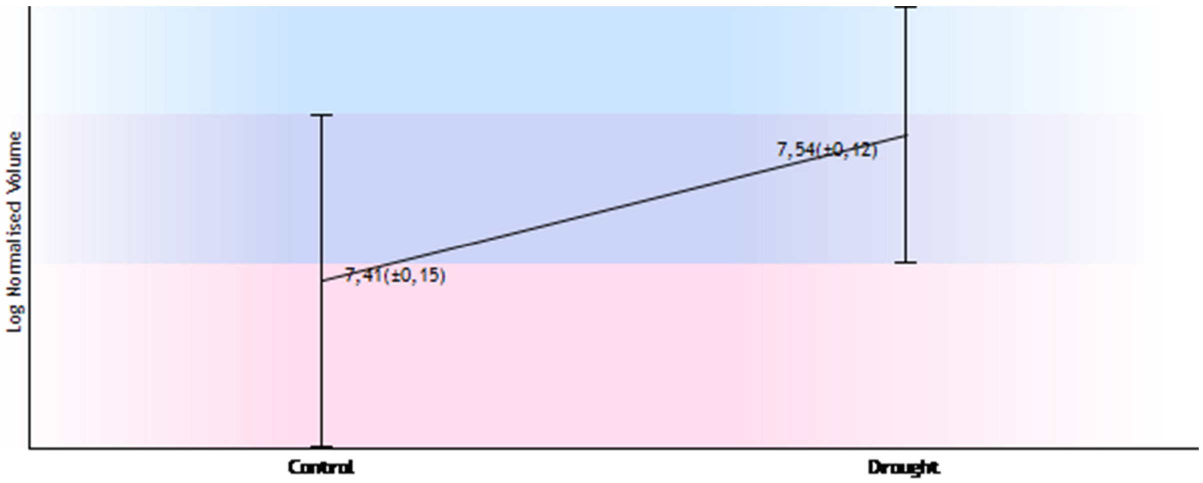

Identifier 1684

Position (1521, 633)

Notes

- (ALL) Max fold change ≥ 1,2
- (COLD)Max fold change ≥ 1,2
- (DROUGHT)Anova p-value ≤ 0,05
- (HEAT) Max fold change ≥ 1,2
- (DROUGHT) Max fold change ≥ 1,2

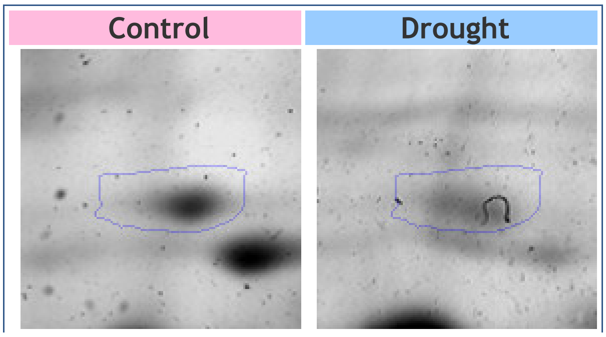

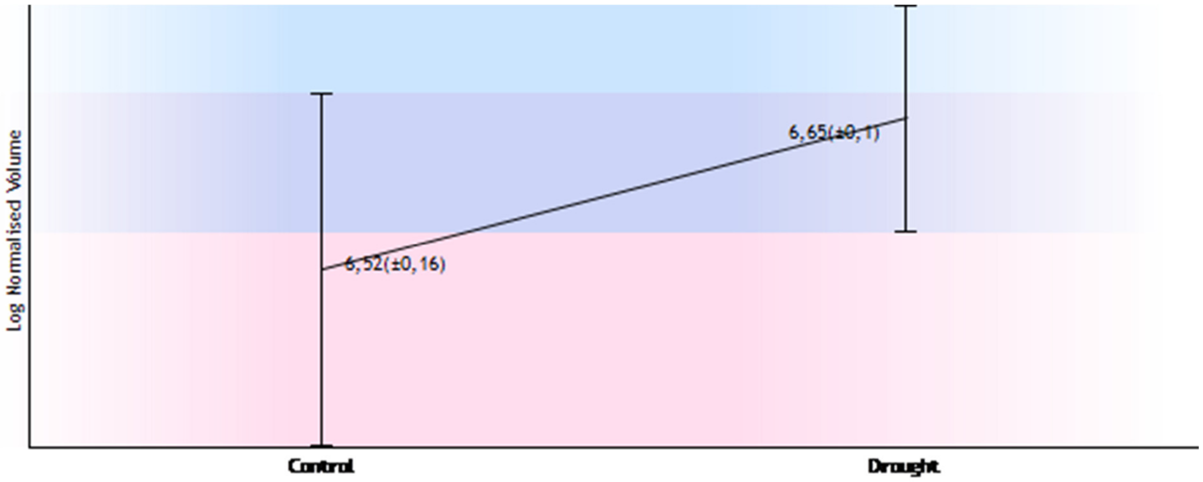

Statistics performed considering Cold/Drought treatment

Reference image

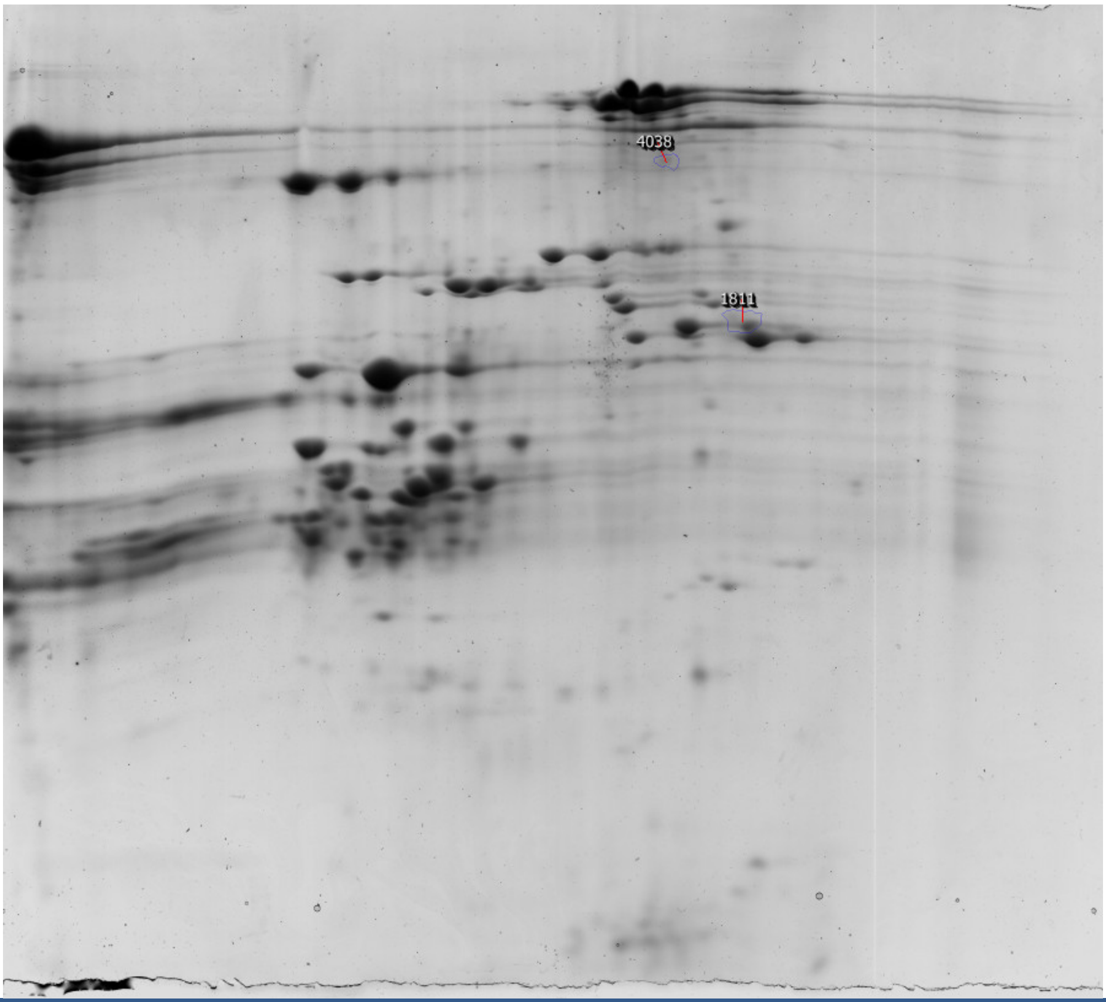

Experiment Design

Condition	Control	Drought
Replicates	9	10

Spots

#	Anova (p)	Fold	Tags	Notes	pI	MW	Protein Accession	Protein Description	Protein pI	Protein MW	Average Normalised Volumes	
											Control	Drought
4038	0,029	1,6									1,440e+006	2,303e+006
1811	0,003	1,7									9,180e+006	1,568e+007

Tags	
	Edited
	(COLD)Anova p-value ≤ 0,05
	(COLD)Max fold change ≥ 1,2

	(DROUGHT)Anova p-value ≤ 0,05
	(DROUGHT) Max fold change ≥ 1,2
	(HEAT) Anova p-value ≤ 0,05
	(HEAT) Max fold change ≥ 1,2
	(ALL) Anova p-value ≤ 0,05
	(ALL) Max fold change ≥ 1,2
	(Drought) MAX fold change

Identifier 4038

Position (1460, 349)

Notes

- (ALL) Max fold change ≥ 1,2
- (COLD)Anova p-value ≤ 0,05
- (COLD)Max fold change ≥ 1,2
- (DROUGHT)Anova p-value ≤ 0,05
- (HEAT) Max fold change ≥ 1,2
- Edited
- (DROUGHT) Max fold change ≥ 1,2

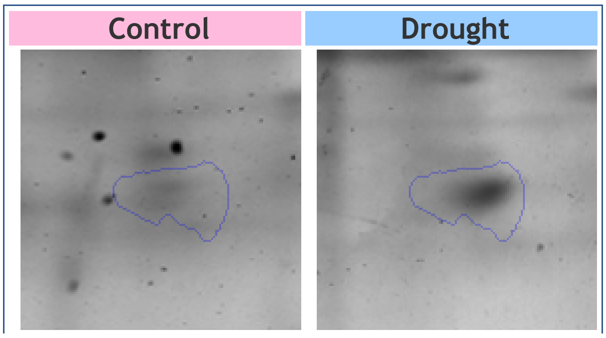

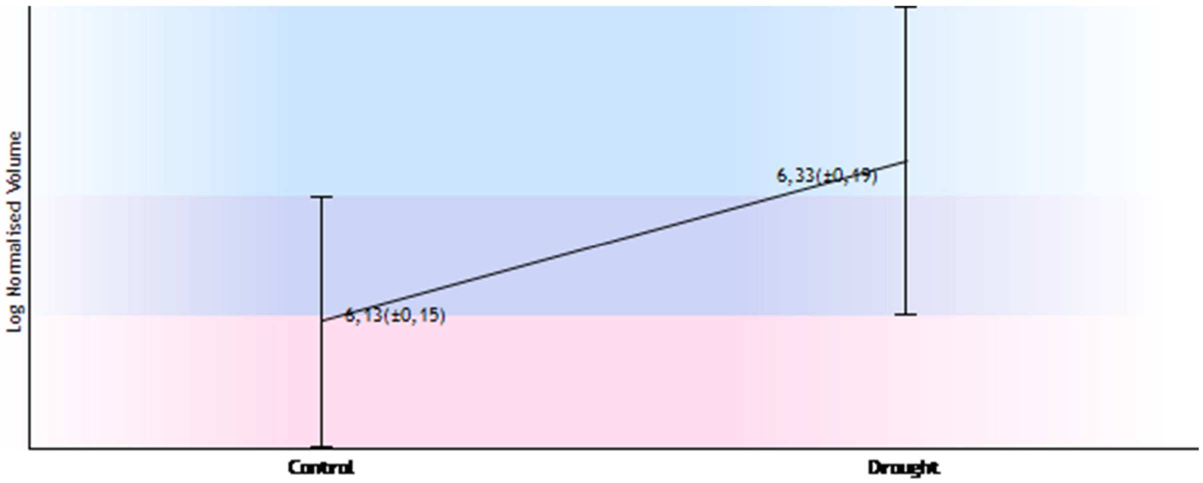

Identifier 1811

Position (1624, 707)

Notes

- (ALL) Anova p-value ≤ 0,05
- (ALL) Max fold change ≥ 1,2
- (COLD)Anova p-value ≤ 0,05
- (COLD)Max fold change ≥ 1,2
- (DROUGHT)Anova p-value ≤ 0,05
- (HEAT) Max fold change ≥ 1,2
- (DROUGHT) Max fold change ≥ 1,2

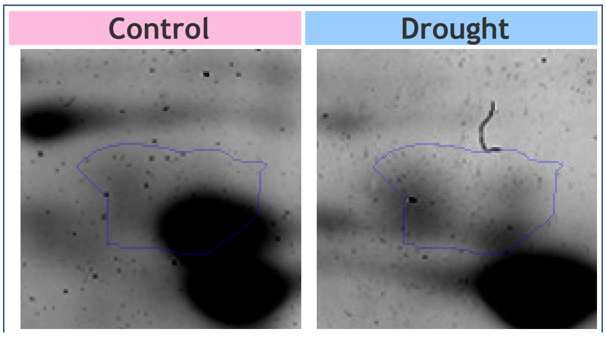

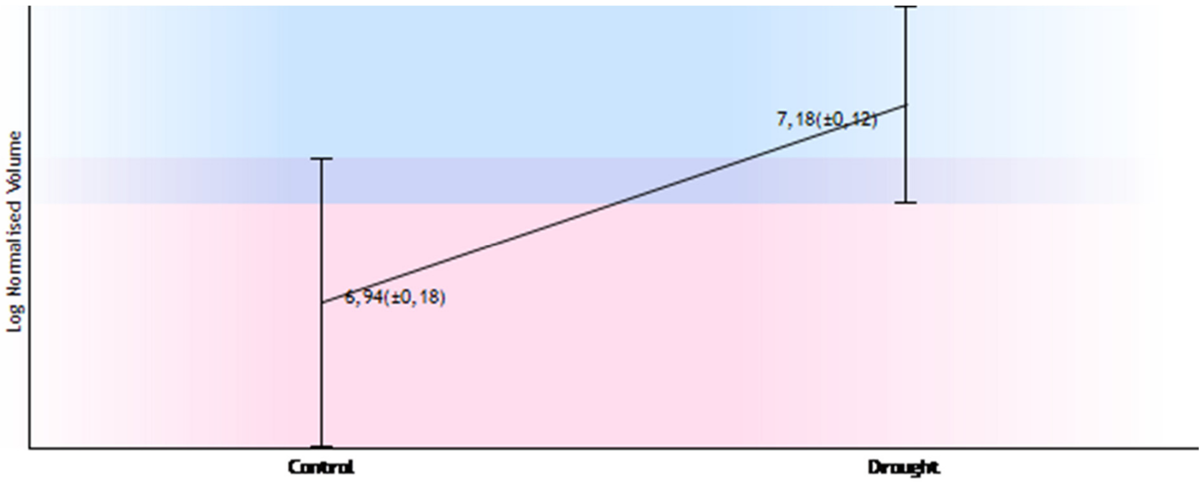

Reference image

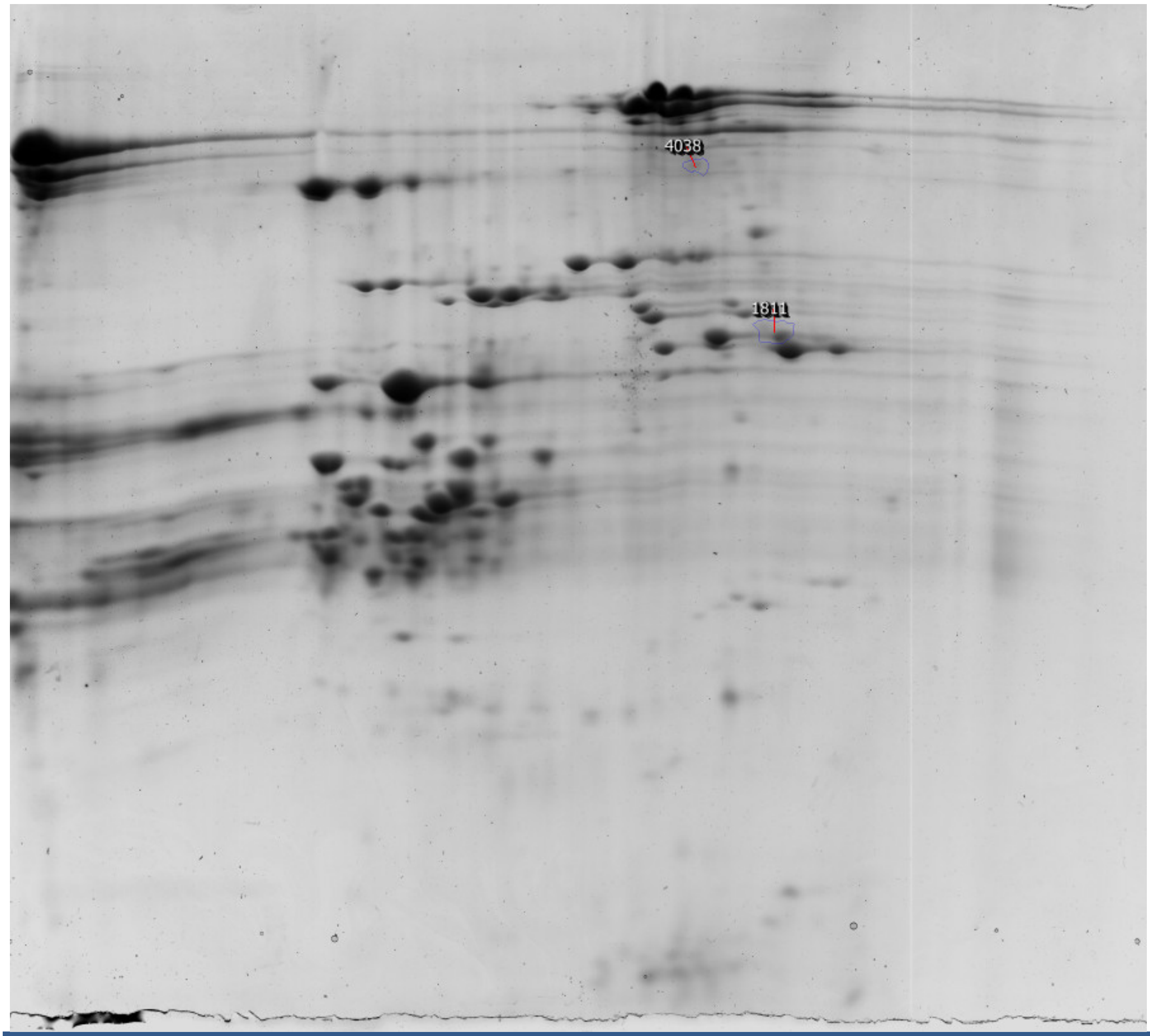

Experiment Design

Condition	Control	Cold
Replicates	9	9

Spots

#	Anova (p)	Fold	Tags	Notes	pI	MW	Protein Accession	Protein Description	Protein pI	Protein MW	Average Normalised Volumes	
											Control	Cold
4038	0,019	1,6									1,440e+006	2,346e+006
1811	0,032	1,4									9,180e+006	1,295e+007

Tags

	Edited
	(COLD)Anova p-value ≤ 0,05
	(COLD)Max fold change ≥ 1,2
	(DROUGHT)Anova p-value ≤ 0,05
	(DROUGHT) Max fold change ≥ 1,2
	(HEAT) Anova p-value ≤ 0,05
	(HEAT) Max fold change ≥ 1,2
	(ALL) Anova p-value ≤ 0,05
	(ALL) Max fold change ≥ 1,2
	(Drought) MAX fold change

Identifier 4038

Position (1460, 349)

Notes

- (ALL) Max fold change ≥ 1,2
- (COLD)Anova p-value ≤ 0,05
- (COLD)Max fold change ≥ 1,2
- (DROUGHT)Anova p-value ≤ 0,05
- (HEAT) Max fold change ≥ 1,2
- Edited
- (DROUGHT) Max fold change ≥ 1,2

Control	Cold

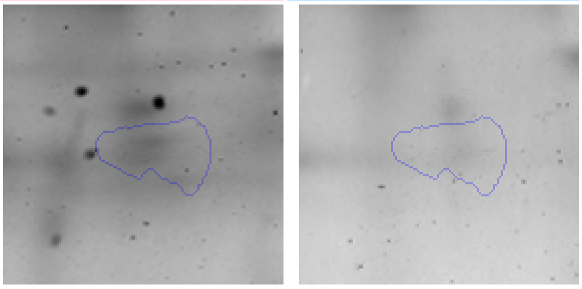

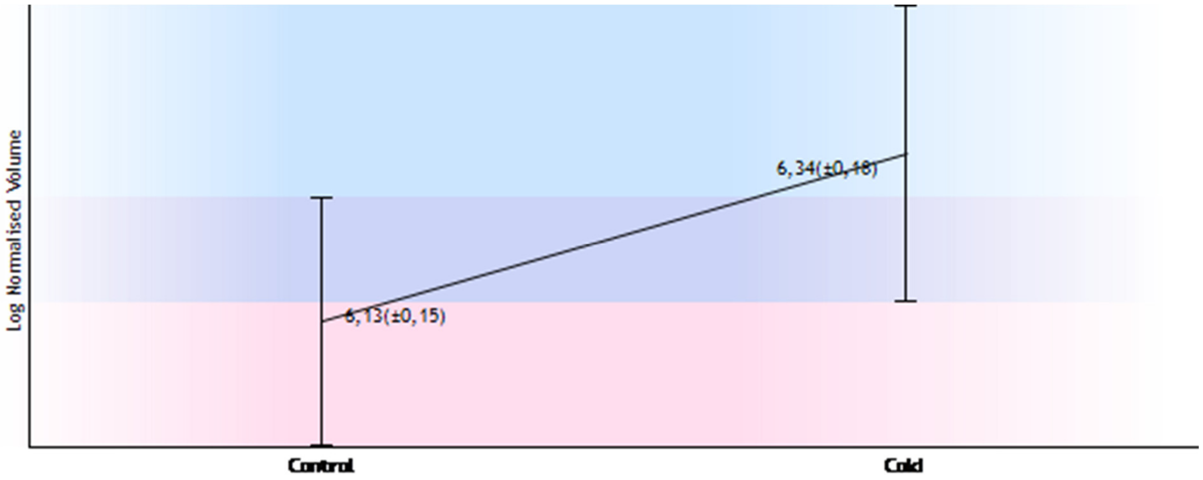

Identifier 1811

Position (1624, 707)

Notes

- (ALL) Anova p-value ≤ 0,05
- (ALL) Max fold change ≥ 1,2
- (COLD)Anova p-value ≤ 0,05
- (COLD)Max fold change ≥ 1,2
- (DROUGHT)Anova p-value ≤ 0,05
- (HEAT) Max fold change ≥ 1,2
- (DROUGHT)Max fold change ≥ 1,2

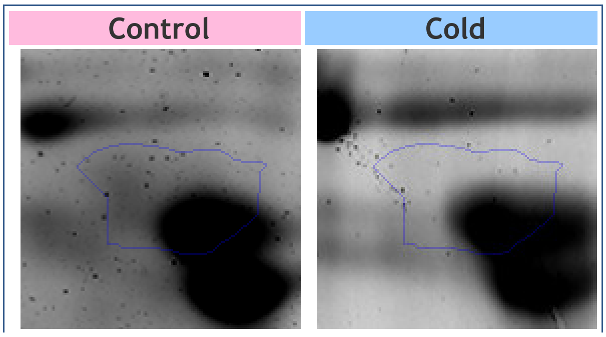

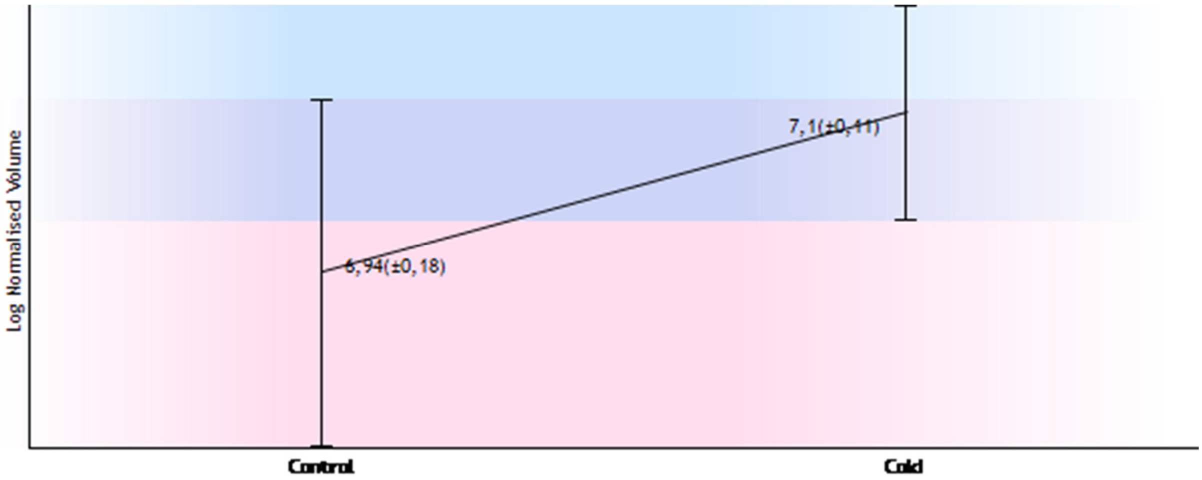

No spots differentially espressed in heat treatment

Figure S1. Raw data of analyzed protein spots after cold and drought treatments.
